# Rare *LRRFIP1* variants contribute to the pathophysiology of severe early-onset scoliosis and common scoliotic phenotypes

**DOI:** 10.1172/JCI202751

**Published:** 2026-09-29

**Authors:** Tanja Frey, Elena M. Cabello, Gabriele Siegel, Carla Bello, Rike Schiller, Martina A. Trippel, Neguin Ranjbar, Paranchai Boonsawat, Stephanie E. van Gijn, Ivan Ivanovski, Michael Papik, Markus Zweier, Kan Min, Katharina Steindl, Anita Rauch

**Affiliations:** 1 Institute of Medical Genetics, University of Zurich, Schlieren-Zurich, Switzerland.; 2 University Children’s Hospital Zurich, University of Zurich, Zurich, Switzerland.; 3 Swiss Scoliosis, Centre for spinal and scoliosis surgery, Zurich, Switzerland.

**Keywords:** Clinical Research, Genetics, Extracellular matrix, Genetic diseases, Rheumatology

## Abstract

Despite enormous advances in clinical genomics, idiopathic scoliosis remains an enigmatic condition with poorly understood genetic and pathophysiological underpinnings impeding molecular diagnosis and the development of targeted treatments. We performed linkage analysis, exome, genome and short- and long-read RNA-seq in a multigenerational family affected by autosomal dominant early-onset scoliosis (EOS) with a unique pattern of spondylodysplastic elements and progressive endplate erosion and identified the LRR-binding FLII-interacting protein 1 (*LRRFIP1*) gene as the disease-causing gene. The underlying cause is a rare noncoding variant altering transcription factor binding of nuclear receptor subfamily 3 group C member 1 (NR3C1) (glucocorticoid receptor), leading to changes in *LRRFIP1* isoform expression. Transcriptomic changes in fibroblasts of affected individuals indicated a combination of disturbed Wnt signaling during somitogenesis, planar cell polarity signaling, and postnatal inflammatory dysregulation with clinical and molecular overlaps with ankylosing spondylitis and Scheuermann’s kyphosis. We conducted a rare variant enrichment analysis using genome data from 484,903 UK Biobank participants and found an enrichment of rare risk-increasing *LRRFIP1* variants in individuals with scoliosis. Our analysis of an Lrrfip1^tm1.1(KOMP)Wtsi^-KO mouse model showed an increased prevalence of idiopathic kyphoscoliosis in *Lrrfip1*-deficient mice. Our work provides insights into the pathophysiology of rare and common spinal disorders and hints at potential future therapeutic approaches with selective NR3C1 inhibitors.

## Abstract 2

Research involving a large family with severe childhood-onset scoliosis identified a genetic cause linked to altered development and inflammatory processes, informing future therapeutic approaches.

## Graphical abstract

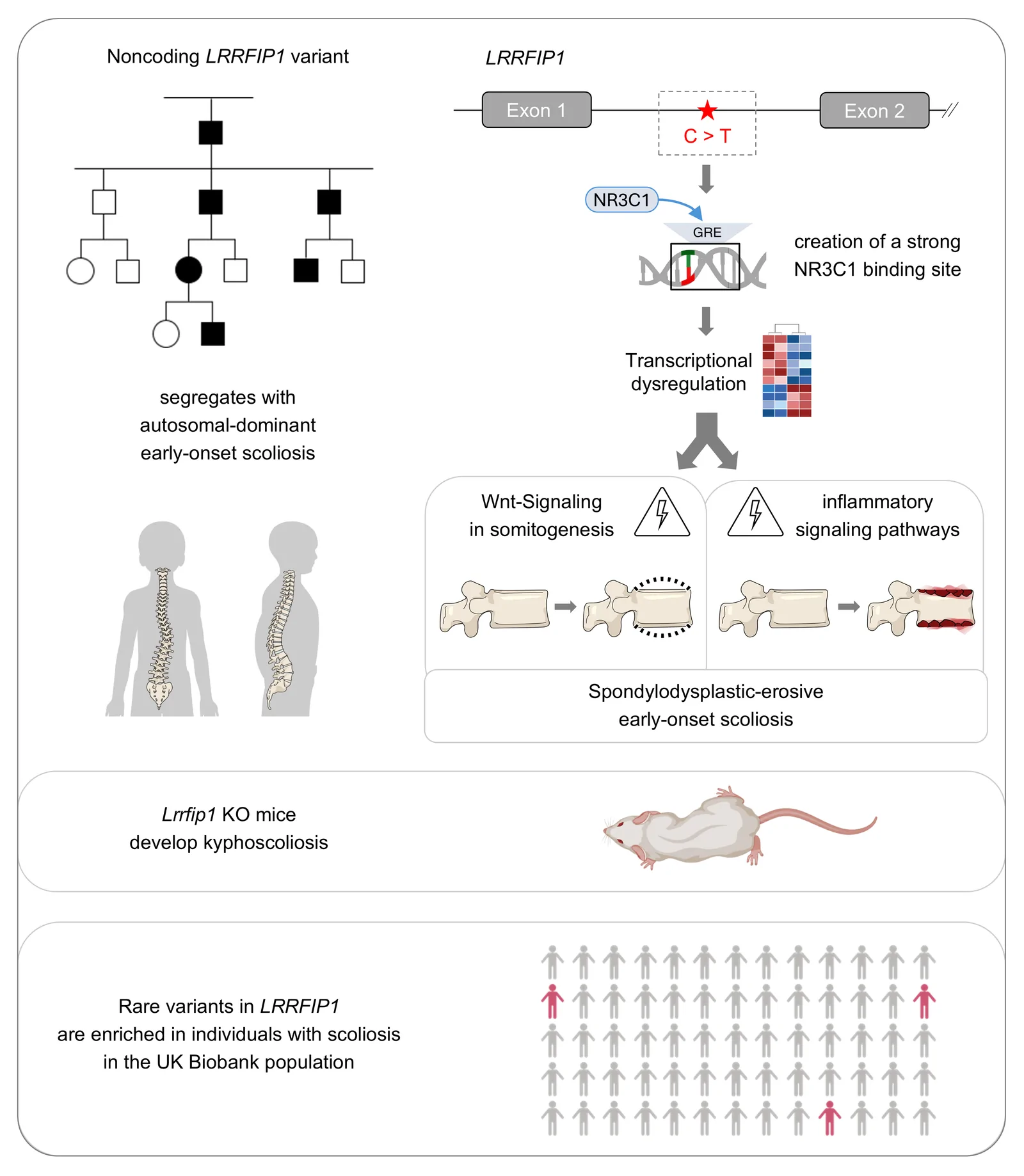

## Introduction

Scoliosis is a common condition affecting over 10% of the adult population above age 40 (1), and 1%–3% of adolescents develop scoliosis without any apparent cause (adolescent idiopathic scoliosis) (2). Despite extensive research, the etiology of adolescent idiopathic scoliosis remains obscure and is most likely the result of a complex interplay of genetic, biomechanical, environmental, and hormonal factors (2). The rarer early-onset scoliosis (EOS) with onset before age 10 years can be part of a multisystem disorder (neuromuscular EOS and syndromic EOS) or occur in isolation (congenital scoliosis or idiopathic EOS) (3) and often leads to lifelong sequelae and disability. Idiopathic EOS has a prevalence of approximately 1 in 3,500 individuals and is defined as a spinal deformity of over 10° presenting in children under the age of 10 years without an underlying vertebral segmentation or formation defect or syndromic or neuromuscular condition (3).

Although a primarily monogenic cause is suspected for many forms of EOS, the genetic diagnostic rate is low. In a large EOS cohort, a monogenic cause was identified in 18.6% of individuals, most of which were affected by syndromic or neuromuscular forms of EOS (4). For isolated forms of EOS, however, only a few causative genes have been described to date, including *TBX6* (5) and *PTK7* (6), which only explain a fraction of isolated EOS cases (4) and implicate disturbed Notch, Wnt, and planar cell polarity (PCP) signaling in the etiology (7). Most patients with isolated EOS, however, currently remain without a molecular diagnosis (4).

Among other factors, the considerable number of genes with still unknown disease association and the difficulties in interpreting variants in noncoding regions contribute to a limited diagnostic yield (8). The identification of new disease genes and a better understanding of noncoding variations are important steps toward higher diagnostic yields, which will not only improve patient management but also provide insights into the molecular pathomechanisms, a fundamental prerequisite for the development of targeted therapies. Importantly, the identification of genetic causes of rare diseases like EOS also has the potential to provide insight into the mechanisms underlying more common conditions (9).

Here, we identified a very rare noncoding variant in the LRR-binding, FLII-interacting protein 1 gene (*LRRFIP1),* which, to our knowledge, has not previously been established as a monogenic disease gene, as the cause of a severe and distinctive form of autosomal-dominant EOS. Furthermore, we found an enrichment of less rare *LRRFIP1* variants in individuals with idiopathic scoliosis within a large population sample. We reproduced this scoliotic phenotype in a KO mouse model.

## Results

### Phenotypic characteristics of LRRFIP1 EOS.

The index patient (patient III-12) presented to the medical genetics clinic because of severe, early-onset kyphoscoliosis (Figure 1, A–J) and a remarkable family history of EOS with affected individuals in 4 consecutive generations (Figure 2A) Vertical transmission of EOS through 4 generations, male-to-male transmission in several instances, and the presence of affected females are fully consistent with a monogenic cause of EOS with an autosomal-dominant mode of inheritance. The absence of healthy transmitters suggests complete penetrance of the phenotype. Affected family members had a scoliotic and/or kyphotic deformity that became apparent in the first years of life and progressed throughout childhood. While some affected individuals had a severe deformity that required surgical correction due to functional limitations, other affected individuals had a less extreme spinal deformity on physical examination (Figure 1, K and L) yet still showed the same severe, characteristic radiological pattern and pronounced spinal stiffness (Figure 1M). Besides EOS, none of the affected family members suffered from generalized musculoskeletal, neurologic, rheumatologic, or autoimmune diseases. No increased rates of bone fractures, hypermobility, muscle weakness, or exercise intolerance that would indicate a secondary etiology of the scoliosis were evident.

Radiologic imaging of affected family members (Figure 1) showed a distinctive pattern of pathologies. In the first years of life, a rounded deconfiguration of the vertebral bodies and decreased intervertebral spaces are evident. During childhood, rapidly progressive erosive changes of the vertebral endplates develop, most severely affecting the upper lumbar spine, progressing to ankylosis and complete obliteration of intervertebral spaces. The erosive endplate lesions gradually extend over time in a caudocranial direction to the thoracic spine and eventually to the lower cervical spine, leading to progressive spinal rigidity and scoliosis with or without kyphosis. The facet joints, spinous processes, and sacroiliac joints appear unaffected. Extraspinal skeletal elements show no dysplastic, erosive, or inflammatory aspects.

### Identification of an EOS-linked locus.

Both runs of genome-wide parametric linkage analysis showed a single peak mapping to chromosome 2q35q37 (Figure 2B). As the highest possible logarithm of the odds (LOD) score for the pedigree analyzed was Z(θ) = 2.61, the LOD scores of 2.4020 and 2.3971 at the 2q35q37 locus provided strong evidence for linkage. Fine mapping based on exome-sequencing data narrowed down the linked region to 470 kb on chromosome 2q37.3 encompassing 6 protein-coding genes (Figure 2C). To our knowledge, none of the 6 protein-coding genes within this region were known causes of isolated EOS (details are provided in the Supplemental Methods; supplemental material available online with this article; https://doi.org/10.1172/JCI202751DS1). RNA-seq did not show significant changes in mRNA expression levels of any of the 6 linked genes in cultured fibroblasts of EOS-affected individuals compared with age-matched unaffected relatives (Figure 2D).

### Identification and characterization of a noncoding candidate variant in LRRFIP1.

Exome sequencing did not show any rare candidate variants within or outside of the linked region. Genome sequencing in the affected individuals III-3 and III-12 revealed the rare noncoding heterozygous variant NM_001137550.2(*LRRFIP1*):c.96+34737C>T as the only potentially disease-causing variant. Sanger sequencing confirmed the presence of this variant in all 6 further tested affected patients and its absence in 4 unaffected family members. The variant is absent from healthy population control datasets (GnomAD, version 4.1.0) (10). This rare noncoding variant is located in the first intron of Matched Annotation from NCBI and EMBL-EBI (MANE) select transcript ENST00000308482.14 corresponding to the protein-coding isoform 1 of *LRRFIP1* (NM_001137550) and transcript ENST00000863709.1 corresponding to protein-coding isoform 2 of *LRRFIP1* (11). According to several sources including ENCODE (Z-Lab), Ensembl regulatory build, RefSeq, dbSUPER, and the Craniofacial Atlas, the NM_001137550.2(*LRRFIP1*):c.96+34737C>T variant is located within a high confidence enhancer element (GH02J237660) (12), which is predicted to interact with the nearby transcription start site (TSS) of *LRRFIP1* isoforms 1 and 2 and the downstream gene *RBM44* (Figure 3A).

The motifbreakR tool (13) predicted the variant to create a strong binding motif for the transcription factor nuclear receptor subfamily 3 group C member 1 (NR3C1) (Figure 3, B and C). The nuclear receptor subfamily 3 group C member 1 gene (*NR3C1)* encodes the human glucocorticoid receptor, a ubiquitously expressed hormone-activated nuclear receptor that works as a ligand-dependent transcription factor by binding to a specific glucocorticoid response element (GRE) of target genes (14). The GRE motif consists of 2 inverted hexameric sequences (GRE half-sites), separated by a 3 bp spacer (Figure 3B). While usually 2 NR3C1 molecules bind as dimers to such a GRE motif in a head-to-head orientation, NR3C1 monomers can also bind to GRE half-sites in conjunction with other nonglucocorticoid transcription factors (14). Although an electrophoretic mobility shift assay (EMSA) did not show increased binding affinity of isolated affinity-purified NR3C1 to *LRRFIP1* DNA duplex or single-stranded probes (Supplemental Figure 1; supplemental material available online with this article; https://doi.org/10.1172/JCI202751DS1), incubation with a mix of nuclear proteins including NR3C1 from retinal pigment epithelial 1 (RPE-1) cells or fibroblasts produced a shift for the single-stranded DNA probes that was consistently stronger for the mutant oligonucleotide containing the patient variant compared with the WT sequence (Figure 3D), showing that the binding affinity of 1 or multiple proteins to this DNA sequence was increased by the *LRRFIP1* c.96+34737C>T variant.

### LRRFIP1 c.96+34737C>T results in isoform expression changes in the presence of NR3C1.

RNA-seq did not detect statistically significant overall expression changes of *LRRFIP1* or any of the neighboring genes within the linked interval in cultured fibroblasts of affected patients (Figure 2D). An initial reverse transcription PCR (RT-PCR) analysis of *LRRFIP1* isoforms using published (15) primers indicated the presence of isoforms 1, 3, and 5 and NM_ 001137551 in patient and control fibroblasts (Supplemental Figure 5). For a comprehensive and quantitative analysis of isoform expression, we performed short-read RNA-seq, which showed expression of mainly isoforms 2, 4, 5, and 6 in all individuals (Figure 4A) and a shift in relative *LRRFIP1* isoform expression in fibroblasts of affected individuals with decreased expression of isoform 4 (–2.2 percentage points [pp] in generation II, –4.3 pp in generation III) and 5 (–8.5 pp in generation II, –4.3 pp in generation III), as well as increased expression of isoform 2 (+4.9 pp in generation II, +1.9 pp in generation III) (Figure 4, A–C). These compositional changes in isoform expression were recapitulated in an Aitchison principal component analysis (PCA), showing clear separation between WT and affected individuals and an Aitchison distance of 0.36 between group centroids (Figure 4B). To confirm these alterations in isoform expression, we performed long-read RNA-seq, which allows more accurate identification of isoforms by direct sequencing of full-length transcripts without the need for transcript assembly. Long-read PacBio IsoSeq data (439-682 FLNC reads per individual) confirmed the isoform expression differences observed in short-read data and indicated that the decrease of isoforms 4 and 5 was mainly driven by isoform 5 in the younger generation and of isoform 4 in the older generation. (Figure 4, D–F). Aitchison PCA of long-read compositions showed a larger Aitchison distance of 0.46, indicating an even clearer separation of isoform composition between affected and unaffected individuals (PC1: 66.9% variance; Figure 4E). Pairwise comparison of isoform proportions between affected and WT individuals within each generation in both short- and long-read datasets revealed a consistent pattern of reciprocal shifts in isoform expression in affected individuals with consistently decreased expression of isoforms 4 and 5 and increased expression of isoform 2 (Figure 4, C and F, and Supplemental Table 4). In line with these findings, Western blot analysis with an antibody that detected isoforms 3–5 showed decreased levels in affected individuals (Supplemental Figure 6).

RNA-seq of induced pluripotent stem cells (iPSCs) showed no relevant changes in isoform expression in *LRRFIP1* c.96+34737C>T heterozygous versus WT iPSC clones (Supplemental Figure 10A), and Aitchison PCA showed no separation between WT and heterozygous clones (Aitchison distance = 0.08; PC1: 68.7% variance; Supplemental Figure 10B). However, *NR3C1* expression was almost absent in iPSCs (mean transcripts per million [TPM], 2.0) and substantially lower compared with fibroblasts (mean TPM, 75.6; Supplemental Figure 11).

### Transcriptomic changes in patient fibroblasts indicate dysregulation of canonical Wnt signaling, PCP signaling, and inflammatory signaling.

Assessment of differentially expressed genes (DEGs) identified through RNA-seq of cultured fibroblasts showed 42 significantly upregulated and 39 significantly downregulated genes in patients with the *LRRFIP1* c.96+34737C>T variant in both generations (Supplemental Figure 9, Supplemental Table 10). PANTHER overrepresentation analysis (16) showed significant overrepresentation of extracellular matrix organization pathways, the Wnt signaling pathway involved in somitogenesis, and PCP regulation (Supplemental Tables 6 and 7). Significant DEGs included secreted frizzled–related protein 1 (*SFRP1*) and secreted frizzled–related protein 2 (*SFRP2*), which encode potent regulators of canonical Wnt signaling and the PCP pathway and receptor tyrosine kinase-like orphan receptor 2 (*ROR2*), which encodes a receptor able to bind Wnt ligands within the PCP pathway (17, 18). Significant DEGs involved in inflammatory signaling include coiled-coil domain containing 3 (*CCDC3*), which encodes a negative regulator of the TNF-α–induced proinflammatory response in endothelial cells via inhibition of TNF-α–induced NF-κB activation (19); baculoviral IAP repeat containing 3 (*BIRC3*), which encodes a modulator of inflammatory responses and regulates both canonical and noncanonical NF-κB signaling and has recently been identified as a key player in the pathogenesis of rheumatoid arthritis (20); and matrix metallopeptidase 3 (*MMP3*), which encodes a matrix-degrading enzyme able to cleave and remodel extracellular matrix proteins. *MMP3* is known for its role in the pathogenesis of ankylosing spondylitis, orchestrating tissue degradation influenced by TNF-α and NF-κB signaling (21) (Figure 4, G and H). A full list of DEG analysis results in fibroblasts and iPSCs is provided in Supplemental Table 10.

### Enrichment of rare LRRFIP1 variants in individuals with scoliosis within the UK Biobank cohort.

Our analysis of 484,903 genomes from the UK Biobank showed a significant enrichment of rare variants in the UTR and coding region of *LRRFIP1* in individuals with scoliosis. The ADD burden analysis showed a burden signal of risk-increasing near-private variants (minor allele count [MAC] ≤5) in the coding sequence + UTR mask (OR 2.66, *P* = 0.016) and the UTR + promoter mask (OR 2.45, *P* = 0.049) in *LRRFIP1* (unique region), while *COL6A3* intronic near-private variants were also significant but with a protective effect (OR 0.71, *P* = 0.019) (Figure 5). Individuals with scoliosis showed enrichment of regulatory UTR (62% vs. 48%) and loss-of-function (12% vs. 5%) variants in *LRRFIP1* compared with controls (Supplemental Figure 2), consistent with an enrichment of regulatory and loss-of-function variants driving the observed burden signal. Additionally, the analysis confirmed that the *LRRFIP1* c.96+34737C>T variant is exceedingly rare, with a minor allele frequency (MAF) of just 0.00031%.

### Idiopathic kyphoscoliosis in Lrrfip1-KO mice.

While some phenotyping screens were applied by the International Mouse Phenotyping Consortium (IMPC) (22), the *Lrrfip1*-KO mice had not been evaluated for kyphoscoliosis by the IMPC. Our analysis of the available radiographs of heterozygous and homozygous young adult *Lrrfip1*-KO mice revealed that *Lrrfip1*-KO mice had significantly lower kyphosis indexes, corresponding to more severe kyphosis compared with WT controls (*P* = 0.000913) (Figure 6, A and B) and a significantly higher prevalence of scoliosis (*P* = 0.0151) and larger scoliotic Cobb angles (*P* = 0.000651) compared with WT controls (Figure 6, C and D). Sixteen of 18 KO mice (88 %) had cervical hyperlordosis, which was not detected in any of the 46 WT mice. Phenotypic screens applied by the International Knockout Mouse Consortium (IKMC) showed no neuromuscular deficits or vertebral malformations, consistent with idiopathic scoliosis in *Lrrfip1*-KO mice. An in situ whole-mount *lacZ* reporter assay provided by the IMPC (22) showed expression of *Lrrfip1* in bone tissue of juvenile mice. Detailed measurements of kyphoscoliosis assessment and statistical analyses are provided in the Supplemental Methods, Supplemental Tables 8 and 9, and Supplemental Figure 3.

## Discussion

We identified a distinct type of autosomal-dominant EOS caused by a rare noncoding variant in the gene *LRRFIP1*, whose role in the disease is supported by a *Lrrfip1-*KO mouse model. Analysis of large-scale genome sequencing data from the UK Biobank further supports the involvement of rare variants in *LRRFIP1* in the etiology of scoliosis.

*LRRFIP1* EOS presents with a unique combination of spondylodysplastic elements with ovoid vertebral bodies, progressive multisegmental erosions, and ankylosis of vertebral endplates with onset in early childhood implicating both disturbed embryonic spine development and postnatal erosive processes in the pathogenesis. To our knowledge, this combination of phenotypic features does not correspond to any known disease, although some aspects overlap with known conditions. Ovoid vertebral bodies can be a feature of lysosomal storage disorders and some types of spondylometaphyseal dysplasia including *FN1*-related spondylometaphyseal dysplasia corner fracture type (21). Intervertebral disc space narrowing, progressive (anterior) endplate erosions, and kyphoscoliosis with spinal stiffness are features of progressive pseudorheumatoid dysplasia (23). However, compared with these entities, patients with *LRRFIP1* EOS lack dysplastic aspects of the extraspinal skeletal elements, and the erosive changes are exclusively limited to the vertebral endplates. Furthermore, these patients do not have any extraskeletal manifestations or systemic symptoms and are of normal stature.

Intervertebral disc space narrowing, endplate irregularities, and progressive kyphosis are seen in Scheuermann’s kyphosis (24). The earlier age of onset, the ovoid-shaped vertebrae, the multisegmental endplate erosions predominantly of the lumbar spine, and ankylosis differentiate *LRRFIP1* EOS from Scheuermann’s kyphosis. Spinal ankylosis is a key feature of ankylosing spondylitis (25), a form of spondyloarthritis with inflammation and progressive ankylosis of the sacroiliac, intervertebral, and facet joints associated with HLA-B*27 (25). Intriguingly, *LRRFIP1* was found to be significantly overexpressed in the blood of patients with ankylosing spondylitis (26), and patients with *LRRFIP1* EOS have increased expression of *MMP3*, a known finding in patients with ankylosing spondylitis (27). However, other than spinal ankylosis, patients with *LRRFIP1* EOS show no clinical signs of sacroilitis, inflammation of facet joints, enthesitis, dactylitis, or any other characteristic ankylosing spondylitis–related manifestations (25), and MRI images do not show the Romanus lesions or syndesmophytes typically seen in ankylosing spondylitis (28).

On the basis of these considerations, we conclude that, to our knowledge, *LRRFIP1* EOS is a previously undescribed disorder of the spine, which we propose to refer to as spondylodysplastic-erosive EOS.

Whether the individuals with scoliosis within the UK Biobank cohort who carry rare variants in *LRRFIP1* display spondylodysplastic-erosive EOS or unspecified types of scoliosis remains unknown, as most cases of scoliosis within the UK Biobank are coded as “unspecified” or “self-reported.” Furthermore, an underascertainment of scoliosis within the UK Biobank is likely, as the prevalence of reported scoliosis in this cohort corresponds to 0.76%, which is substantially lower than the generally accepted prevalence rates of scoliosis within the population (1).

The c.96+34737C>T variant observed in the *LRRFIP1* EOS family creates a new binding site for the transcription factor NR3C1 in an enhancer element for which interactions with the TSS of *LRRFIP1* isoforms 1 and 2 is predicted. Since we found that the protein mix present in nuclear extracts that includes NR3C1, but not isolated NR3C1, increases binding to the motif created by the *LRRFIP1* c.96+34737C>T variant, we presume that additional transcription factors or coactivator complexes must be present for NR3C1 to bind to the mutated sequence, as has been described for NR3C1-related transcriptional regulation This variant-induced ectopic NR3C1 binding most likely causes aberrant activity of the enhancer element, resulting in an increase in the expression of *LRRFIP1* isoform 2 but a decrease in the expression of isoforms 4 and 5. Accordingly, in isogenic iPSC lines with intrinsically absent *NR3C1* expression, we found no differences in *LRRFIP1* isoform expression with and without the c.96+34737C>T variant. An NR3C1-dependent effect of the c.96+34737C>T variant was also observed in patients’ fibroblasts: the older affected individual II-8, who was found to have higher expression of *NR3C1*, showed more pronounced changes in *LRRFIP1* isoform expression than did the younger affected individual III-12, who had lower *NR3C1* expression levels (Supplemental Table 2). These findings suggest that the pathogenic effects of the c.96+34737C>T variant are only exerted in the presence of NR3C1 and that this effect may be dosage dependent. This observation is particularly important, as it raises the possibility of potential therapeutic intervention in patients with *LRRFIP1* EOS using selective NR3C1 inhibitors, which are currently being developed for several other indications including Cushing’s syndrome (ClinicalTrials.gov NCT03697109).

With regard to the disease mechanism introduced by the c.96+34737C>T variant, a simple loss-of-function or gain-of-function model does not appropriately capture the intricate effects of this regulatory variant. While loss-of-function of *LRRFIP1* certainly plays a role in the pathogenesis of *LRRFIP1* EOS, as is evident from the *Lrrfip1*-KO mouse model and the enrichment of rare loss-of-function variants in *LRRFIP1* in individuals with scoliosis in the UK Biobank cohort, we propose that the c.96+34737C>T variant potentially involves both loss- and gain-of-function mechanisms, as shown by the relative decrease in expression of isoforms 4 and 5 and the relative increase in expression of isoform 2. This may explain the severe and highly penetrant phenotype observed in the family we describe here.

Although isoform 2 has high sequence homology with isoform 1, which has been described as a regulator of canonical Wnt signaling (15), less is known about the specific functions of isoforms 4 and 5, but the significant transcriptomic changes in patient fibroblasts implicate dysregulated canonical Wnt signaling, PCP signaling, and inflammatory signaling pathways in the pathomechanism underlying the scoliosis phenotype in patients with *LRRFIP1* EOS. These transcriptomic changes were absent in iPSCs that lacked *NR3C1* expression and hence showed no alteration in *LRRFIP1* isoform expression, suggesting that the transcriptomic changes in patients’ fibroblasts are downstream consequences of the c.96+34737C>T variant–induced alteration of *LRRFIP1* isoform expression. A loss-of-function effect is also supported by the enrichment of regulatory and loss-of-function variants in *LRRFIP1* in individuals with scoliosis in the UK Biobank cohort. *LRRFIP1* encodes a transcriptional regulator with diverse roles in key signaling pathways including canonical and noncanonical Wnt signaling, PCP signaling, and TLR signaling (29). Both Wnt signaling and PCP signaling (30) have previously been implicated in the etiology of EOS, and compromised Wnt signaling has been found to underlie progressive scoliosis with endplate deterioration (31). *LRRFIP1*, to our knowledge, has not been characterized as a monogenic disease gene but has been linked to wound healing, autoimmune processes, inflammation, obesity, thrombosis formation, excitotoxicity after stroke, and cancer (29). Rare variants in *LRRFIP1* were found in a cohort of patients with juvenile idiopathic arthritis (32), and collated analysis of GWAS data (33) revealed associations with juvenile rheumatoid arthritis, bone inflammation, and connective tissue and musculoskeletal diseases (26). *LRRFIP1* has been found to be highly expressed in nucleus pulposus cells of intervertebral discs (34), proliferating chondrocytes (35), and in the somites of mice (15) and zebrafish (36). The *LRRFIP1* isoform 1 ortholog is predominantly expressed in somites of embryonic mice (15), and this isoform is a critical enhancer of canonical Wnt signaling, a key signaling pathway during somitogenesis (7). The patient variant c.96+34737C>T is located in an enhancer in the first intron of isoforms 1 and 2, but isoform 1 is not expressed in human fibroblasts. Consistent with the role of *LRRFIP1* in Wnt signaling, we detected reduced RNA expression of *SFRP1* and greatly increased expression of *SFRP2* in patients’ fibroblasts. *SFRP1* and *SFRP2* are potent regulators of the Wnt signaling pathway during somitogenesis (17). Our findings are in line with a previous report of significantly increased expression of *SFRP2* induced by knockdown of *LRRFIP1* in murine myocytes (18), further supporting the idea of a loss-of-function effect induced by the c.96+34737C>T variant.

Furthermore, *LRRFIP1* is known to activate type 1 IFN and proinflammatory cytokine responses by regulating the TNF-α and NF-κB signaling pathways (29), which have been shown to be involved in degeneration of intervertebral discs (37) and vertebral endplates (38) and in the pathogenesis of ankylosing spondylitis (27) and is reflected by the altered expression of *BIRC3*, *CCDC3*, and *MMP3*, we detected in patients’ fibroblasts. Wounds treated with recombinant *LRRFIP1* show improved healing, decreased TLR4-mediated inflammation, and improved collagen deposition (39), demonstrating the effect *LRRFIP1* signaling has on extracellular matrix organization and inflammation, which was reflected by the significant (FDR 4.82E-04) enrichment of DEGs involved in extracellular matrix organization pathways we detected in fibroblasts from patients with *LRRFIP1* EOS.

On the basis of these findings, we propose that the *LRRFIP1* c.96+34737C>T variant contributes to the development of EOS by dysregulating PCP and Wnt signaling during somitogenesis, accounting for the developmental dysplastic aspects of *LRRFIP1* EOS, and by dysregulating TNF-α/NF-κB signaling, leading to disrupted extracellular matrix organization in an inflammation-type reaction accounting for the postnatal progressive endplate erosion and ankylosis.

We conclude that the c.96+34737C>T variant exerts a very specific effect on isoform expression in the presence of NR3C1, which was found to be expressed in early embryonic costal cartilage and vertebral tissue including bone and growth plates of embryonic mice (40).

The identification of an extremely rare variant in *LRRFIP1* as the cause of a fully penetrant, severe autosomal-dominant form of EOS, along with enrichment of less-rare variants of *LRRFIP1* in individuals with scoliosis within a large population sample is consistent with recent research showing that rare genetic variants causing monogenic diseases converge on shared genes and molecular signaling pathways with common disorders and complex traits (9, 41). This was also shown by the report of rare loss-of-function variants in the gene *PTK7* causing congenital scoliosis, while hypomorphic variants in the same gene were identified in individuals with adolescent idiopathic scoliosis (6). Likewise, we assume converging pathomechanisms for *LRRFIP1* EOS, Scheuermann’s kyphosis, and ankylosing spondylitis, given the phenotypic overlap and significant overexpression of *LRRFIP1* in the blood of patients with ankylosing spondylitis (26). This work substantially expands our knowledge about the etiology of EOS and enables the inclusion of *LRRFIP1* in genetic testing panels for EOS worldwide, thereby improving diagnostic yields and further advancing the characterization of this distinct disease — both prerequisites for future improvements in disease management and targeted therapies. Additionally, we provide insights into possible molecular mechanisms underlying more common phenotypes including ankylosing spondylitis and idiopathic scoliosis, whose etiopathology is still largely unsolved (24, 26). The NR3C1-dependent pathogenic effect of the *LRRFIP1* c.96+34737C>T variant is a promising target for future therapeutic approaches with selective NR3C1 inhibitors.

## Methods

### Sex as a biological variable.

Both the family-based analyses and the analysis of the UK Biobank cohort included female and male individuals. Our study of an *Lrrfip1*-KO mouse included female and male animals. Since *LRRFIP1*-EOS is inherited in an autosomal-dominant manner, the findings of this study are expected to be equally relevant for both sexes.

Additional details on the methods can be found in Supplemental Methods.

### Recruitment and sampling.

A total of 17 family members of the index patient III-12 were recruited for this study, including 10 affected and 7 unaffected individuals (Figure 2A). Anamnestic information was available for another 17 family members. A detailed clinical assessment of the core family was performed by clinical examination and review of medical reports and radiological imaging over a span of several years. The phenotype of members of the extended family was determined by interviews and review of radiographs and photographs. Peripheral blood samples were collected from the members of the core family. Saliva samples were sent in by all other participating individuals. Fibroblast samples were collected by skin punch biopsy from 4 individuals (Figure 2A).

### Parametric linkage analysis.

Parametric linkage analysis using the easyLINKAGE-Plus (42) package with Allegro (43) under assumption of autosomal-dominant inheritance was performed with genotyping data from Affymetrix CytoScan HD Micro-Arrays (now Thermo Fisher Scientific) covering 750,000 SNPs. SNP data retrieved as CYCHP files were converted into CYCHP.txt files using the executable apt-chp-to-txt from the Affymetrix Power Tools. Given the limited capacity of the package, SNP data on only 10 family members were used for the linkage analysis (Figure 2A), once with 100 markers and once with 83 markers per set with 1 marker every 0.5 cM. The calculated highest possible LOD score Z(θ) for this pedigree with 12 meioses separating the 10 genotyped individuals was 2.61, approximated by Z(θ) = log_10_[(1–θ)^12^/0.5^12^] (44). The chromosomal region that yielded the highest LOD score in both analyses was further visualized by generating haploplots with the output files of easyLINKAGE-Plus (42) using HaploPainter (Supplemental Figure 8) (45).

### Fine-mapping and linkage analysis by exome sequencing.

Using DNA extracted from peripheral blood lymphocytes or saliva samples from 11 individuals (*n* = 6 affected and *n* = 5 unaffected), whole-exome sequencing was performed using the IDT xGen Exome Research Panel (version 2) with paired-end sequencing on an Illumina NovaSeq 6000. The raw FASTQ files were aligned to the hg19 reference genome using the Illumina DRAGEN platform. Eleven VCF files were created with the Illumina DRAGEN platform. For quality control, several filter criteria were applied (DRAGEN quality score >30, mapping quality [MQ] >100, approximate read depth [DP] >25). The 11 VCF files were merged into a single VCF file using bcftools, reformatting with PLINK, and annotating with wANNOVAR in a Linux Subsystem for Windows (46–48). To validate the results from the classical parametric linkage analysis, we assessed the exome data for haplotype “blocks” over all chromosomes. A variant was considered a haplotype “hit” for our purposes when all affected individuals shared the same genotype and that genotype differed from all unaffected individuals. For a more straightforward and detailed visualization, the data from chromosome 2 were converted to an Excel worksheet, with genotypes and LOD scores incorporated. Since LOD scores correspond to markers that are 0.5 cM apart, most individual variants were located between markers, hence, we annotated the LOD scores as intervals. This allowed for manual evaluation of the linked region as described previously (44).

### Mutation screening by genome sequencing.

Using the Illumina TruSeq PCR-Free workflow and a NovaSeq 6000 platform as previously described (49), we performed whole-genome sequencing of 2 affected family members (III-3 and III-12) to screen the linked region for rare candidate variants. Only ultra-rare variants fully segregating with EOS were considered. Sanger sequencing of the candidate variant was performed for 10 additional family members, 6 affected and 4 unaffected, to confirm segregation of the candidate variant with the phenotype using standard protocols.

### Fibroblast culturing and RNA isolation.

Primary fibroblasts were cultured from mechanically fragmented skin-punch biopsy specimens from individual II-8 and his wife (II-8.5) and from individuals III-12 and III-13 and were subsequently cultivated and propagated in Advanced DMEM supplemented with 10% FBS, 5 mM GlutaMAX (Thermo Fisher Scientific) and penicillin-streptomycin. TrypLE Express Enzyme (Thermo Fisher Scientific) was used to detach cells for passaging. For RNA isolation, cells were seeded onto cell culture–treated 6-well plates at approximately 40% confluence. When cells reached confluence after 2 days, a full medium change was performed. After 24 hours, the culture medium was removed and cells lysed by addition of 1 mL/well TRIzol reagent (Thermo Fisher Scientific). RNA was extracted via chloroform-induced phase separation, followed by precipitation from the aqueous phase using isopropanol according to the manufacturer’s protocol. RNA pellets were resuspended in nuclease-free water and treated with DNase I (New England Biolabs) for 10 minutes at 37°C. The enzyme and buffer were removed by column purification using the Monarch Spin RNA Clean Up kit (New England Biolabs).

### Short-read RNA-seq and differential gene expression analysis.

RNA samples from 3 replicates (prepared from 3 subsequent passages) were processed for sequencing at the Functional Genomics Center Zurich (FGCZ). Sequencing libraries were prepared from 300 ng total RNA using the TruSeq Stranded Total RNA Kit (Illumina). Paired-end sequencing (150 bP) was done on a NovaSeq X system (Illumina). An average of approximately 150 million reads were generated per sample. For analysis of DEGs, the sequencing data were processed with the data analysis framework Support Users for Shell script Integration (SUSHI) (https://fgcz-sushi.uzh.ch/) on the University of Zurich server. Alignment to genome assembly GRCh38.p13 was done using the application STAR with sequence trimming of the first 10 base pairs, adapters, and low-quality tails. Differential gene expression was assessed using a negative binomial generalized linear model (GLM) with a quasi-likelihood *F* test implemented in edgeR (50). *P* values were adjusted for multiple testing using the FDR, with FDR values below 0.05 considered statistically significant. DEG analysis of fibroblasts showed significant age-dependent effects with age-related clustering (Supplemental Figure 8A). For this reason, the DEG analysis was performed for both generations separately (Supplemental Figure 9B). Only DEGs that were significantly differentially expressed in both generations were input for the functional enrichment analyses.

### Functional enrichment analysis.

With the list of DEGs, a PANTHER overrepresentation test (51) was run using the Gene Ontology (GO) Biological Process Complete dataset (52) and the Reactome pathways dataset (Reactome version 86, released September 7, 2023).

### Short-read RNA-seq isoform quantification.

Transcript-level isoform quantification was performed using Salmon version 1.10.3 (53) in mapping-based mode with a GENCODE version 49 transcriptome index including decoy sequences. While GENCODE version 49 lists 145 transcripts for *LRRFIP1* (54), we considered the 5-well supported and validated protein coding isoforms and the 1 noncoding APPRIS P1 isoform for isoform expression analyses and annotated to 6 manually curated isoforms (isoforms 1–6; Figure 3A and Figure 4). For fibroblasts, 3 replicates per individual were quantified for 4 individuals: II-8.5 (healthy, WT), II-8 (affected, *LRRFIP1* c.96+34737C>T heterozygote), III-13 (healthy, WT), and III-12 (affected, *LRRFIP1* c.96+34737C>T heterozygote). For iPSCs, 3 WT clones (WT-1 to WT-3) and 3 isogenic heterozygous clones (Het-1 to Het-3, heterozygous for the *LRRFIP1* c.96+34737C>T variant) were quantified, each with up to 3 replicates. Two iPSC samples with a total *LRRFIP1* TPM below 10 were excluded as outliers (Het-2 and Het-3 retained 2 replicates each). Overall TPM values for *LRRFIP1* and the 5 neighboring genes within the linked region (*COL6A3*, *MLPH*, *PRLH*, *RAB17*, *RBM44*) were obtained by summing transcript-level TPM per gene using GENCODE version 49 transcript-to-gene mapping. TPM values are reported as the mean of individual or clone means, not as a grand mean across all samples.

### Long-read RNA-seq.

For long-read RNA-seq, sequencing libraries were generated at the Functional Genomics Center Zurich (FGCZ) from 300 ng total RNA from 1 experimental replicate of individuals II-8, II-8.5, III-12, and III-13 using the Kinnex full-length RNA kit (Pacific Biosciences of California). Samples were barcoded during cDNA synthesis and pooled after the cDNA amplification step. Sequencing was done on a Revio long-read system (Pacific Biosciences of California) at the Institute of Medical Genetics, University of Zurich. PacBio IsoSeq long-read data was processed through the IsoSeq analysis pipeline (PacBio SMRT Link software tools) to generate full-length nonchimeric (FLNC) reads.

### Long-read RNA-seq isoform quantification.

Transcript-level quantification was performed using IsoQuant version 3.4 (55) with GENCODE version 49 annotation as a reference. FLNC read counts were assigned to the 6 curated *LRRFIP1* isoforms as detailed above; reads mapping to other annotated or novel *LRRFIP1* transcripts were grouped as “other.” Isoform fractions were computed as the proportion of FLNC reads assigned to each isoform relative to the total *LRRFIP1* FLNC read count per individual. Gene-level full-length values for the linked genes were obtained from the IsoSeq classification pipeline (whole-transcriptome quantification), which provides proper cross-gene normalization across 21,074 genes.

### Isoform fraction and proportion comparisons.

Isoform fractions were computed for each sample as the ratio of isoform TPM to total *LRRFIP1* TPM and then averaged across replicates within each individual (for the short-read RNA-seq data from fibroblasts) or clone (for short-read RNA-seq data from iPSCs). Pairwise differences in isoform proportions between affected and healthy individuals were computed within each generation from the Salmon short-read RNA-seq data and from the IsoQuant long-read FLNC data, respectively. For generation II, the comparison was done between affected individual II-8 and unaffected individual II-8.5; for generation III, the comparison was done between affected individual III-12 and unaffected individual III-13. Differences are reported in pp (Δpp), computed as the isoform proportion in affected individuals/samples minus the isoform proportion in unaffected individuals/samples for each isoform.

### Aitchison PCA of isoform compositions.

To compare isoform composition profiles between individuals, an Aitchison PCA was applied, which is a PCA specifically designed for the analysis of compositional data. Isoform fractions were treated as compositions on the simplex. A pseudocount of 10^–6^ was added to zero fractions, compositions were reclosed, and a centered log ratio (CLR) transformation was applied. The PCA was performed on the CLR-transformed data. For fibroblasts (short-read and long-read), 4 isoforms with non-negligible expression (isoforms 2, 4, 5, and 6) were included in the PCA; for iPSCs, all 6 isoforms were included, as all were meaningfully expressed (≥8% mean fraction). The Aitchison distance between WT and affected (or heterozygous) group centroids was computed as the Euclidean distance between CLR-transformed centroid vectors.

### CRISPR/Cas9-mediated genome editing.

For genome editing to introduce the *LRRFIP1* patient variant [NM_001137550.2(LRRFIP1): c.96+34737C>T] into the WT iPSC line, the following reagents from Integrated DNA Technologies (IDT) were used: Alt-R S.p. Cas9 Nuclease V3; Alt-R CRISPR-Cas9 tracrRNA; Alt-R CRISPR-Cas9 crRNA (sequence: UCUAAGGGGUUAAGAGCACA); and Alt-R HDR donor oligonucleotide (sequence: AAGAGAAAGAAAAAAAATACATTCAGACATTGGACCTGAAAAAAAAATTGTCCTTATCTT TTCTAAGGGGTTAAGAACACATGGCACACACCACACCTTAGCACCCTGAAGAATAAAA). iPSCs were dissociated with Accutase solution (Merck KGaA), and 3 × 10^5^ cells were nucleofected with Cas9 gRNA ribonucleoprotein complex and a donor template using the P3 Primary Cell 4D-Nucleofector Kit and the 4D-Nucleofector System (Lonza) (electroporation program CM150). Forty-eight hours after nucleofection, iPSCs were seeded at single-cell density onto 10 cm culture dishes and later manually picked for clonal expansion. Genome-editing outcomes were assessed by Sanger sequencing.

### iPSC culture and RNA isolation.

iPSCs were cultured in StemFlex medium (Thermo Fisher Scientific) on cell culture–treated multi-well plates that were coated with Geltrex matrix (Thermo Fisher Scientific). To detach cells for passaging, 0.5 mM EDTA in PBS was used. For RNA isolation, 3 iPSC clones with heterozygosity for the *LRRFIP1* patient variant (NM_001137550.2(*LRRFIP1*): c.96+34737C>T) and 3 isogenic WT clones were seeded onto 6-well plates at a density of 150,000 cells per well in StemFlex culture medium with 10 μM Y-27632 (MedChemExpress). After 48 hours, the culture medium was exchanged with fresh StemFlex without Y-27632. Cell lysis was performed on day 4 after cell seeding by addition of 1 mL/well TRIzol reagent (Thermo Fisher Scientific). Samples from 2 independent experimental replicates were collected. RNA was extracted via chloroform-induced phase separation, followed by precipitation from the aqueous phase using isopropanol according to the manufacturer’s protocol. RNA pellets were resuspended in nuclease-free water and treated with DNase I (New England Biolabs) for 10 minutes at 37°C. The enzyme and buffer were removed by column purification using the Monarch Spin RNA Cleanup kit (New England Biolabs).

### motifbreakR.

To assess potential transcription factor–binding effects of the *LRRFIP1* variant identified in the EOS family, the R/Bioconductor package motifbreakR version 2.14.2 (13) was utilized with the HOCOMOCO version 10 (core) database. Reference and alternate alleles were compared using the information content algorithm (*P* ≤ 1 × 10^–4^). Predicted binding affinities were expressed as normalized motif scores ranging from 0–1. Motifs with a normalized binding score of 0.85 or higher in either allele were retained as high-confidence binding sites. Variants showing a difference in the normalized binding score between alleles (Δ ≠ 0) were classified as potentially disruptive, indicating a predicted alteration in transcription factor–binding strength between the reference and alternate sequences. Scores were normalized to the motif matrix (scoring range, 0–1). If either *F*(*s*_\textscref_, *M*) or *F*(*s*_\textscalt_, *M*) exceeded the threshold of 0.85 (corresponding to *P* ≤ 1 × 10^–4^), the motif was reported; neutral effects Δp_i<0.4 were not reported.

### EMSA.

To functionally test whether the NM_001137550.1(*LRRFIP1*):c.96+34737C>T variant alters the binding affinity of NR3C1, EMSA was performed using biotin-labeled DNA probes (39 nt) spanning the region around the patient variant incubated together with nuclear extracts from RPE-1 or fibroblasts, or eluate sample from NR3C1-FLAG IP. RPE-1 cells were selected for nuclear extracts because of the good expression levels of NR3C1 (nTPM 47.3 in HTERT-RPE-1 according to the GTEx Human Protein Atlas) and fibroblasts because of the planned further analyses of patients’ fibroblasts.

The following pairs of HPLC-purified DNA oligonucleotides with 5′-biotin labeling were ordered from IDT: *LRRFIP1* WT probe, 5′-TGTGGTGTGTGCCATGTGCTCTTAACCCCTTAGAAAAGA-3′ and 5′-TCTTTTCTAAGGGGTTAAGAGCACATGGCACACACCACA-3′; and *LRRFIP1*-mutant probe, 5′-TGTGGTGTGTGCCATGTGTTCTTAACC CCTTAGAAAAGA-3′ and 5′-TCTTTTCTAAGGGGTTAAGAACACATGGCACACACCACA-3′ (the underlined nucleotide indicates site of mutation). Corresponding oligonucleotides were annealed at 10 μM concentration in duplex buffer (IDT) to generate dsDNA probes. Nuclear extracts were prepared from cultured RPE-1 cells (American Type Culture Collection [ATCC]) or fibroblasts through sequential incubation with hypotonic and high-salt buffer. Briefly, cell pellets were resuspended in buffer A (10 mM HEPES, pH 7.9, 1.5 mM MgCl_2_, 10 mM KCl, 0.5 mM DTT and protease inhibitors) and incubated for 60 minutes on ice before lysates were passed 10 times through a 26 gauge needle using an attached 2 mL syringe. Following 10 minutes of centrifugation at 750*g* at 4°C, nuclear pellets were resuspended in approximately twice the volume of buffer B (20 mM HEPES, pH7.9, 1.5 mM MgCl_2_, 420 mM NaCl, 0.2 mM EDTA, 10% glycerol, 0.5 mM DTT and protease inhibitors). Samples were incubated for 15 minutes on ice and then centrifuged for 45 minutes at 20,000*g* at 4°C. Supernatants were mixed with equal volumes of buffer C (20 mM HEPES, pH 7.9,1.5 mM MgCl_2_, 0.2 mM EDTA, 30% glycerol, 0.5 mM DTT). For IP of NR3C1, an ORF plasmid for expression of human NR3C1 with C-terminal FLAG-Tag (RC220189, Origene) was transfected into HEK293 cells (DSMZ) using Lipofectamine 2000 transfection reagent (Thermo Fisher Scientific). Cells were lysed with IP lysis buffer (25 mM Tris-HCl, pH 7.4, 1 mM EDTA, 150 mM NaCl, 5% glycerol, 1% NP-40, HALT protease inhibitor cocktail). NR3C1-FLAG was immunoprecipitated from the lysate using mouse anti-FLAG antibody (clone M2, F1804, Merck) coupled to Protein G magnetic beads (MedChem Express). Elution was done by incubation with free 3× FLAG peptide (HY-P0319, MedChem Express) for 30–36 hours at 4°C using a 550× molar excess compared with coupled antibody.

EMSA binding reactions were performed with reagents from the LightShift Chemiluminescent EMSA kit (Thermo Fisher Scientific) according to the manufacturer′s protocol. Briefly, 20 μL binding reactions were set up with 1.5–3 μg nuclear extract or 2 μL IP eluate sample, and 2 μL of 10 nM dilutions of duplex DNA probe, 2 μL binding buffer, 1 μL of 50% glycerol, 1 μL of 100 mM MgCl2, 1 μL NP40, 1 μL of 1 μg/μL poly(dI.dC), and 1 μL of 1% NP-40. Reactions were incubated at room temperature for 50 minutes and then electrophoresed with active cooling through a 6% nondenaturing polyacrylamide gel in 0.5 × tris-borate-EDTA (TBE) buffer. Semidry transfer to a nylon membrane (0.45 μm pore size) was done in 0.5× TBE buffer for 8 minutes at 25 V. DNA probes were crosslinked to the membrane by 10 minutes of UV light exposure. Detection of biotin-labeled probe on the membrane was done either with the HRP-coupled Streptavidin and Luminol substrate from the LightShift Chemiluminescent EMSA kit (Thermo Fisher Scientific) and imaging on a ChemiDoc XRS+ system (Bio-Rad Laboratories), or by incubation of the membrane with IRDye 800CW–coupled Streptavidin (LI-COR Biotechnology) and imaging on an Odyssey DLx system (LI-COR Biotechnology).

### UK Biobank rare variant enrichment analysis.

For this project, approval to access large-scale genomic and health data was granted by UK Biobank (project ID 435853).

For the rare variant enrichment analysis, scoliosis cases and controls were ascertained from the UK Biobank 500k release (*n* = 484,903 participants with whole-genome sequencing data after standard sample quality control, including removal of withdrawn participants, sex mismatches, excess relatedness, and high heterozygosity). Gene-based enrichment testing was performed for all 6 genes within the linked region on chromosome 2q37 (*LRRFIP1*, *COL6A3*, *MLPH*, *PRLH*, *RAB17, RBM44*) in individuals with scoliosis (*n* = 3,668 cases) and without scoliosis (*n* = 481,235 controls). The phenotype was defined as a binary phenotype using the International Classification of Diseases–10 (ICD-10) code M41 (scoliosis, including kyphoscoliosis) from hospital inpatient records, primary care data, and self-reported conditions (UK Biobank field 20002). As most cases of scoliosis in the UK Biobank are coded as “unspecified” or “self-reported,” it was not possible to model a more defined subtype of scoliosis or for age of onset of scoliosis. Covariates included age at recruitment (field 21022), genetic sex (field 31), and the first 5 genetic principal components derived from genotype imputation (field 22009, components 1–5). Whole-genome sequencing data from the UK Biobank 500k release were accessed via the DNAnexus Research Analysis Platform (RAP). Variant calls were obtained from GraphTyper population-level variant call format (pVCF) files (field 23374). For the target region on chromosome 2 (chr2:237,324,003-237,842,808; GRCh38), 28 pVCF blocks (blocks 11865-11892) were downloaded and concatenated using bcftools concat (--naive mode). The merged VCF was converted to the PLINK binary format using PLINK 2.0, with variant identifiers encoded as chr:pos:ref:alt. Multiallelic sites were restricted to biallelic records (--max-alleles 2), and deletion placeholder alleles (*) were removed. The final cleaned dataset was verified to contain no duplicate variant identifiers. Variants within each gene were annotated into functional subregions using GENCODE version 49 (gencode.v49.annotation.gtf). Four mutually exclusive regions were defined for each gene: coding sequence (coding), 5′ and 3′ UTR, intronic region, and promoter region (defined as the 1 kb region upstream of the TSS). These subregions were combined into 6 annotation masks for burden testing (coding; UTR+coding; UTR + promoter; intronic; UTR + coding + intronic; all regions). Because of the partial overlap of the sequences of *LRRFIP1* and *RBM44*, the analysis was separated into the respective unique regions and the overlap region, defining a total of 7 gene sets. Variant-to-gene annotation files (.annot), gene set lists (.setlist), and mask definitions (.masks) were generated programmatically from the cleaned BIM file and GENCODE GTF using a custom Python script.

Gene-based rare variant enrichment testing was performed using REGENIE version 3 (56) in a 2-step framework for binary traits with Firth logistic regression and saddle-point approximation (--bt --firth --approx). For whole-genome regression, leave one chromosome out (LOCO) predictions were computed using common variants from UK Biobank array genotype data (field 22418, all 22 autosomes merged). Array variants were filtered for a MAF of greater than 1%, a MAC of greater than 100, a genotyping rate of greater than 90%, a Hardy-Weinberg equilibrium *P* > 1 × 10^–15^, and an individual call rate of greater than 90%. This was run with a block size of 1,000 SNPs and 16 threads. For the region-based testing, rare variant burden tests were conducted on the cleaned whole-genome sequencing (WGS) data for the 6 genes in the linked region. Each gene was tested across the 6 annotation masks and 3 allele frequency strata: primary (alternative allele frequency [AAF] ≤1%), ultra-rare (AAF ≤0.1%), and near-private (MAC ≤5). All frequency strata applied a minimum MAC threshold of 5 for variance component tests (--vc-MACthr 5) and a minimum MAC of 1 for inclusion (--minMAC 1).

We chose to use the additive count (ADD) burden test statistic, a gene-based additive burden test, because it supports the analysis of very rare variants (MAC ≤5) and additionally provides a directionality analysis to differentiate whether an enrichment of rare variants is risk increasing or protective toward the phenotype modeled. Burden test SNP lists were written for downstream inspection (--write-mask-snplist), and annotation files were validated with strict checking (--check-burden-files --strict-check-burden). All rare variants within the analyzed gene sets were functionally annotated using the Ensembl Variant Effect Predictor (VEP) version 115 (see Supplemental Methods, including Supplemental Figure 2) (57).

Analyses were performed on the DNAnexus RAP using the following: REGENIE version 3 (gene-based tests), PLINK 2.0 (VCF conversion, QC, allele frequencies), bcftools (VCF concatenation), GENCODE version 49 (functional annotation), and PLINK 1.9 (array merge). The forest plot was generated using matplotlib version 3.x in Python. Computation was carried out on a mem3_ssd1_v2_x16 instance (16 cores, 128 GB RAM).

### Mouse model.

The mouse model analyzed in this study was created through the IKMC Project (58). The Lrrfip1^tm1.1(KOMP)Wtsi^ reporter–tagged deletion allele was created by a targeted Cre-mediated excision, resulting in the removal of the neomycin selection cassette and critical exon(s) while leaving behind the inserted lacZ reporter sequence using an L1L1_Bact_P vector in C57BL/6N mice, resulting in a null allele (58). Since these mice were not analyzed for scoliosis by the IMPC, we evaluated radiographs available through their website. For our analysis of kyphoscoliosis in Lrrfip1^tm1.1(KOMP)Wtsi^ mice, we used the radiographs of 18 KO mice and 46 WT mice, since they were available on the IMPC website (https://mousephenotype.org) in March 2023. The individual IMPC specimen IDs for each evaluated mouse are listed in Supplemental Tables 4 and 5. To exclude as many confounding factors as possible, we analyzed only mice that were placed consistently and in accordance with the recommendations for standardized radiographic assessment of scoliosis in mice (59). Deviating from these recommendations, the tails of the mice were bent to the left, but this was consistently done in the same manner in all mice, and, as previously demonstrated, tilting of the tail does not result in any relevant deviation of the spinal alignment (59). Only mice aged 13 or 14 weeks (young adults) were included in the analysis. We analyzed whole-body anteroposterior and lateral conventional radiographs of 18 KO mice (*n* = 11 heterozygous, *n* = 7 homozygous) and 46 WT control mice. A Cobb angle of at least 10° was used as the cutoff for a positive scoliotic phenotype. We measured scoliotic Cobb angles using Microsoft Powerpoint by creating a virtual goniometer as described previously (60) (Supplemental Figure 3). To evaluate kyphotic deformities, we analyzed lateral whole-body conventional radiographs. Two WT mice had to be excluded for suboptimal placement of the hind legs. Otherwise, WT and KO mice had all been positioned in a consistent, comparable position. Because some degree of thoracic kyphosis is naturally present in mice, we needed to develop a specific kyphosis index to measure kyphotic changes that exceed the normal spinal curvature. In this effort, we were guided by the method proposed by Laws and Hoey (61). However, to account for the cervical hyperlordosis that was observed in many KO mice, we developed a modification of their kyphosis index generating an adapted kyphosis index (Supplemental Figure 3) to account for the added effect of cervical hyperlordosis on the total dorsoventral deviation, thereby allowing a reliable assessment of the extent of the kyphosis with lower kyphosis index values representing more severe kyphotic deformities (Supplemental Figure 3). In addition, we evaluated all mice for the presence or absence of cervical hyperlordosis (Supplemental Figure 3). Cobb angle and kyphotic index measurements were performed by an investigator blinded to the mutation status, age, and sex of the mice by assigning a randomly generated identification number to each radiograph and shuffling the order of the radiographs. We repeated the angle measurement for each radiograph after 7 days in a different random order and used the average of the 2 measurements for statistical analysis. Measurements are provided in Supplemental Tables 4 and 5. Since there were no statistically significant differences in Cobb angle values (*t* = 1.55388; *P* = 0.139768), kyphosis index values (*t* = 0.40561; *P* = 0.690401), or scoliosis prevalence (Fisher’s exact test *P* = 0.1448) between homozygous and heterozygous *Lrrfip1*-KO mice, we did not differentiate between heterozygous KO (*n* = 11) and homozygous KO mice (*n* = 7) for the comparison with the WT control sample.

### Statistics.

The UK Biobank gene-based rare variant enrichment testing was performed using REGENIE version 3 (56) in a 2-step framework for binary traits with Firth logistic regression and saddle-point approximation. Gene-level associations were evaluated using the additive (ADD) burden test. ORs and 95% CIs were calculated for each gene mask to assess the cumulative effect of rare variants across 7 gene sets and 6 variant masks (42 tests in total). Statistical significance was assessed at a nominal threshold of *P* < 0.05; after Bonferroni correction for 42 tests, the significance threshold was *P* < 1.19 × 10^–3^.

For the DEG-based overenrichment tests, a Fisher’s exact test with correction by calculation of the FDR by the Benjamini-Hochberg procedure was performed for each set. Only results with an FDR below 0.05 were considered.

To calculate the significance of the difference in prevalence of scoliosis and cervical hyperlordosis between WT and *Lrrfip1*-KO mice, a Fisher’s exact test with a 2 × 2 contingency was used. An independent 2-tailed *t* test was applied to compare the mean Cobb angles and the mean kyphotic indexes with the effect size measured by calculating hedges g. To determine whether the differences in Cobb angles and kyphotic indexes between KO and WT mice were significant, a 1-tailed Mann-Whitney *U* test was calculated. All statistic calculations were performed using vassarstats.net (62) and socscistatistics.com (63, 64). Significance was set at a *P* value of less than 0.05 for all calculations. Expression data in Figure 2D and Figure 4, G and H are presented as individual data points, mean ± SD.

### Study approval.

This project received approval from the ethics commission of the Canton of Zurich, Switzerland (BASEC-Nr. 2021-01631). This study was conducted according to the principles of the Declaration of Helsinki. For the family-based analyses, written informed consent for genetic testing and publication of clinical and genetic data was obtained from each individual prior to participation in the study. Written informed consent for the use of photographs and radiographs was also obtained. For the UK Biobank-based analyses, the UK Biobank received ethics approval from the North West Multi-centre Research ethics committee as a research tissue bank (RTB).

### Data availability.

Values for all data points in graphs and reported means presented in the main text and supplemental material are reported in the Supporting Data Values file. Genetic raw data on the family with monogenic EOS are not made publicly available because of consent restrictions and privacy protection. Part of the study using UK Biobank data was conducted using the UK Biobank Resource under application number 435853. Whole-genome sequencing data were accessed via the UK Biobank RAP (https://ukbiobank.dnanexus.com/landing). The UK Biobank data are accessible to researchers with an approved UK Biobank project (https://www.ukbiobank.ac.uk/use-our-data/eligibility/). All analyses were conducted on the UK Biobank RAP using DNAnexus applications, command-line tools, and R/Python scripts. Analytic code is available through the GitHub repository (https://github.com/carlalbc/LRRFIP1-analysis; commit ID a277ddf). Radiographs of the mouse model were retrieved from the IKMC and IMPC (www.mousephenotype.org), available under the Creative Commons Attribution 4.0 International license (CC-BY 4.0).

## Manuscript information

### Author contributions.

Clinical and imaging data were acquired by TF, AR, KS, and KM. TF, AR, KS, KM, and RS analyzed and interpreted clinical and imaging data. EMC, MT, CB, GS, AR, and TF performed bioinformatics and statistical analyses. GS, SvG, NR, and MZ conducted experiments. UK Biobank analyses and functional variant annotation were performed by CB and AR. TF performed the analysis of the radiographs of the mouse model. CB and GS performed short- and long-read RNA-seq, DEG analysis, and isoform expression analyses. II, PB, MP, MZ, EMC, and AR performed exome and genome sequencing and analyzed the genomic sequencing data. TF, AR, EMC, GS, CB, and RS were involved in the writing of the manuscript. TF acquired funding. AR conceived and supervised the study. All authors read and approved the manuscript.

### AI disclosure.

The authors declare that no AI tools were used in the original research for or writing of this article.

### Conflict of interest.

The authors have declared that no conflict of interest exists.

### Funding.

This work was supported by the following:

- UK Biobank Platform Credits Programme, Getting Started Credit (funded by Amazon Web Services; ukbgrant_2501, to TF).

## Supplementary Material

Supplemental data

Unedited blot and gel images

Supplemental table 10

Supporting data values

## Figures and Tables

**Figure 1:**
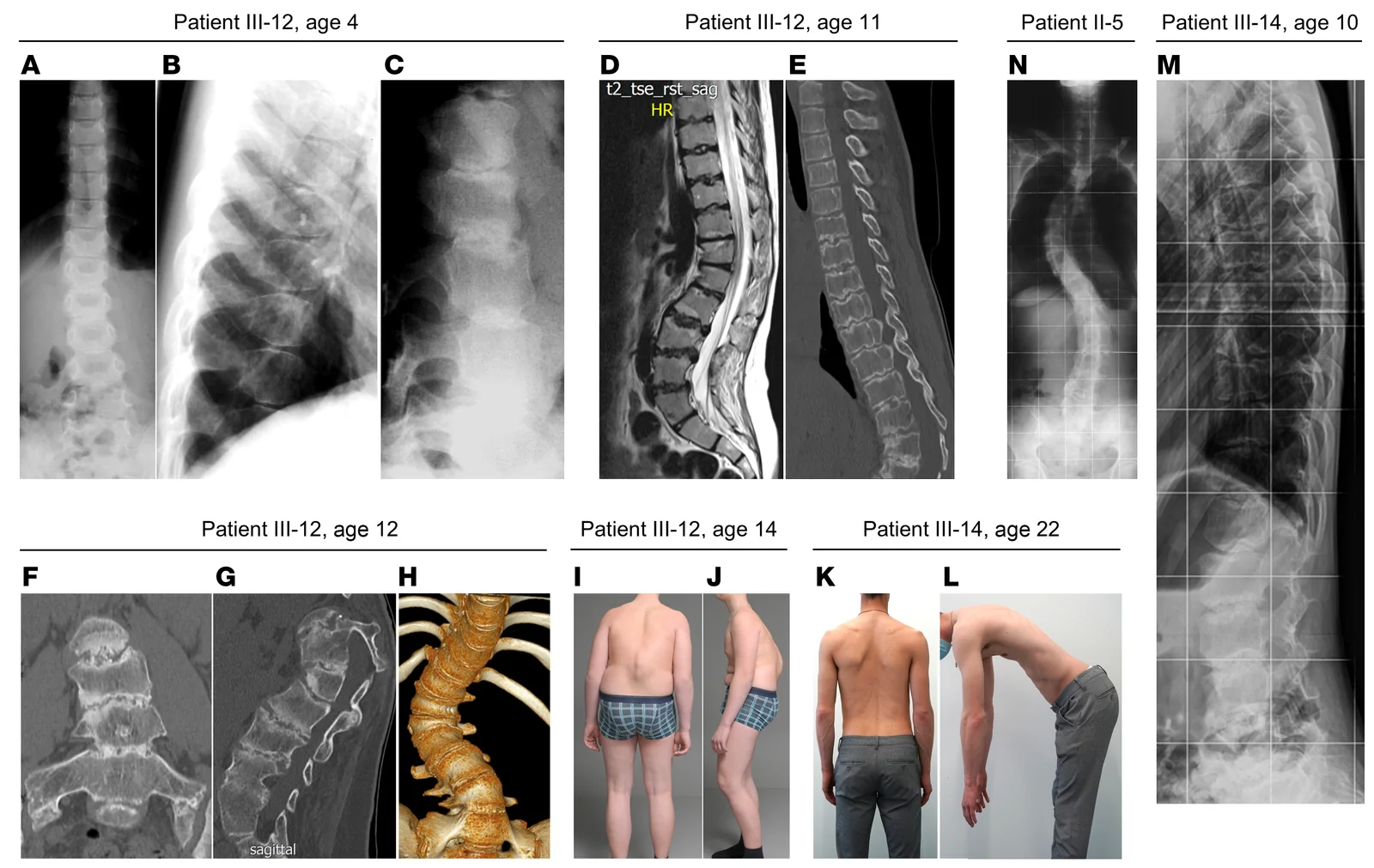
Phenotype of selected individuals with *LRRFIP1* EOS. (**A**–**J**) Image of individual III-12. Radiographs at age 4 show mild lumbar scoliosis (Cobb angle 8°) (**A**), loss of intervertebral spaces (**A**–**C**), ovoid deconfiguration of the thoracic vertebrae (**B**), and erosive endplate lesions in the lumbar segments (**C**). Images at age 11 (**D** and **E**) show thoracolumbar kyphosis with severely decreased intervertebral spaces and extensive endplate erosions. MRI shows T2-hyperintense reactive Modic changes, bulging intervertebral discs, and endplate irregularities (**D**). CT images show a gradual extension of endplate changes in a caudocranial direction, calcifications of intervertebral discs, coronal clefts, and irregularly shaped vertebral bodies (**E**). CT scans of the lumbar spine at age 12 (**F** and **G**) show complete obliteration of intervertebral spaces, severe erosion of the endplates, and multisegmental ankylosis. 3D-rendered CT at age 14 (**H**) shows severe thoracolumbar kyphoscoliosis. Photographs at age 14 (**I** and **J**) show a gibbus deformity at the thoracolumbar junction and severe kyphoscoliosis with a kyphotic Cobb angle of 110° (T10–L3) and a scoliotic Cobb angle of 50° (T12–L4), as well as resulting functional extension deficits of knee and hip joints. (**K**–**M**) Images of individual III-14. Photographs at age 22 show mild scoliosis with asymmetry of the scapulae (**K**) and severe spinal stiffness in maximum spinal flexion (**L**). Lateral radiograph at age 10 (**M**) shows loss of physiological spinal curvature, rounded deconfiguration of thoracic vertebrae, obliterated intervertebral spaces, multisegmental endplate irregularities, and ankyloses of the lumbar and lower thoracic vertebrae identical to those seen in patient III-12 and II-5. (**N**) Radiographic image of individual II-5 at age 51 shows thoracolumbar scoliosis (thoracic curve with a Cobb angle of 64°and lumbar curve with a Cobb angle of 53°), obliterated intervertebral spaces, and long stretches of ankylosis. Note that enlarged radiographic images of the images shown in **A**–**G**, **M** and **N** are provided in Supplemental Figure 7.

**Figure 2:**
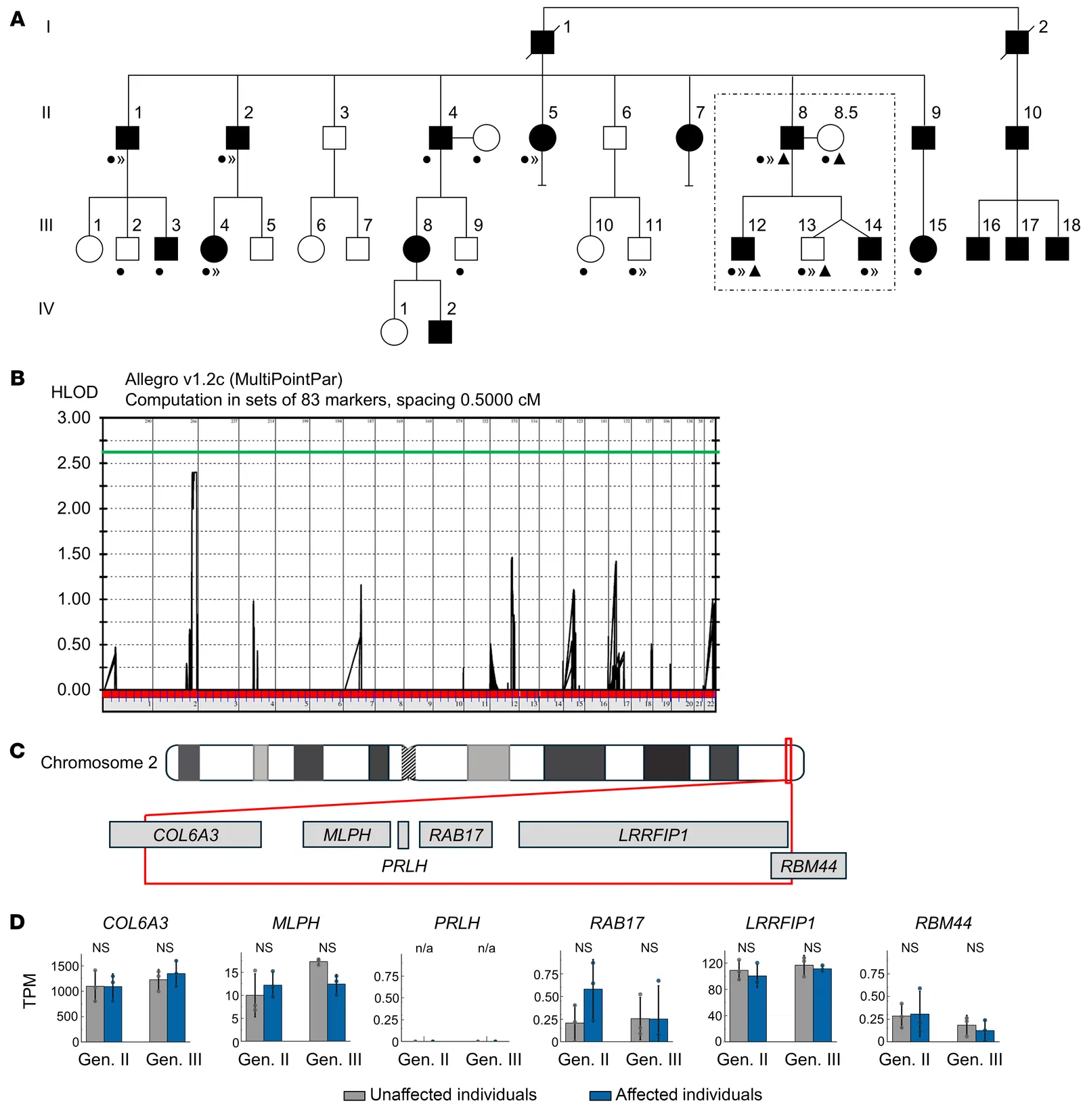
Family-based linkage analysis and investigations of the linked region. (**A**) Schematic showing the large nonconsanguineous pedigree with affected individuals in 4 generations. Individuals were considered affected by EOS if there was documentation of a scoliotic or kyphotic deformity of more than 10° with onset before the age of 10 years and/or the characteristic pattern of ovoid vertebrae, endplate erosion, and ankylosis. Affected individuals are represented by solid black symbols. The dotted square denotes the core family with index patient III-12. ●, DNA sample available; », linkage analysis; ▲, RNA-seq. (**B**) Heterogeneity logarithm of the odds (HLOD) scores for the genome-wide parametric multipoint linkage analysis with 83 markers per set. The highest LOD score of 2.4020 maps to an approximately 12.9 Mb locus on chromosome 2q36.3-2q37.3 (GRCh37/hg19 chr2:227482752-240350326). The green horizontal line represents the highest possible LOD score Z(θ) = 2.61 for the pedigree analyzed. (**C**) Linked locus after exome sequencing–based fine mapping spanning 470 kb on chromosome 2q37.3 (GRCh37/hg19 chr2:238263489-238732864) encompassing 6 protein-coding genes. (**D**) RNA-seq data from cultured fibroblasts (triplicates) of affected individual II-8 and unaffected individual II-8.5 (generation II) and affected individual III-12 and unaffected individual III-13 (generation III) show no significant changes in overall mRNA expression levels of the 6 linked genes in age-matched comparisons (respective FDR values are presented in Supplemental Table 3). RNA-seq data were analyzed with age-matched comparisons within generations using a quasi-likelihood *F* test in EdgeR (50). Expression changes with an adjusted *P* value (FDR) of less than 0.05 were considered statistically significant.

**Figure 3:**
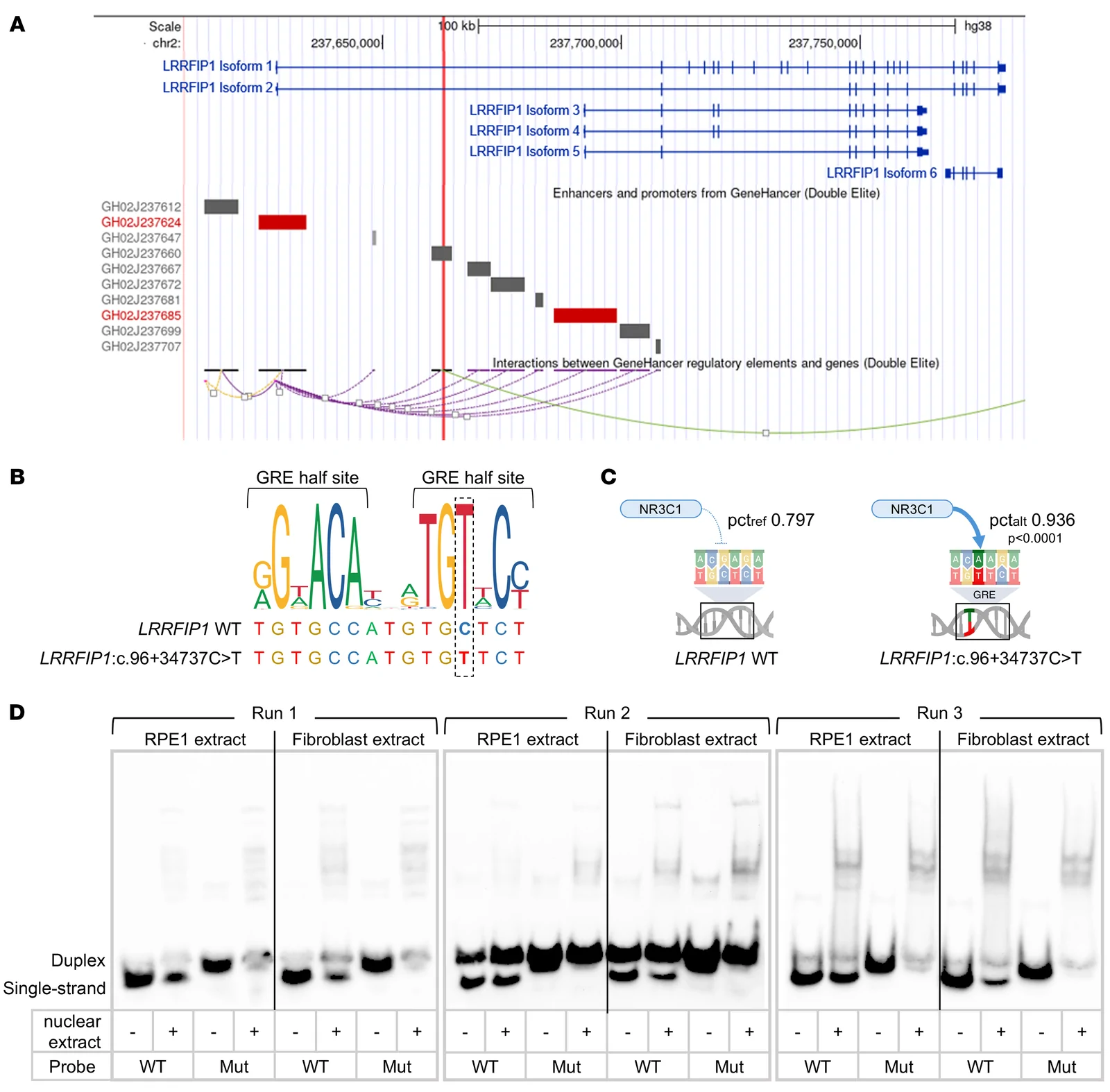
Variant characterization. (**A**) Screenshot adapted from http://genome.ucsc.edu shows the genomic location of the deep intronic patient variant NM_001137550.2(*LRRFIP1*):c.96+34737C>T on chromosome 2q37.3, indicated by the red vertical line (corresponding to GRCh38.p14 NC_000002.12:g.237662477C>T). The variant affects a nucleotide in the high-confidence enhancer GH02J237660 (GeneHancer Track; ref. 12), for which interactions with the TSSs of *LRRFIP1* and *RBM44* are predicted. (**B**) The genomic position of the patient variant NM_001137550.1(*LRRFIP1*):c.96+34737C>T (hg38:2:237662477 C>T) and surrounding nucleotides compared with the consensus transcription factor–binding motif for NR3C1 (JASPAR matrix profile MA0113.4), which is referred to as the GRE motif (65). The GRE motif consists of 2 inverted hexameric sequences (GRE half-sites), separated by a 3 bp spacer. The variant creates a strong GRE half-site. (**C**) Graphical representation of the results of the motifbreakR (13) analysis for the *LRRFIP1* WT sequence and the variant sequence. The patient variant NM_001137550.1(*LRRFIP1*):c.96+34737C>T is predicted to have a strong effect on the binding affinity for NR3C1 (*P* < 0.0001), reaching a pctalt value of 0.936. pctref/pctalt = the score when the sequence contains the reference (pctref) or alternative (pctalt) variant allele, normalized to a scale ranging from 0–1. Normalized motif score (pct) values of 0.85 or higher indicate significant binding probabilities (corresponding to *P* = 0.0001). (**D**) Increased protein binding of *LRRFIP1* c.96+34737C>T oligonucleotides compared with WT oligonucleotides in 3 runs of an EMSA. The biotin-labeled double-stranded oligonucleotide probes showed dissociation into ssDNA oligonucleotides during the run. Following incubation with nuclear extract from RPE-1 or fibroblasts, both the ssDNA WT and mutant probes showed a shift indicating that protein bound to both oligonucleotide probes. The mutant probe clearly shifted more efficiently, indicating stronger protein binding of the mutant probe. This can be appreciated mainly by the lower signal of unshifted mutant probe compared with the WT probe.

**Figure 4:**
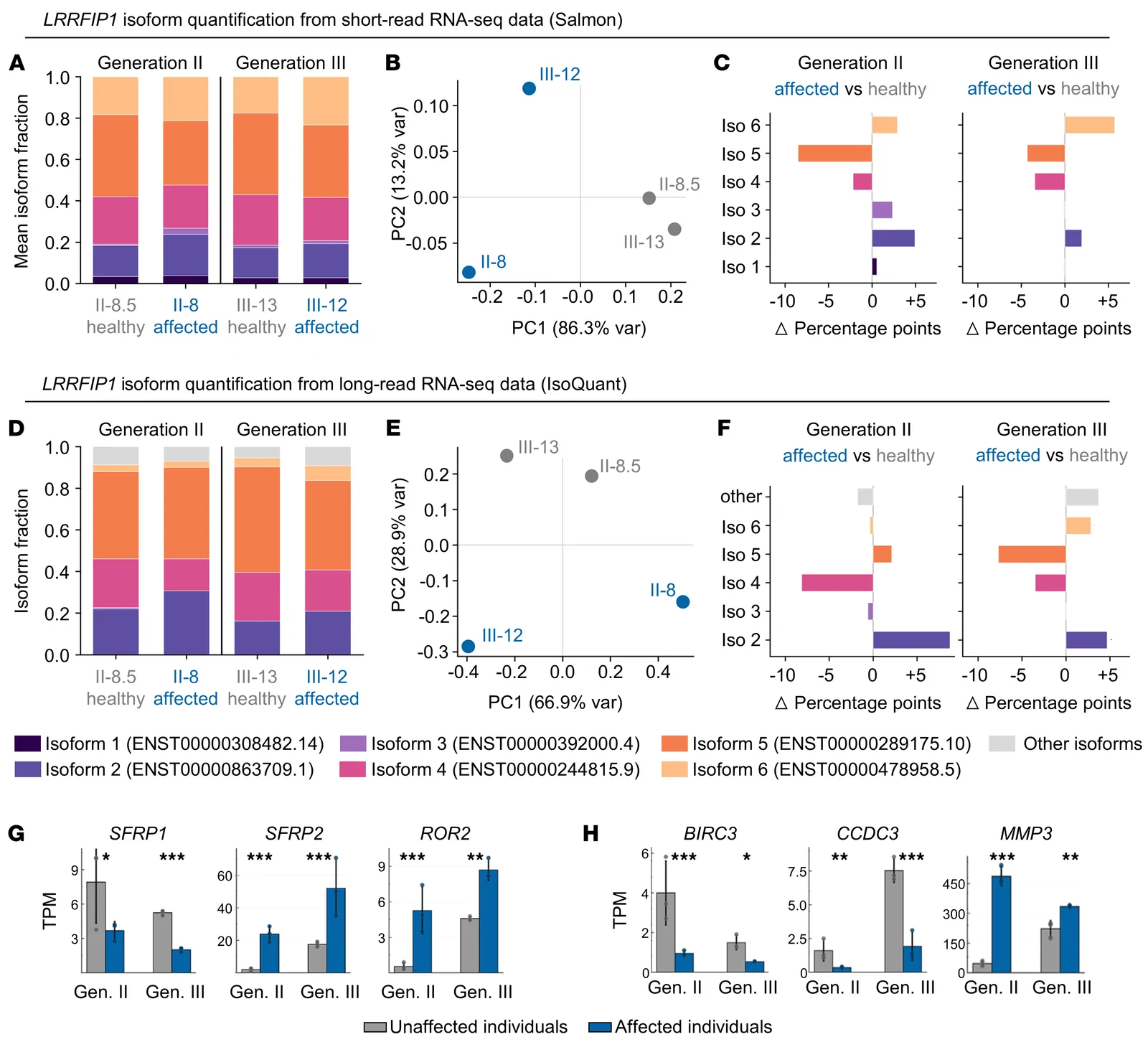
Short- and long-read RNA-seq of cultured fibroblasts from affected and unaffected individuals. (**A**–**F**) Isoform quantification from fibroblasts showing increased expression of isoform 2 and decreased expression of isoform 4 or 5 in affected individuals in long- and short-read RNA-seq data. (**A**–**C**) Short-read *LRRFIP1* isoform quantification with Salmon. Each bar represents the mean across 3 technical replicates. (**A**) Stacked bar charts of *LRRFIP1* isoform fractions indicate a near-absence of isoforms 1 and 3 in all individuals, and a shift of proportions for isoforms 2 and 4–6 in affected individuals. (**B**) Aitchison PCA of isoform composition showing clear separation between affected and unaffected individuals and (**C**) pairwise isoform (Iso) changes between age-matched affected and unaffected individuals showing increased isoform 2 expression and decreased expression of isoforms 4 and 5 in affected individuals. (**D**–**F**) Long-read *LRRFIP1* isoform quantification of full-length nonchimeric PacBio reads using IsoQuant. Stacked bar charts of *LRRFIP1* isoform fractions (**D**), PCA (**E**), and pairwise age-matched comparisons (**F**) confirm short-read RNA-seq results and indicate that the decrease in isoforms 4 and 5 was mainly driven by isoform 5 in the younger generation and isoform 4 in the older generation. (**G** and **H**) DEG analysis of short-read RNA-seq data showed 81 significantly DEGs, including genes involved in canonical and noncanonical Wnt signaling (**G**) and inflammatory TNF-α and NF-κB signaling (**H**). TPM and FDR values for the DEGs shown are provided in Supplemental Table 5, and the complete DEG list is shown in Supplemental Table 10. RNA-seq data were analyzed with age-matched comparisons within generations using a quasi-likelihood *F* test in EdgeR (50). Generation II (Gen. II): affected individual II-8 compared with unaffected individual II-8.5. Generation III: affected individual III-12 compared with unaffected individual III-13. Nonsignificant, FDR ≥0.05. Significant, *FDR < 0.05, **FDR < 0.01, and ***FDR < 0.001.

**Figure 5:**
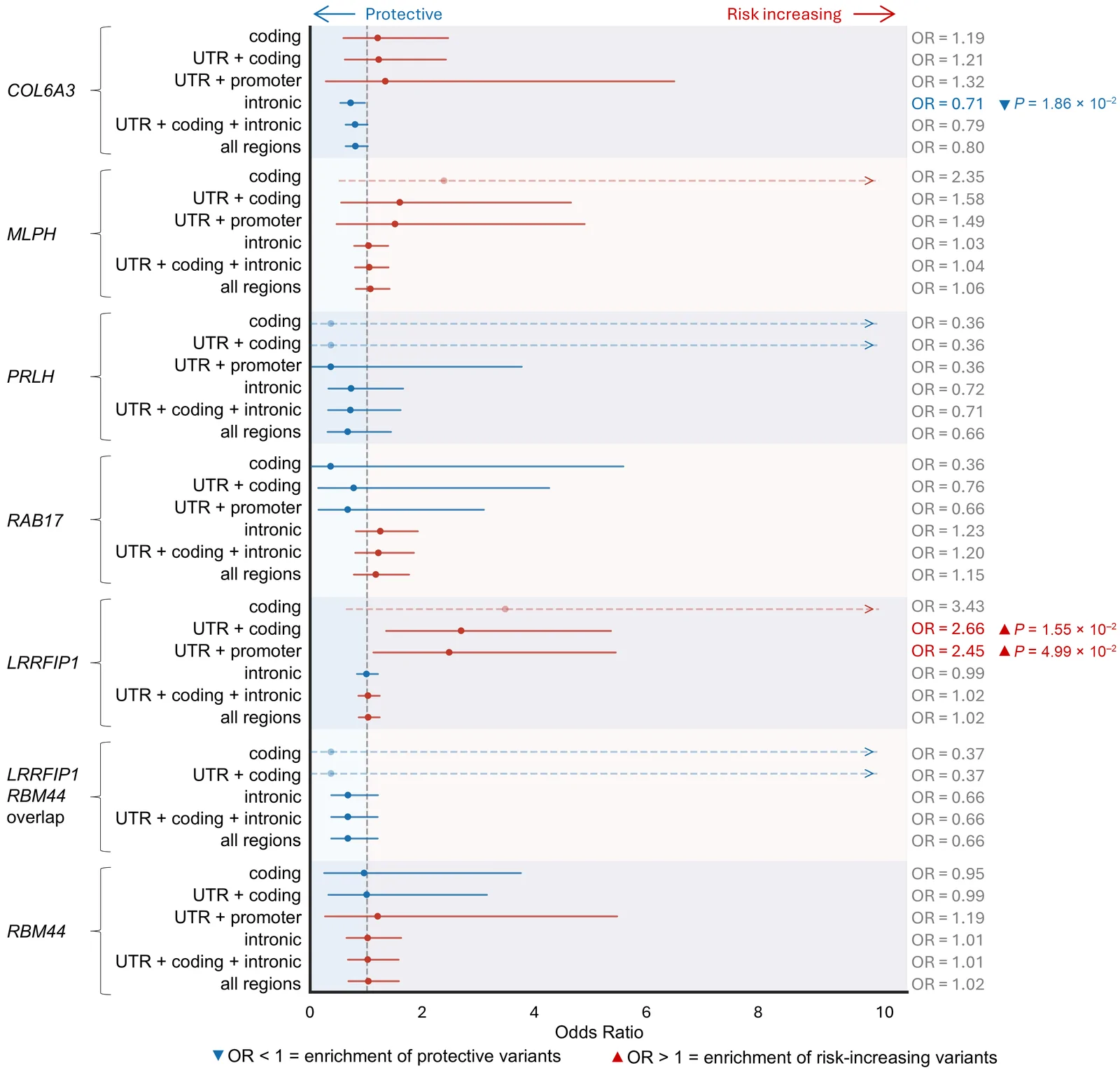
Rare variant ADD burden with directionality effects across the 6 genes within the linked region in the UK Biobank cohort. Rare variant ADD burden test with directionality analysis showing a significant enrichment of rare risk-increasing variants in *LRRFIP1* in individuals with a diagnosis of scoliosis in the UK Biobank Cohort. The forest plot shows ORs and 95% CIs from ADD burden tests with near-private rare variants (MAC ≤5) with REGENIE for the 6 protein-coding genes within the linked interval on chromosome 2q37. This configuration captures the cumulative effect of the rare variants in all 6 genes and the genomic overlap region of *LRRFIP1* and *RBM44* across 6 annotation masks (42 tests total). The 6 masks per gene represent the variant localization within the respective gene: “coding” refers to the protein-coding sequence, “UTR” refers to 5′ and 3′ UTR, “promoter” refers to the promoter region defined as the 1 kb region upstream of the TSS, “intronic” refers to intronic sequence. An OR of greater than 1 (red color) indicates a risk-increasing burden (enrichment of rare variants in individuals with scoliosis). An OR of less than 1 (blue color) indicates a protective burden (enrichment of rare variants in individuals without scoliosis). The dashed vertical line indicates OR = 1 (null). CIs were capped at 10; arrows indicate CIs extending beyond the range shown. *P* values are displayed for masks reaching nominal significance (*P* < 0.05). *LRRFIP1* CDS + UTR (OR = 2.66, *P* = 0.016) and UTR + promoter (OR = 2.45, *P* = 0.050) show risk-increasing signals, while *COL6A3* intronic variants show a protective effect (OR = 0.71, *P* = 0.019).

**Figure 6:**
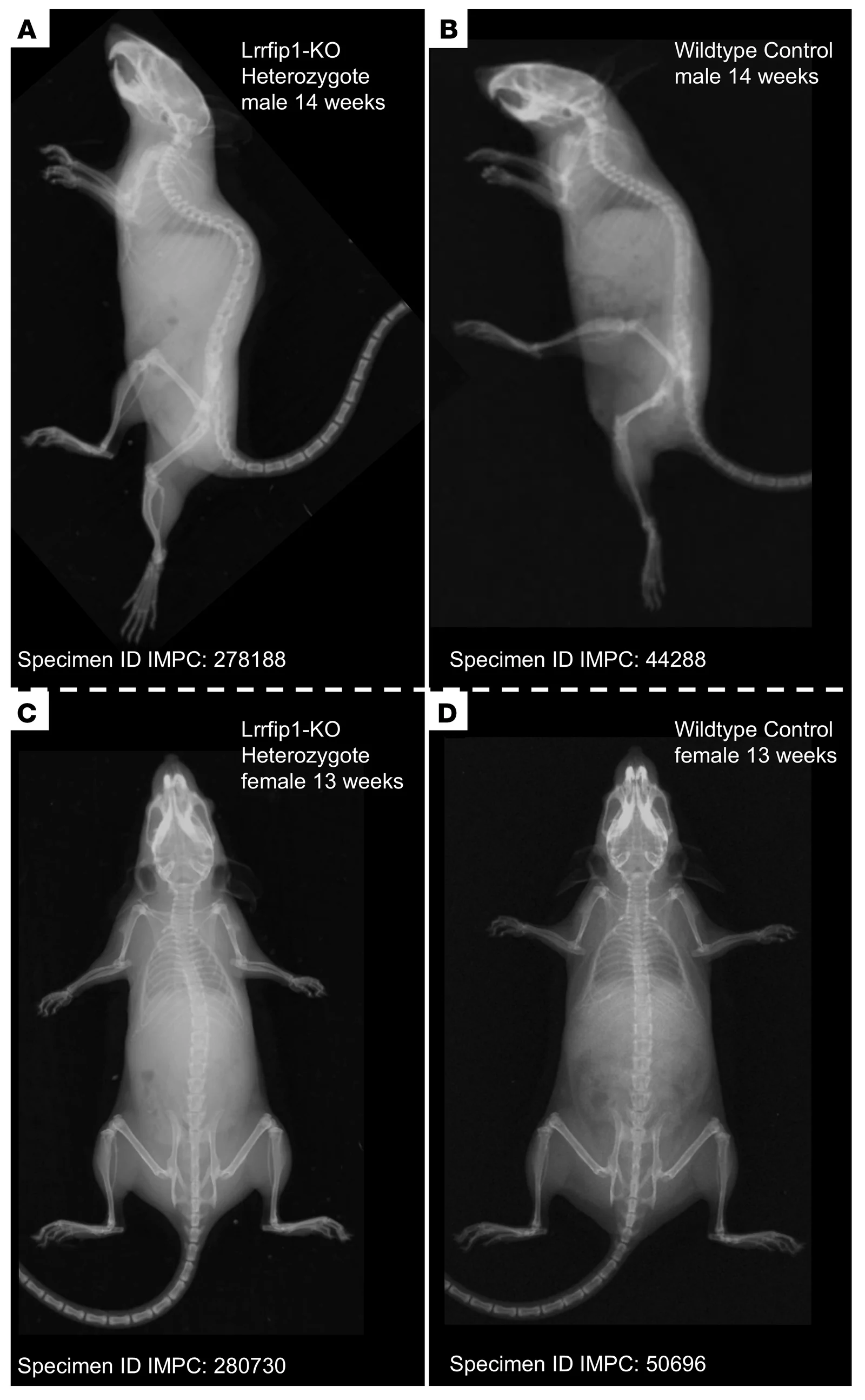
*Lrrfip1*-deficient mice are affected by idiopathic kyphoscoliosis. Lateral radiograph of a heterozygous Lrrfip1-KO mouse (14-week-old male, IMPC specimen ID: 278188) with marked kyphosis and cervical hyperlordosis with a kyphosis index of 2.85 (**A**), compared with the lateral radiograph of an age- and sex-matched WT control mouse (14-week-old male, IMPC specimen ID: 44288) showing physiological spinal curvature with a kyphosis index of 3.105 (**B**). Lower kyphosis indexes indicate more severe kyphosis. Anteroposterior radiograph of a heterozygous *Lrrfip1*-KO mouse (13-week-old female, IMPC specimen ID: 280730) with left convex thoracic scoliosis with a scoliotic cobb angle of 15.5° (**C**), compared with the anteroposterior radiograph of an age- and sex-matched WT control mouse without scoliosis (13-week-old female, IMPC specimen ID: 50696) (**D**). Images were retrieved from the IKMC/IMPC (www.mousephenotype.org), available under the Creative Commons Attribution 4.0 International license (CC-BY 4.0).

## Version 1 09/29/2026

In-Press Preview

## Version 2 11/16/2026

Electronic publication
