# Supplementary material for: Rare LRRFIP1 variants contribute to the pathophysiology of severe early-onset scoliosis and common scoliotic phenotypes
Source: J Clin Invest. 2026 Sep 29;136(22):e202751. doi: 10.1172/JCI202751 (PMC13769427; doi:10.1172/JCI202751)
Supplement: Supplementary file 1 [file jci-136-202751-s007.pdf]

Supplemental Information: Table of Contents

1 Phenotype descriptions of selected *LRRFIP1*-EOS affected individuals

2 Genes within linked region (besides *LRRFIP1*)

3 Transcription factor binding site analysis: motifbreakR
- Table S1

4 EMSA supplemental information
- Figure S1

5 UK Biobank rare variant enrichment analysis supplemental information
- Figure S2

6 *Lrrfip1* mouse model supplemental information
- Figure S3
- Figure S4

7 RT-PCR analysis in patient fibroblasts
- Figure S5

8 Western blot
- Figure S6

9 Antibodies

10 Eukaryotic cell lines

Supplemental Figures S7 A-F: enlarged versions of radiographic images shown in main figure 1

Supplemental Figures S8-11

- Figure S8: Haplotype analysis of the EOS candidate region on chromosome 2q35q37
- Figure S9: DEG analysis from cultured fibroblasts
- Figure S10: *LRRFIP1* isoform composition in iPSCs
- Figure S11: *NR3C1* expression in iPSCs and fibroblasts

Supplemental Tables

- Table S2: Isoform expression changes compared to age-matched wildtype controls in relation to *NR3C1* expression
- Table S3: FDR values for expression differences of the six genes within the linked region
- Table S4: Overview of pairwise comparison of isoform expression changes in affected individuals compared with age-matched unaffected individuals in both short- and long-read RNA-seq data
- Table S5: FDR values of the differentially expressed genes shown in main Figure 4 (G and H)
- Table S6: Top 10 most enriched Gene Ontology Biological Processes using the significantly differentially expressed genes from RNA sequencing data

from individuals with *LRRFIP1*-EOS compared to unaffected generation-matched relatives
- Table S7: Top 10 most enriched Reactome Pathways using the significantly differentially expressed genes from RNA sequencing data from individuals with *LRRFIP1*-EOS compared to unaffected generation-matched relatives
- Table S8: Scoliotic Cobb angle, Kyphotic Index and Hyperlordosis measurements of Lrrfip1-knock out mice
- Table S9: Scoliotic Cobb angle, Kyphotic Index and Hyperlordosis measurements of wildtype control mice

Supplemental references

1 Phenotype descriptions of selected *LRRFIP1*-EOS affected individuals

The index patient III-12 first presented for clinical and genetic evaluation at the outpatient medical genetics clinic at the age of 11 years due to his pronounced kyphoscoliosis. He was an overall healthy boy with normal psychomotor development and no history of significant disease, except for progressive early-onset kyphoscoliosis. The first clinical signs of scoliosis had been noticed at the age of 3 years. Despite early physical therapy and brace therapy, the kyphoscoliosis progressed significantly during childhood. At the age of 11, the patient had a thoracolumbar kyphosis (T12-L3, Cobb angle of 56°) with a visible Gibbus deformity, and a right convex scoliotic deformity (T11-L3, Cobb angle of 20°) accompanied by marked stiffness of the thoracic and lumbar spine in all directions with a fingertip-to-floor distance over 35 cm. At the age of 14 years, kyphoscoliosis had further worsened reaching a maximum kyphotic Cobb angle of 110° (T10-L3) and a scoliotic Cobb angle of 50° (T12-L4), requiring surgical correction at age 14. Besides the kyphoscoliosis and intermittent knee and groin pain, the patient reported no complaints and had always been in good overall health. He had never sustained any bone fractures and reported no symptoms of inflammatory back or joint pain and no symptoms of generalized rheumatologic or autoimmune disease (no signs of peripheral arthritis, no history of enthesitis, dactylitis, anterior uveitis, psoriasis or colitis). He had been diagnosed with femoroacetabular impingement due to camshaft (CAM) morphology of the left and right femoral neck. At the age of 11 years, we noted tall stature for his age (height 164.5 cm, >97th centile, +2.5SD)) and macrocephaly (OFC 57 cm, >97th centile, +2.5 SD), a decreased arm span-to-height ratio of 0.96 (arm span 158.6 cm), and relative brachydactyly with a palmar length of 17.8 cm (95th centile) and length of the third finger of 7.2 cm (48th centile). Bilateral sandal gaps and broad halluces were noted. The patient had a long face with malar flattening, hooded eyelids, downslanted palpebral fissures, anteverted nares, and a high-arched and narrow palate. We noted normal joint mobility except for significant rigidity of the spine, and no obvious signs of generalized connective tissue abnormalities or signs of arthritis. He had no apparent muscle weakness or neurologic symptoms that would be suggestive of a neuromuscular or syndromic cause of his kyphoscoliosis. At the last

examination at age 24, 10 years post-surgery, he was 185.5cm tall (90th centile, + 1.25 SD) and had mild macrocephaly (OFC 60.4cm, >97th centile, + 2.22 SD).

Imaging (X-rays, CTs, MRIs, Figure 1A-H) of patient III-12 were obtained at ages 4, 5, 6, 11, 12, 13, 14. At age 4 years, X-Rays of the spine show rounded deconfiguration of all vertebrae and markedly thinned intervertebral spaces with erosions of the vertebral endplates and mild scoliosis of the upper lumbar spine. During further childhood, rapidly progressive kyphoscoliosis of the lumbar spine is evident with progressive erosive changes of the vertebral end plates leading to final ankylosis in the lumbar spine as well as gradual expansion of the erosive changes over time to the thoracic and finally lower cervical spine with the intervertebral spaces completely obliterated in the lumbar and lower thoracic spine. X-Rays of other skeletal elements show no or only mild deconfigurations: Coxa valga with big femoral heads, relatively broad appearance of ribs, clavicles and pubic rami. Outside of the spine, no major dysplastic elements and no erosive, inflammatory or degenerative changes are apparent.

Patient III-14 had a history of back stiffness, scoliosis, and back pain. At the age of 22, he was tall (192.7 cm, >97th centile, +2.22 SD), while his head circumference was within the normal range (OFC 57.4 cm, 60th centile, +0.24 SD). He displayed severe spinal stiffness in all directions, with almost complete lack of flexion in the thoracic and lumbar spine and a mild scoliotic curvature of the thoracic spine with asymmetry of the scapulae (Fig. 1K, L). He had a flat chest with slight pectus excavatum. He shared some of the same facial features with patient III-12 (long face, downslanted palpebral fissures, hooded eye lids, anteverted nares). He too had a high-arched and narrow palate, a long neck, bilateral sandal gaps, and broad halluces. Besides scoliosis, he was healthy without significant history of disease or any known organ pathologies. He had no apparent muscle weakness or neurologic symptoms. Radiographs of his spine taken at age 10 show complete loss of the physiological spinal curvature with thoracic hypokyphosis and flattening of the lumbar lordosis, significantly reduced intervertebral spaces in the thoracic and lumbar spine, multisegmental endplate irregularities with significant erosive changes and ankyloses of the lumbar and lower thoracic vertebral bodies. Radiological

assessment of bone age at age 10 showed mildly disharmonic osseus maturation with advanced maturation of the carpal bones with normal configuration of the hand skeleton.

The unaffected fraternal twin brother (III-13) of patient III-14 was not affected by any spinal deformity or stiffness. He was of normal stature (173 cm, 35th centile) and reported to be completely healthy.

Patient II-8 (the father of III-12, III-13, III-14) presented with the same clinical picture as his two affected sons in terms of spinal deformity and stiffness manifesting in early childhood. He had a visible gibbus deformity and significant rigidity of the sine. He used to be 185 cm tall (85th centile, +1.19 SD), but at the last examination at age 61, his height had reduced to 181.9 cm (75th centile, +0.77 SD). His OFC was 57 cm (50th centile, -0.02 SD). He had a long neck and a high arched palate, and he shared the same facial features with his two affected sons (long and narrow face, downslanted palpebral fissures, hooded eyelids, anteverted nares) and he too appeared to have relatively short fingers, and he additionally had brachydactyly type D. He had developed coronary artery disease requiring implantation of multiple coronary stents at the age of 60 and he suffered from mild age-related hearing loss. Otherwise, he stated that he was healthy. He had no history of bone fractures, no rheumatologic or neurological diseases, and no vision problems.

Patient II-1, a 76 year-old male at the time of the interview, had severe early-onset kyphoscoliosis that caused sensory disturbances in his legs and breathing problems, but had never been surgically corrected. When he was younger, he was 184 cm tall (83rd centile, +1.05 SD). He reported pain in his legs and joints. Except for severe kyphoscoliosis beginning in early childhood, he had always been healthy. He developed osteoporosis and mild age-related vision and hearing impairment only at an advanced age. X-Ray of the spine showed significant thoracolumbal kyphoscoliosis with severe endplate erosion and ankyloses of thoracic and lumbar vertebrae with relatively normal appearing cervical spine and no signs of sacroiliitis (images not shown due to poor quality).

Patient II-5, a 69-year-old female, had severe early-onset scoliosis for which she underwent corrective surgery. X-Rays taken at the age of 51 years decades after corrective surgery had been performed (Figure 1N) show an S-shaped scoliosis involving the thoracic and lumbar spine with a right convex thoracic curve with a Cobb angle of 64° and a left convex lumbar curve with a Cobb angle of 53°(Figure 1N), and loss of the cervical lordosis with obliterated intervertebral spaces, extensive erosions of the vertebral endplates and long stretches of ankylosis in the thoracolumbar segments (lateral X-Ray not shown due to poor quality). In her younger years she was 172cm tall (90th centile, +1.35 SD), at the time of the interview she was 168cm tall. At age 65, she had undergone aortic valve replacement. Otherwise, she reported no chronic diseases or significant health problems. She denied having any neurological symptoms or weakness. She reported normal hearing and mild age-related vision loss.

Patient II-9, a 60-year-old male, also had severe early-onset scoliosis. He had been treated with a rigid brace in childhood but required surgery because of continuous worsening of the deformity. He reported sensory disturbances, pain, and breathing problems. He also developed osteoarthritis in the hip joints. He used to be 190 cm tall (95th centile, +1.85 SD) and reported that he had no health problems other than the scoliosis and its secondary problems.

Patient III-15, a 25-year-old female, had received physical therapy since early childhood due to her early-onset scoliosis. Her adult height was 178cm (>97th centile, +2.26 SD). She was wearing a 13 mm corrective insole in her left shoe to compensate for the significant pelvic asymmetry due to scoliosis. Besides scoliosis and mild astigmatism and myopia, she had always been healthy.

2 Genes within linked region (besides *LRRFIP1*)

Within the linked region on chromosome 2q37.3, no gene previously known to cause isolated EOS or any other isolated spinal pathology was located. We evaluated all genes within the peak linkage region regarding their function, known disease associations and possible pathomechanistic links to chondrodysplastic-erosive EOS:

COL6A3 (OMIM:120250) is a known disease gene associated with autosomal-dominant and autosomal-recessive forms of muscular dystrophies, which are frequently associated with neuromuscular scoliosis. However, the patients we describe here have no muscular weakness or any other signs of neuromuscular impairment. Genome sequencing identified no likely disease-causing rare variant. The heterozygous variant NM_004369.4(*COL6A3*):c.5610C>A (p.Ser1870Arg) was detected, but this variant was excluded from further considerations as it is present at a high rate in the healthy population (GnomAD v4.1.0 global minor allele frequency of 0.11%) and several homozygous individuals are reported in the gnomAD database. Furthermore, this variant has been submitted to ClinVar (Accession: VCV000094947.53) as benign (2 times), likely benign (2 times), uncertain or conflicting (3 times).

MLPH (OMIM:606526) encodes the protein melanophilin and is associated with the autosomal-recessive Griscelli Syndrome, type 3 (OMIM#607227). Griscelli Syndrome type 3 is a condition without any skeletal involvement or putative links to vertebral development. As the gene is associated with an autosomal-recessive phenotype, we deemed *MLPH* an unlikely candidate gene for autosomal-dominant EOS.

PRLH (OMIM:602663) encodes the prolactin-releasing peptide and has not been associated with a mendelian phenotype yet. *PRLH* is thought to play a role in the regulation of hunger and satiety and regulation of other hypothalamic signaling pathways, yet no plausible links to EOS became evident in a literature review.

RAB17 (OMIM:602206) encodes a small epithelial cell-specific GTPase from the Rab family. Rab GTPases are important for the regulation of membrane trafficking. *RAB17* has not been linked to a Mendelian phenotype yet and a literature review did not reveal any plausible links to EOS.

RBM44 (OMIM:621038) encodes an RNA-binding protein which is predicted to be involved in mRNA splicing as part of the catalytic step 2 spliceosome. *RBM44* has not yet been described

as a disease-causing gene and no apparent links to scoliotic or any other skeletal phenotypes have been reported.

2q37 Deletion syndrome and *HDAC4:* The linked region partially overlaps with the chromosomal region deleted in the 2q37 deletion syndrome (OMIM#600430). Patients with 2q37 deletion syndrome are affected by a neurodevelopmental disorder and frequently are affected by skeletal anomalies including brachydactyly and sometimes scoliosis. However, the gene *HDAC4*, which is the main phenotype defining gene in the 2q37 microdeletion syndrome (1) is located outside the linked region.

3 Transcription factor binding site analysis: motifbreakR

Table S1 MotifbreakR analysis for the patient variant and neighboring variants present in the UK Biobank

MOTIF (gene symbol)	Wildtype (pctref)	chr2:237662472 A>G (Δpct)	chr2:237662475 T>G (Δpct)	chr2:237662476 G>A (Δpct)	chr2:237662476 G>T (Δpct)	chr2:237662477 C>T (Δpct)	chr2:237662479 C>T (Δpct)
MITF	0.993561582	-0.186239546	-0.186239546	-0.202662816	-0.202662816		
NR2F6	0.768121495						0.113706916
NR3C1	0.797097302					**0.138425752**	
YY1	0.814065124				0.108566859		

The table shows that the effect of the patient variant (hg38:2:237662477 C>T) is unique within this genomic region, as neighboring variants lead to different effects. Variants affecting nucleotides chr2:237662472, chr2:237662475, chr2:23766247776 are predicted to decrease binding affinity for MITF, the chr2:237662476 G>T substitution additionally strengthens binding to YY1, and the chr2:237662479 C>T variant strengthens binding affinity to NR2F6. pctref / pctalt = The score as determined by the scoring method, when the sequence contains the reference (pctref) or alternative (pctalt) variant allele, normalized to a scale from 0 - 1. The threshold was set to 0.85, corresponding to a p-value of 1e-4. PCT values ≥0.85 indicate significant binding probabilities, only effects passing the threshold are reported in the table; neutral effects ($\Delta p\_i<0.4$) are not shown.

4 EMSA supplemental information

While we observed no shift of the DNA duplex probe for the wildtype or the mutant condition, a strong shift was present for the lower band of the free probe, corresponding to single strand (ss) DNA. The ssDNA band is the result of duplex dissociation during the electrophoresis step, which is frequently observed in EMSA experiments, including the positive controls of the used EMSA LightShift™ kit (see kit manual). We confirmed the identity of the duplex and ssDNA bands for our probes by gel electrophoresis where we separately analysed duplex DNA and ssDNA oligonucleotides, as well as a mix of forward and reverse oligonucleotides without prior alignment. This analysis confirmed that the lower band of free probe which showed a shift in the EMSA conditions with nuclear extract is indeed ssDNA. Following incubation with nuclear extract from RPE-1 or fibroblasts cells, both the ssDNA wild-type and mutant probes showed a shift indicating that protein bound to both oligonucleotide probes; however, the mutant probe was clearly shifted more efficiently, indicating stronger protein binding to the mutant probe. This can be appreciated mainly by the lower signal of unshifted mutant probe compared to the wildtype probe, whereas the conversely higher signal for shifted mutant probe is not as evident. This suggests that a significant portion of the complexes which formed between nuclear proteins and ssDNA probe could not efficiently enter the gel but were retained in the wells. In addition to the EMSA with nuclear extracts, the assay was also performed with purified NR3C1-FLAG protein obtained through immunoprecipitation (Figure S1). When only NR3C1-FLAG was added, no shift of either the wild-type or the mutant DNA probe was observed, possibly because critical accessory protein interactions were absent in this setup.

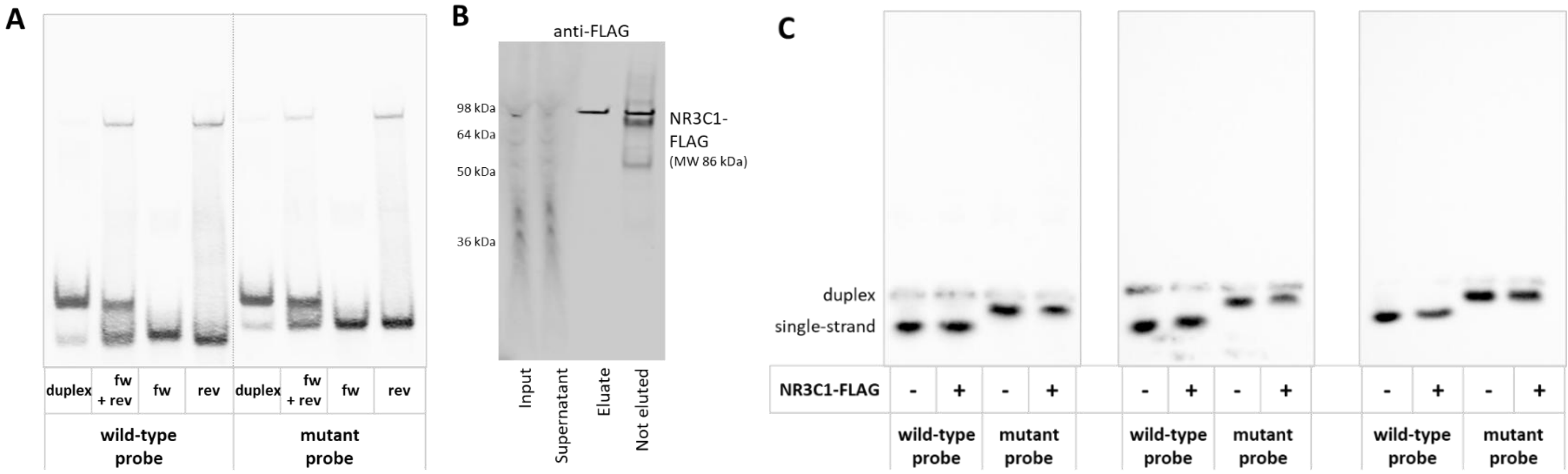

Figure S1 EMSA

Panel A shows the comparative analysis of duplex and single strand DNA probes by PAGE. Biotin-labeled double-strand DNA oligonucleotide probes harboring the *LRRFIP1* patient variant c.(96+34737C>T) (mutant probe) or the respective wild-type sequence (wild-type probe) were run on a polyacrylamide gel next to a mix of the respective single strand probes without prior annealing, or only forward or reverse strand.

Panel B shows a confirmatory analysis of NR3C1-FLAG expression in HEK293 cells purified by immunoprecipitation via the FLAG-tag. Elution was achieved using an excess of free FLAG peptide. Western Blot analysis of NR3C1-FLAG immunoprecipitation samples (input, supernatant, eluate, and not-eluted sample) with anti-FLAG antibody confirmed the presence of NR3C1-FLAG protein in the eluate.

Panel C Biotin-labeled double-strand DNA oligonucleotide probes harboring the *LRRFIP1* variant c.(96+34737C>T) or the respective wild-type sequence were incubated together with or without immunopurified NR3C1-FLAG protein sample and then analysed by Electromobility shift assay (EMSA) in n=3 independent experiments.

5 UK Biobank rare variant enrichment analysis supplemental information

UK Biobank is a large-scale biomedical database and research resource containing anonymized genetic, lifestyle and health information from half a million UK participants including whole genome sequencing data. UK Biobank recruited 500,000 people aged between 40-69 years in 2006-2010 from across the United Kingdom (UK). With their consent, they provided detailed information about their lifestyle, physical measures and had blood, urine and saliva samples collected and stored for future analysis. UK Biobank's research resource is a major contributor in the advancement of modern medicine and treatment, enabling better understanding of the prevention, diagnosis and treatment of a wide range of serious and life-threatening illnesses – including cancer, heart diseases and stroke. UK Biobank is generously supported by its founding funders the UK Medical Research Council (MRC) and Wellcome, as well as the British Heart Foundation, Cancer Research UK, the National Institute of Health Research (NIHR) and UK Research and Innovation (UKRI).

All rare variants within the analyzed gene sets were functionally annotated using the Ensembl Variant Effect Predictor (VEP) version 115. VEP was run via Docker (ensemblorg/ensembl-vep) in offline mode with locally cached databases (GRCh38 assembly), using the --everything flag to retrieve all available annotations, including SIFT, PolyPhen, allele frequencies from gnomAD and 1000 Genomes, ClinVar significance, and regulatory feature overlap. The input VCF contained all variant sites from the cleaned whole-genome sequencing (WGS) dataset within the six gene regions (see Regenie Burden Testing Methods for variant extraction and quality control). VEP was executed with tabular output format (--tab), six parallel threads (--fork 6), and forced overwrite (--force_overwrite). No statistics summary was generated (--no_stats) as downstream analyses were performed with custom scripts. VEP assigns consequences for every overlapping transcript, producing approximately 11 million annotation lines for the six gene regions. To obtain a single representative consequence per variant, the output was filtered to retain only canonical transcripts (CANONICAL=YES column). This pre-filtering step reduced the dataset to approximately 240,000 lines. Where a variant had multiple

consequence terms for the canonical transcript (comma-separated in VEP output), the most severe consequence was selected according to Ensembl severity ranking.

VEP consequence terms were grouped into biologically meaningful categories for visualization and summary statistics. Variant types were classified from the variant identifier (chr:pos:ref:alt format) as single nucleotide variants (SNV; equal-length single-base ref and alt), deletions (ref longer than alt), insertions (alt longer than ref), or multi-nucleotide variants (MNV; equal-length multi-base substitutions). To characterize the profile of variants carried by scoliosis cases versus controls, allele frequencies were computed separately for cases and controls using PLINK2 --freq with --keep to subset samples by phenotype status. Variants were then classified into three mutually exclusive carrier categories based on allele count in each group (case only, shared, control only) (Figure S2).

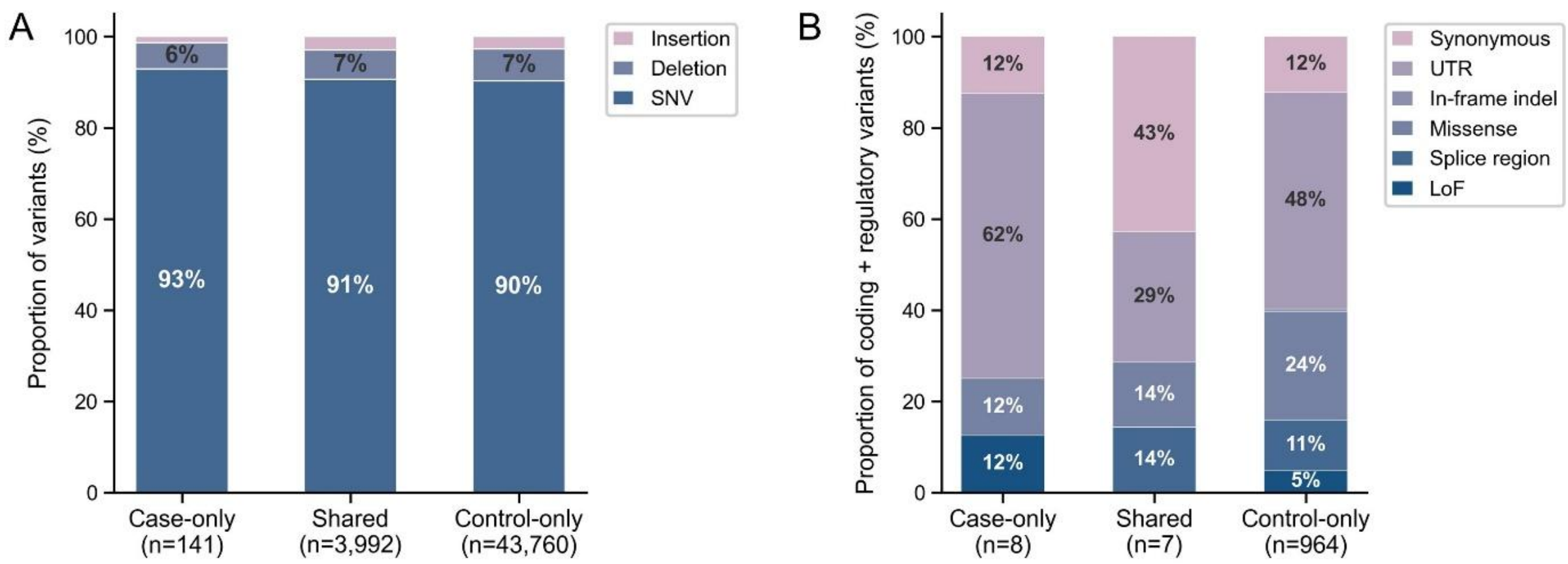

Figure S2 UK Biobank Rare Variant Enrichment Analysis: variant effect predictor analysis
(A) Variant types (SNV, deletion, insertion) across all rare variants in *LRRFIP1* including intronic regions, stratified by disease status: case-only (n = 141 rare variants), shared (n = 3,992 rare variants), and control-only (n = 43,760 rare variants). The variant type distribution is similar across carrier categories, with SNVs predominating (90-93%). **(B)** Stacked bar chart showing the proportional distribution of VEP-annotated consequence categories among rare (MAC ≤ 5) coding and regulatory variants in *LRRFIP1* stratified by disease status: case-only (n = 8 rare variants), shared between cases and controls (n = 7 rare variants), and control-only (n = 964 rare variants). Intronic variants were excluded. Case-only variants are enriched for UTR (62%) and loss-of-function (LoF; 12%) compared to control-only variants (48% and 5%,

respectively), consistent with regulatory and damaging variant enrichment driving the observed burden signal in Figure 5 (OR = 2.66, p = 0.015 for the CDS + UTR mask).

6 *Lrrfip1* mouse model supplemental information

We found that Lrrfip1$^{tm1.1(KOMP)Wtsi}$ mice (KO mice) had a significantly (p=0.0151) higher prevalence of scoliosis with 8 out of 18 affected KO mice (44%) versus 6 out of 46 affected WT mice (13%). The mean Cobb angles were significantly greater in the group of KO mice (N2=18, mean Cobb angle = 9.06°, SD 5.70°, df_2=17) compared with the WT mice sample (N1=46, mean Cobb angle = 4.9°, SD 3.85°, df_1=45) with a t-value of -3.3688 and a p-value of .000651 (significant at $p < .05$) in an independent T-test, with a large effect size (Hedges g= 0.9380). The calculation of a one-tailed Mann-Whitney U test showed a statistically significant difference in Cobb angles between both groups with significantly higher values among the KO mice, U = 593.5, Z = -2.67, p= .00379, significant at p < .005. 16 of 18 KO mice (88 %) had cervical hyperlordosis, which was not detected in any of the 46 WT mice (0 %) (Fishers exact test p= < 0.00001, significant at p <.05). An independent T-test showed that the mean kyphosis index in the KO sample ($N_{2=}$18, mean kyphosis index = 2.92, SD 0.21, df_2=17) was significantly lower than in the WT sample ($N_{1=}$44, mean kyphosis index = 3.15, SD 0.27, df_1=43), representing a more severe mean kyphotic deformity in KO mice with a t-value of 3.26231 and a p-value of .000913 (significant at $p < .05$), with a large effect size (Hedges g = 0.912757). Likewise, the one-tailed Mann-Whitney U test showed a statistically significant difference in kyphosis indexes with lower kyphosis indexes among the KO mice, U = 192.5, Z = 3.14814, p = .00082, significant at p < .005.

Significant phenotypes reported by the IMPC on their website (https://mousephenotype.org) (2) were abnormal skin coloration and decreased levels of circulating triglyceride levels in male heterozygous Lrrfip1$^{tm1.1(KOMP)Wtsi}$ mice, decreased levels of alkaline phosphatase, calcium and total protein in female heterozygous Lrrfip1$^{tm1.1(KOMP)Wtsi}$ mice and preweaning lethality with incomplete penetrance in homozygous Lrrfip1$^{tm1.1(KOMP)Wtsi}$ mice.

A number of neuromuscular and neurologic screens applied by the IKMC/IMPC (grip strength, gait abnormalities, open field tests, tremor, acoustic startle and pre-pulse inhibition testing,

aggression, spontaneous movement) did not show any significant neurologic or muscular differences between Lrrfip1$^{tm1.1(KOMP)Wtsi}$ mice and WT mice (2).

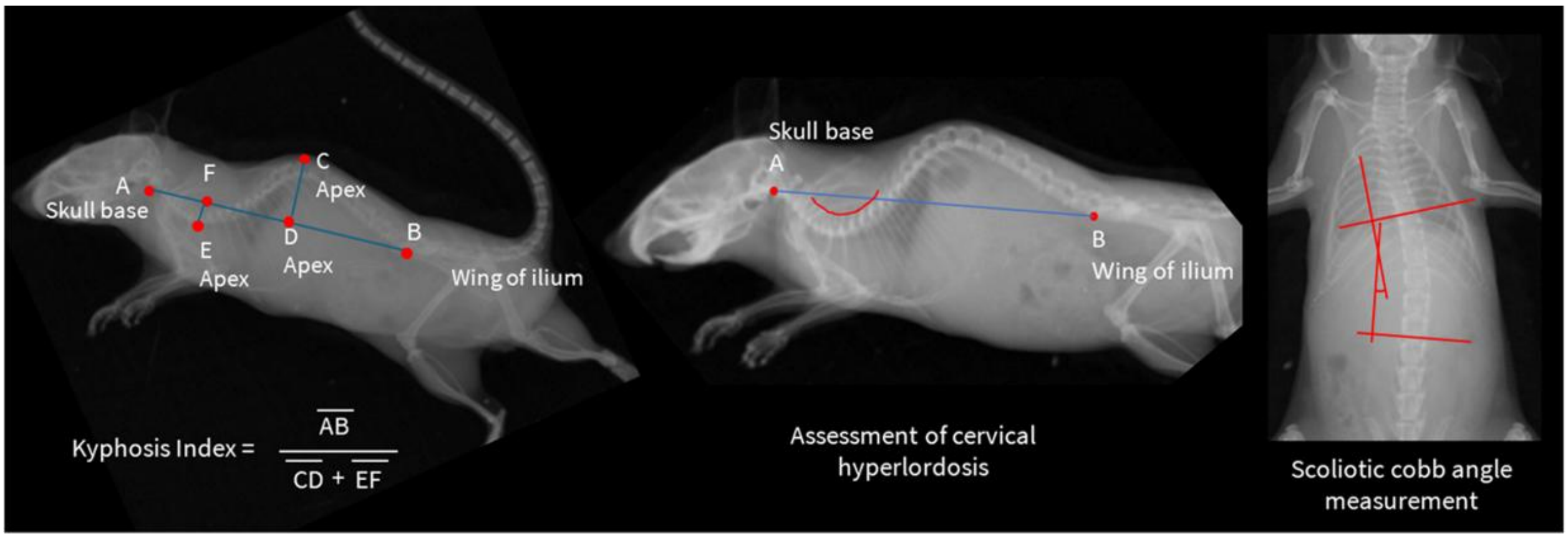

Figure S3 Measuring methods for kyphoscoliosis assessment in *Lrrfip1*-knock out and wildtype control mice

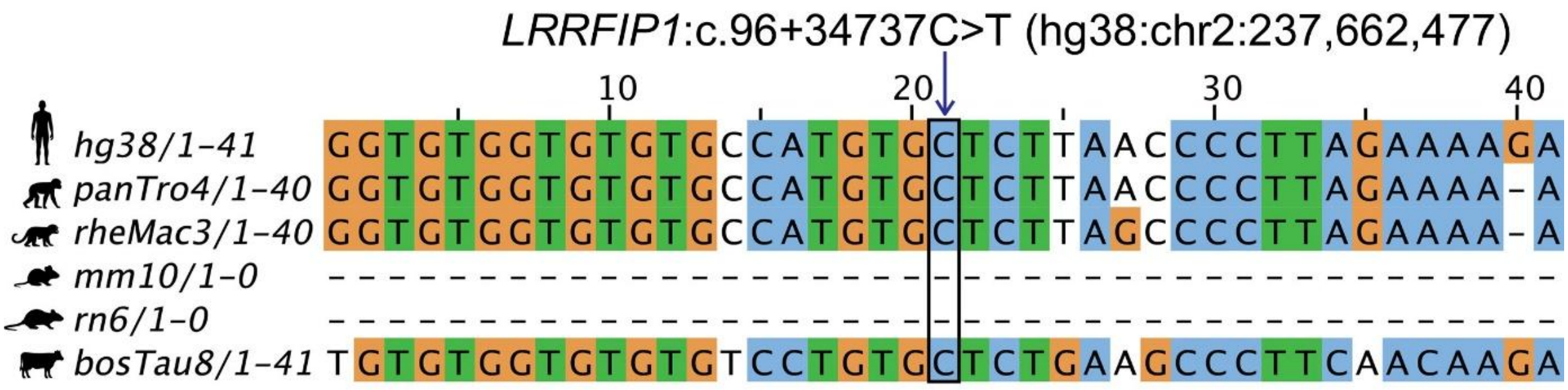

Figure S4 Conservation of variant position in vertebrates

Cross-species alignment at the *LRRFIP1* c.96+34737C>T variant position (UCSC genome browser Multiz 100-way vertebrate alignment, hg38, ±20 bp centered on chr2:237,662,477) is visualized using Jalview (3). The variant position (arrow, boxed) is conserved across primates (human, chimpanzee, rhesus macaque) but lacks aligned sequence in rodents (mouse mm10, rat rn6). Cow (bosTau8) shows moderate conservation of this intronic segment, but conservation of the nucleotide affected by the patient variant.

7 RT-PCR analysis in patient fibroblasts

1.7 µg of total RNA (passages 2-4) were reverse transcribed using the SuperScript™ III First-Strand Synthesis System with random hexamer primers (Thermo Fisher Scientific). *LRRFIP1* isoform specific primer pairs as published in previously (4) were purchased from Microsynth. Iso1-fw: CGTAAGGAGTGAACGGGATG; Iso1-rev: GTC TGTC CCG TTT TCC AAG A; NM_001137551-fw: GGG AGA TCG ACT GTT TGA GC; NM_001137551-rev: 5′CCG TTC ATC AAC CAG CTT TT; Iso3-fw: AGA TTC AGG ATG TAG AAG GCA AA; Iso3-rev: TCA CCA GCA GGG AAT ACC TC; Iso4-fw: GCA GCA GAA GGA GGA AGA CA; Iso4-rev: AGT CCT TGA TTT CCC TGA TGG; Iso5-fw: GGC AGC AGA AGG AGG TAG AA; Iso5-rev: CCA CTT CAC TGG CTC TTC CTA. Phusion™ High-Fidelity DNA Polymerase (New England Biolabs) and the corresponding high-fidelity buffer was used for amplification. The PCR conditions were as follows: 1 cycle at 98°C for 1min, 30 cycles of denaturation, annealing and extension at, respectively, 98°C for 10 sec, 63°C (for isoform 1) or 60°C (for all other isoforms) for 30 sec, and 72°C for 30 sec, followed by one cycle at 72°C for 5 min. Primers that were published 2017 by Labbé et al. (4) were used, where the NM_001137551 had been annotated as Isoform 2, however this does not correspond to the now used APPRIS P1 transcript ENST00000863709.1 which is annotated as isoform 2 in all further analyses.

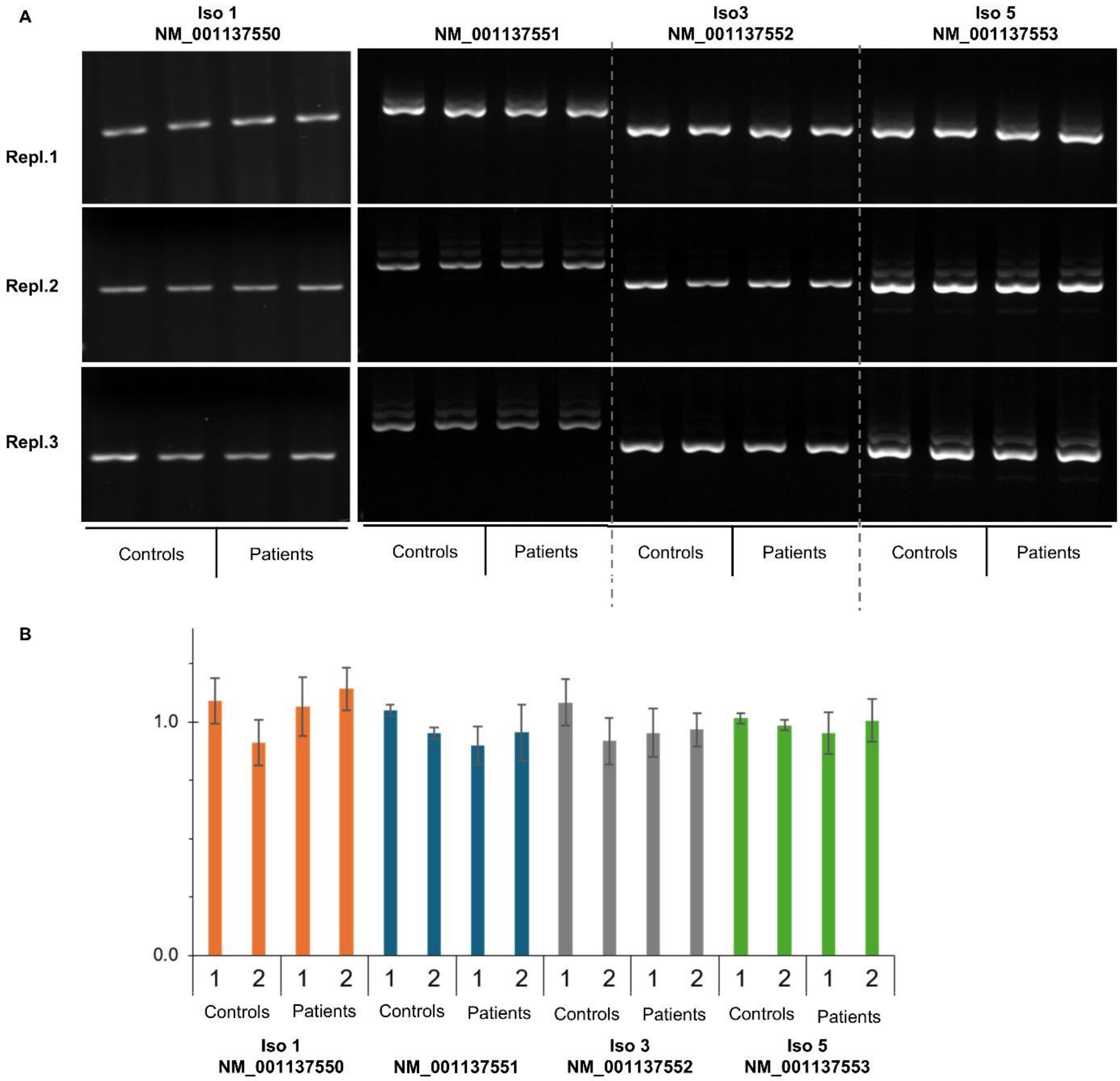

Figure S5: RT-PCR analysis in patient fibroblasts using published primers (4). Total RNA was isolated from cultured fibroblasts of affected individuals II-8 and III-12 ("patients") and unaffected individuals II-8.5 and III-13 ("controls") and analyzed by RT-PCR. Specific PCR products could be detected for four different isoforms.

8 Western Blot

To test if the observed *LRRFIP1* transcriptomic changes with increased expression of isoform 2 and decreased expression of isoforms 4 and 5 mRNAs translates to protein level, we analyzed fibroblast samples by Western blot, however there was only an antibody that detects isoforms 3-5 as a group available. No functional antibody for isoform 2 was available. For the analysis of fibroblast lysates, cells were lysed in M-PER™ Mammalian Protein Extraction Reagent (Thermo Fisher Scientific) and equal amounts of protein were processed for Western Blot. For the analysis of samples from immunoprecipitation, dilution adjusted sample volumes were used. Proteins were separated by SDS-PAGE on 4-12% Bis-Tris gels (Thermo Fisher Scientific; USA) and transferred onto nitrocellulose membranes. Membranes were blocked at room temperature for 1 hr with 5% non-fat milk in Tris-buffered saline (TBS) containing 0.05% Tween-20 before incubation with primary antibody diluted in blocking solution for 1 hr at room temperature or overnight at 4°C. Protein signals were visualized using the Odyssey™ DLx system (LI-COR Biosciences, USA). When re-hybridization with same species primary antibodies was required, membranes were stripped by 15 min incubation in 0.2 M NaOH, followed by a re-blocking for 1 hr at RT.

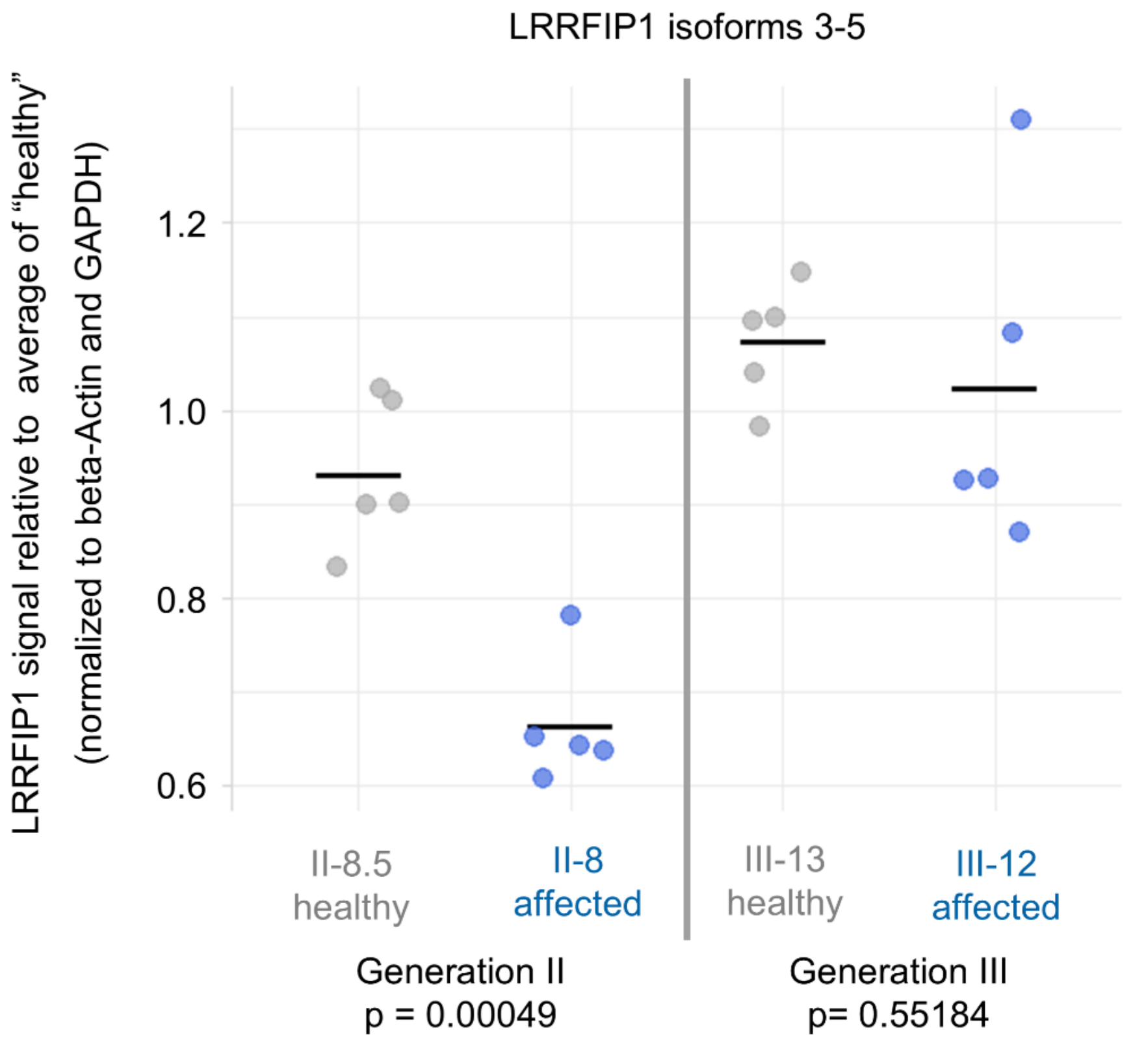

Figure S6 Western Blot of LRRFIP1 isoforms 3-5 in fibroblast samples of affected and unaffected individuals shows a lowered signal in both affected individuals, which was statistically significant in the age-matched comparison within generation II. Considering the very low mRNA transcript levels that we had previously detected for isoform 3, the protein signals are likely largely produced by isoforms 4 and 5, which is in accordance with the findings in short- and long-read RNA-seq analyses with decreased expression of isoforms 4 and 5.

9 Antibodies

The following antibodies were used: Mouse anti-FLAG antibody (Clone M2)(#F1804; Merck KGaA; Germany); rabbit anti-LRRFIP1 (for detection of isoforms 3-5)(84778-4-RR Proteintech), mouse anti-beta-Actin (CloneAC-15)(#A5441; Merck KGaA; Germany), and mouse anti-GAPDH (Clone 6C5)(#AM4300, Thermo Fisher Scientific; USA). For all antibodies, the product specification provided on the manufacturer´s website states that specificity was confirmed by Western Blot showing the presence of protein bands at the expected molecular weight in mammalian crude cell or tissue lysates. IRDye800™CW-coupled anti-rabbit IgG (#926-68072; LI-COR Biosciences, USA) or IRDye™680RD-coupled anti-mouse IgG (#926-32213; LI-COR Biosciences, USA) were used as secondary antibodies.

10 Eukaryotic cell lines

Fibroblast cultures were generated from cells outgrown from skin punch biopsies from four individuals (II-8, II-8.5, III-12, III-13). Human embryonic kidney-293 cells were purchased from DSMZ-German Collection of Microorganisms and Cell Cultures GmbH (DSMZ no.: #ACC 305). Induced pluripotent stem cells (iPSCs) used for genome editing had been derived from a 23-year-old healthy female donor as described previously (C01-Control line from (5)). All cell lines were tested negative for mycoplasma contamination. No commonly misidentified cell lines were used in this study.

Supplemental Figures S7 A-F: enlarged versions of radiographs shown in main figure 1

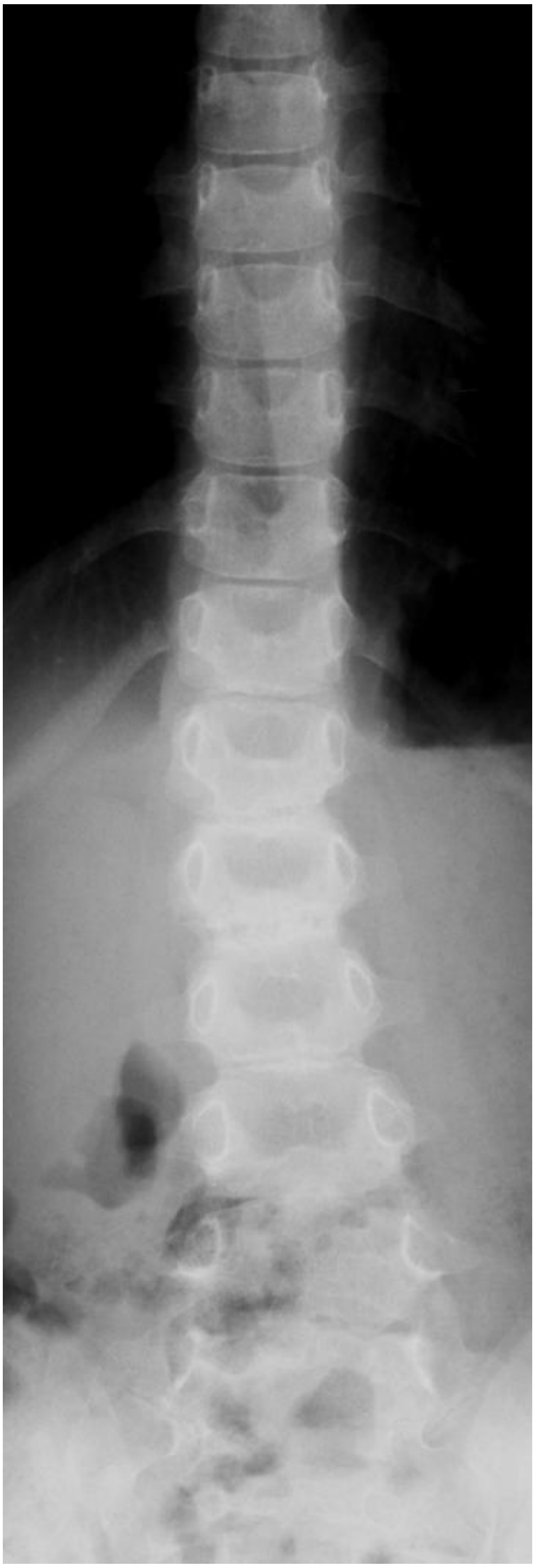

Figure S7, A: Larger view of main Figure 1, Panel A

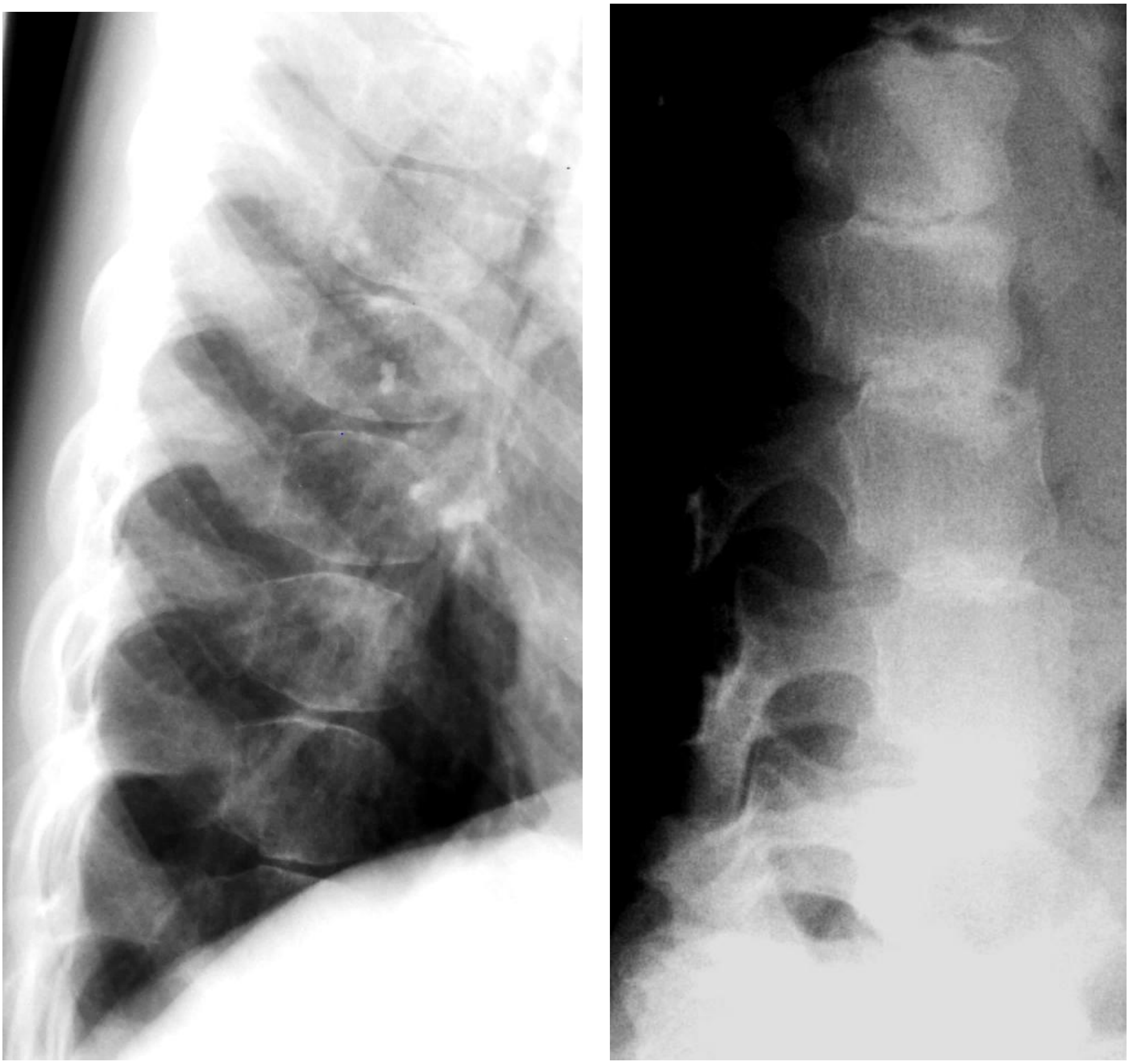

Figure S7, B: Larger view of main Figure 1, Panels B and C

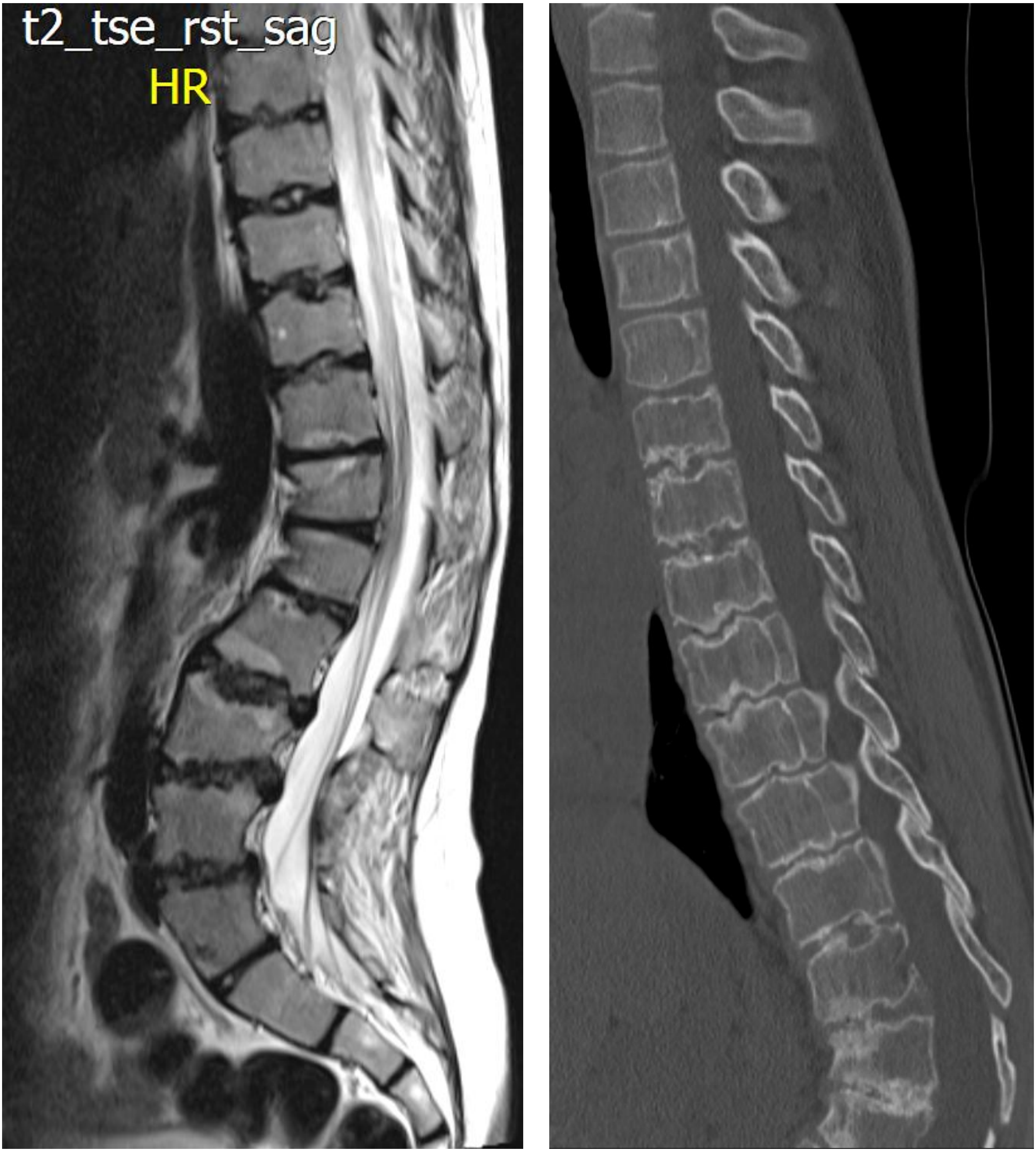

Figure S7, C: Larger view of main Figure 1, Panels D and E

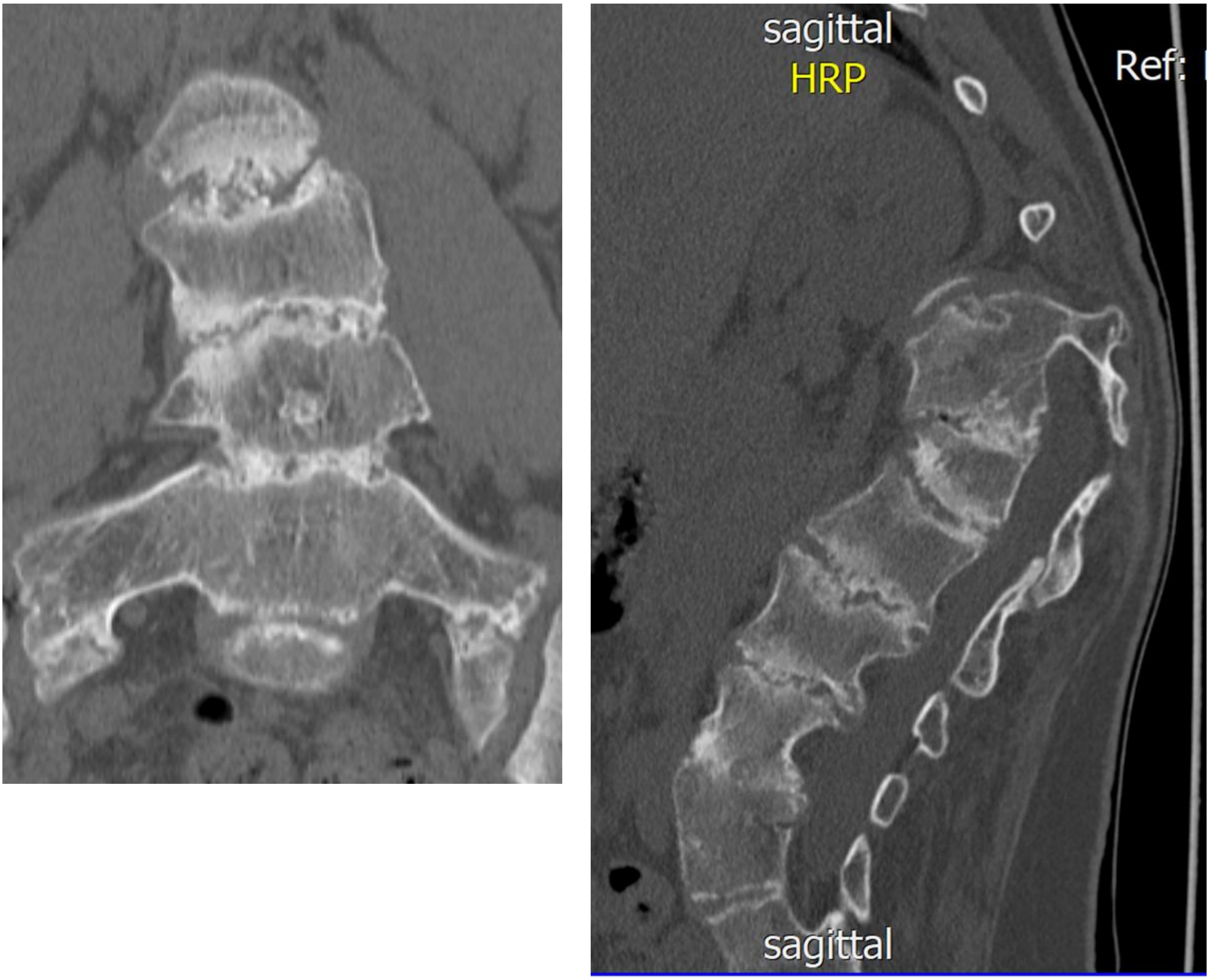

Figure S7, D: Larger view of main Figure 1, Panels F and G

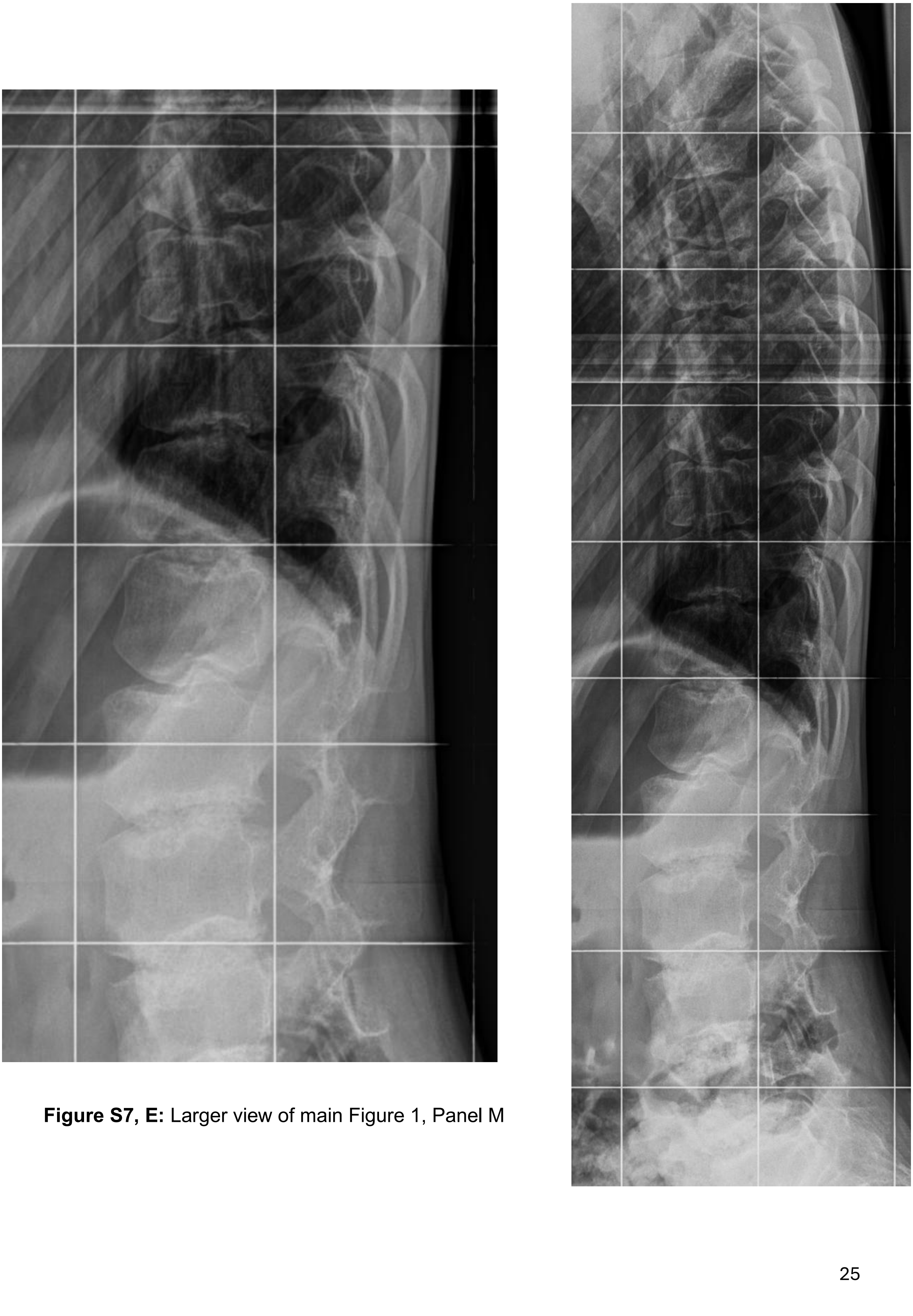

Figure S7, E: Larger view of main Figure 1, Panel M

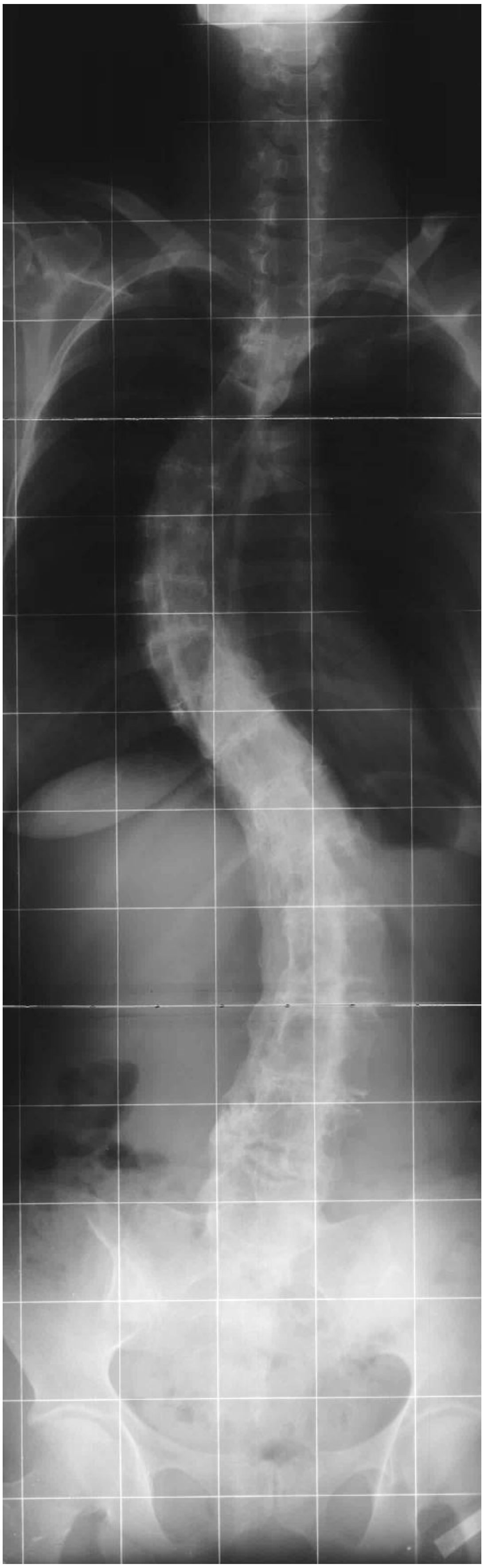

Figure S7, F: Larger view of Main Figure 1, Panel N

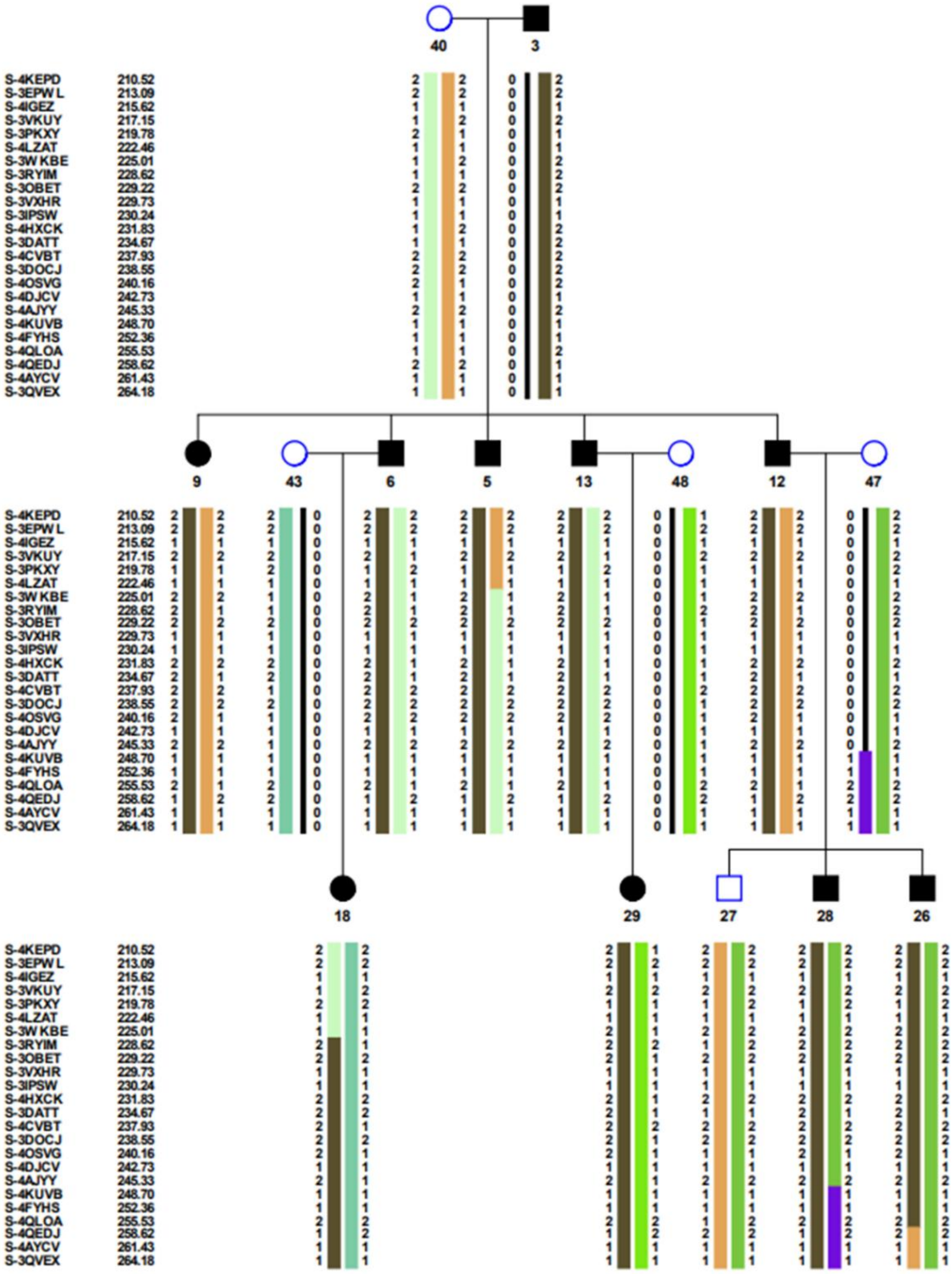

Figure S8 Haplotype analysis of the EOS candidate region on chromosome 2q35q37: The haplotypes based on SNP genotyping are visualized in HaploPainter (6), show that all affected individuals (filled in black symbols) share the same set of alleles on one chromatid (dark brown band), which is not present in any of the unaffected individuals. The haplotype analysis also shows two recombination events in affected individuals 18 and 26, which demarcate the candidate region containing the linked disease locus.

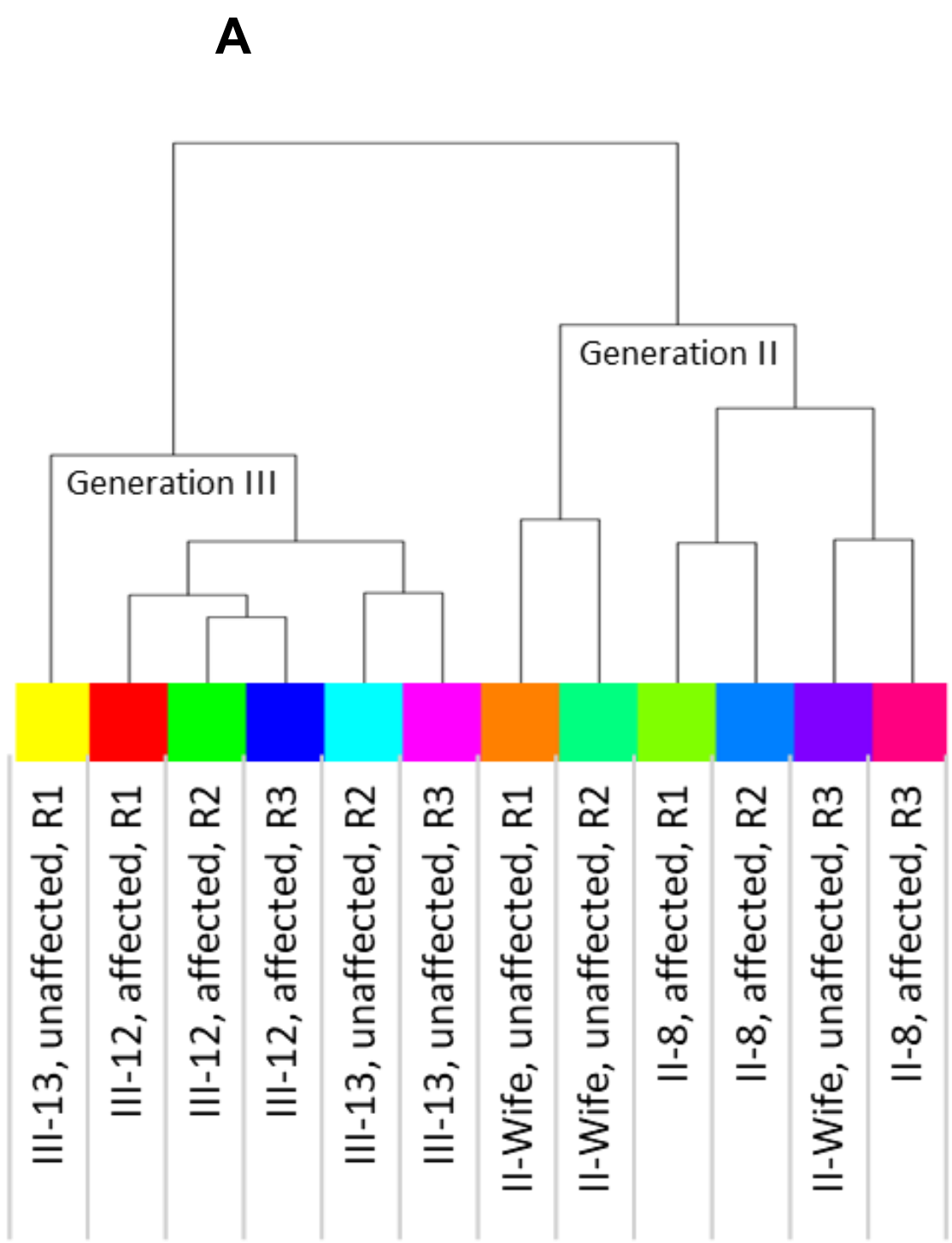

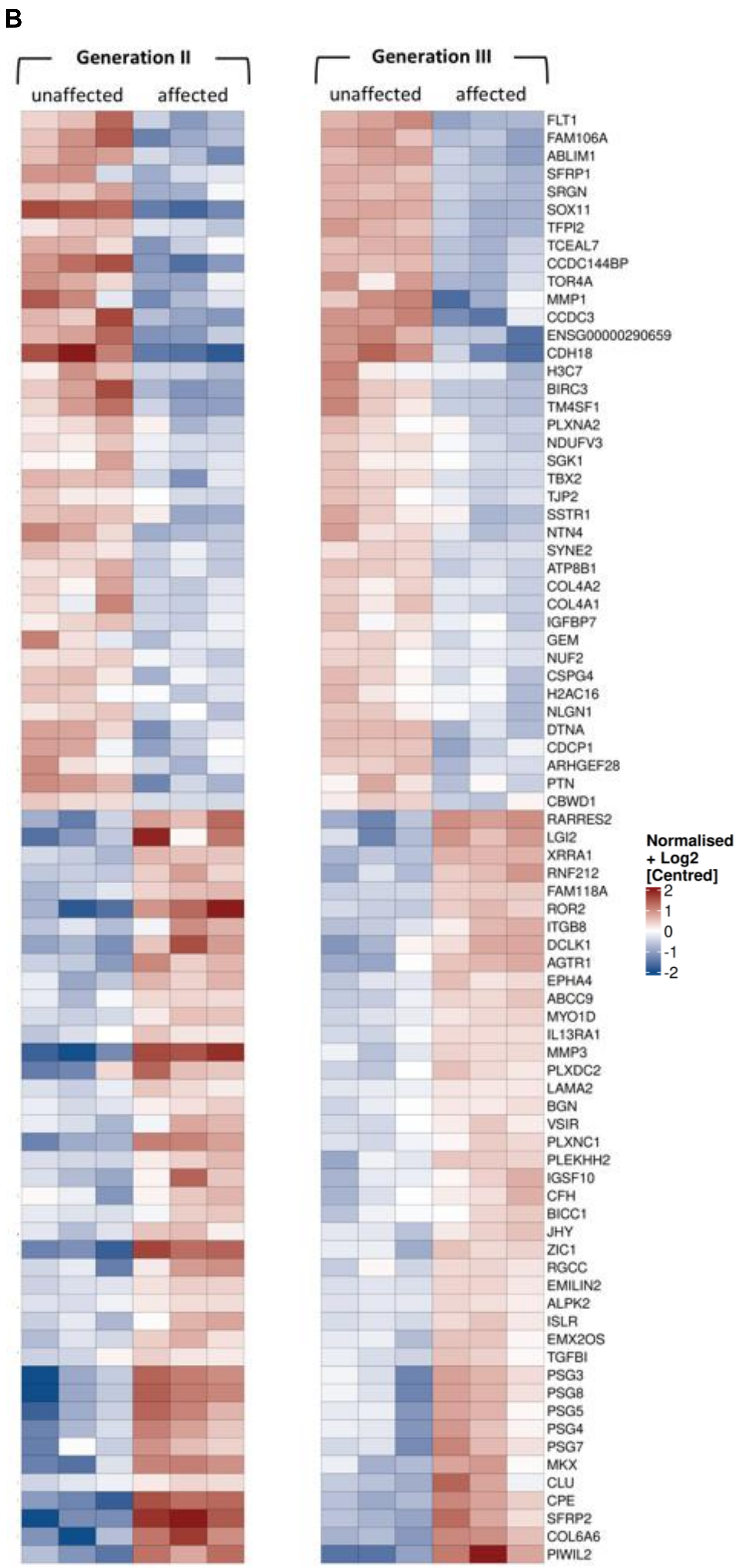

Figure S9 DEG analysis from cultured fibroblasts

(A) shows generational clustering of differential gene expression from RNA-Sequencing data from fibroblast cultures**.**

(B) shows the heatmap of differentially expressed genes shared by both generations.

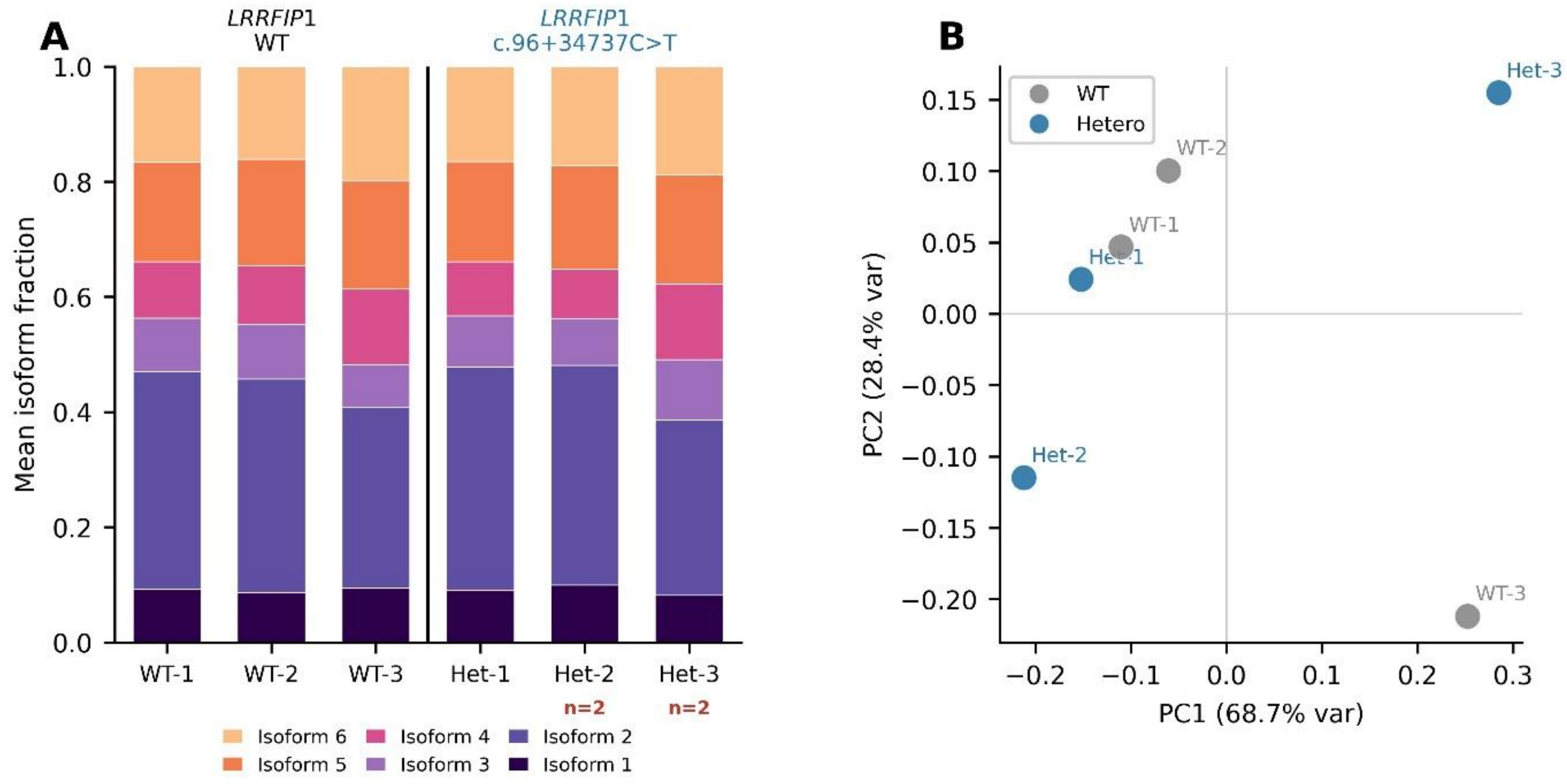

Figure S10 *LRRFIP1* isoform composition in iPSCs

(**A**) Stacked bar chart of mean *LRRFIP1* isoform fractions from short-read RNA-seq (Salmon, GENCODE v49 iso6 index) in iPSC clones derived from two individuals: three WT clones (WT-1 to WT-3) and three heterozygous clones (Het-1 to Het-3) carrying the *LRRFIP1* c.96+34737C>T variant. Each bar represents the mean across replicates (n = 3 unless noted; Het-2 and Het-3: n = 2 after exclusion of samples with total *LRRFIP1* TPM < 10). All six isoforms are meaningfully expressed (≥8%) in iPSCs. No relevant changes to isoform expression in heterozygous versus WT iPSC clones were observed (isoform 1 +0.1 percentage points (pp), isoform 2 +0.4 pp, isoform 3 -0.4 pp, isoform 4 -0.6 pp, isoform 5 +0.1 pp, isoform 6 0.0 pp).

(**B**) Aitchison PCA of isoform compositions across all six clones (all 6 isoforms, CLR-transformed). PC1 explains 68.7% of variance. Aitchison distance between WT and heterozygous centroids = 0.08, indicating no separation by genotype in iPSCs.

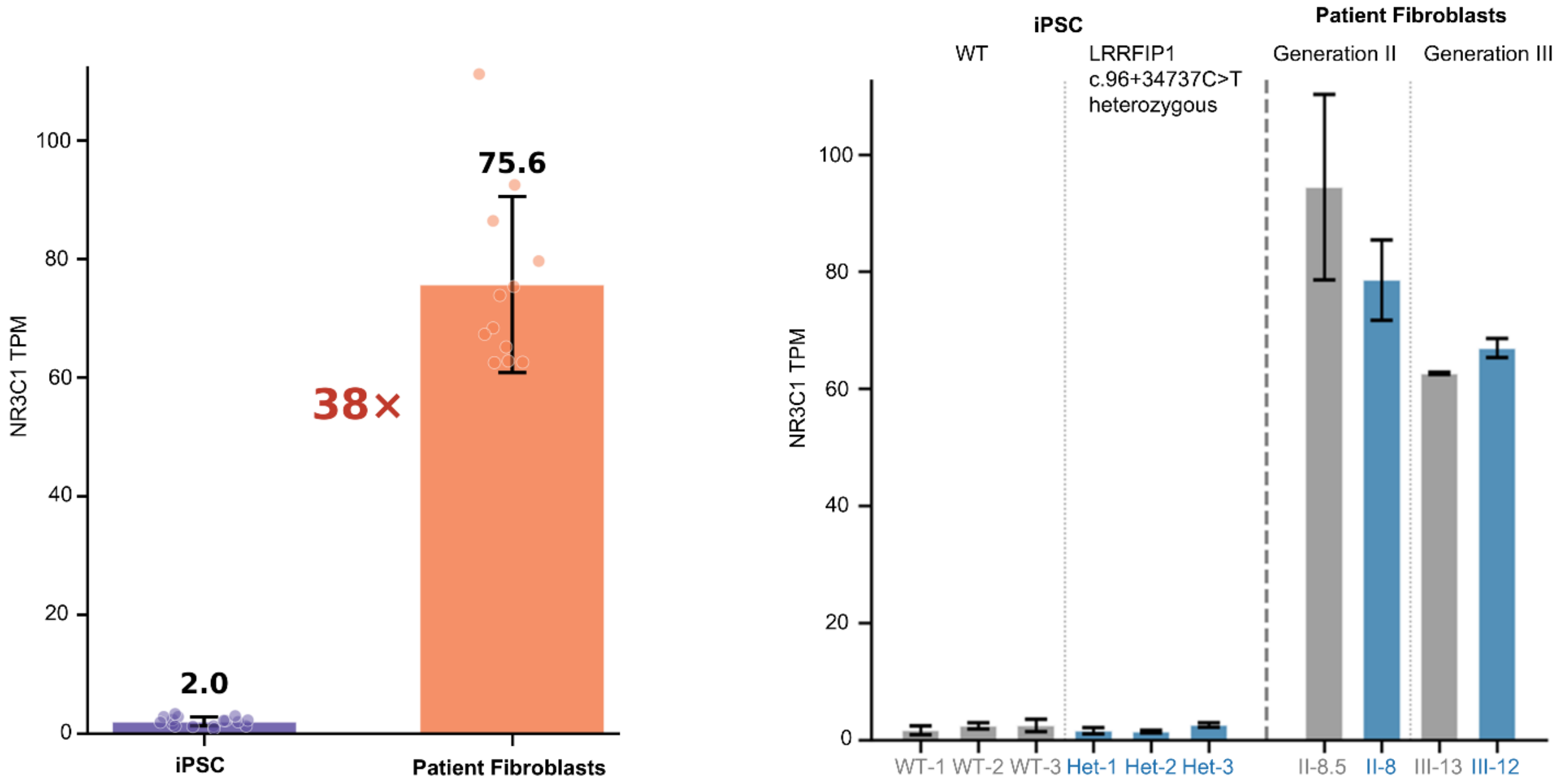

Figure S11 *NR3C1* expression in iPSCs and fibroblasts

Overall *NR3C1* expression (TPM, Salmon, GENCODE v49 iso6 index) comparing iPSCs (mean 2.0 TPM) and fibroblasts (mean 75.6 TPM), representing a 38-fold difference. Individual *NR3C1* expression analysis from fibroblasts shows higher expression of *NR3C1* in the older affected individual II-8 (mean TPM 78.56) compared to the younger affected individual III-12 (mean TPM 66.94). Bars show the mean of individual/clones; error bars show standard deviation across all replicates. Individual data points are overlaid.

Table S2: Isoform expression changes compared to age-matched wildtype controls in relation to *NR3C1* expression

Sample	Genotype	NR3C1 expression (mean TPM)	Expression change Isoform 2 short-read RNA-seq (Δ percentage points)	Expression change Isoform 2 long read RNA-seq (Δ percentage points)	Expression change Isoform 4 short read RNA-seq (Δ percentage points)	Expression change Isoform 4 long read RNA-seq (Δ percentage points)
Fibroblasts individual II-8	*LRRFIP1* c.96+34737C>T het	78.56	+4.9	+8.7	-2.2	-8.1
Fibroblasts individual III-12	*LRRFIP1* c.96+34737C>T het	66.94	+1.9	+4.7	-3.5	-3.5
iPSCs	*LRRFIP1* c.96+34737C>T het	2.0	+0.4	n/a	-0.6	n/a

Table S3: FDR values for expression differences of the six genes within the linked region

gene_id	gene_name	FDR value generation II	FDR value generation III
ENSG00000163359	*COL6A3*	0.972	0.515
ENSG00000115648	*MLPH*	0.797	0.326
ENSG00000071677	*PRLH*	Expression too low	Expression too low
ENSG00000124839	*RAB17*	0.748	Expression too low
ENSG00000124831	*LRRFIP1*	0.794	0.995
ENSG00000177483	*RBM44*	0.992	0.913

Table S4: Overview of pairwise comparison of isoform expression changes in affected individuals compared with age-matched unaffected individuals in both short- and long-read RNA-seq data

Pairwise comparison affected vs unaffected isoform expression (Δ percentage points)				
sample	fibroblasts	fibroblasts	fibroblasts	fibroblasts
RNA-seq	short-read	long-read	short-read	long-read
generation	generation II	generation II	generation III	generation III
Isoform 1	low expression	no expression	low expression	no expression
Isoform 2	+4.9	+8.7	+1.9	+4.7
Isoform 3	+2.3	-0.6	0	0
Isoform 4	-2.2	-8.1	-3.5	-3.5
Isoform 5	-8.5	+2.1	-4.3	-7.7
Isoform 6	+2.9	-0.4	+5.7	+2.8

Table S5: FDR values of the differentially expressed genes shown in main Figure 4 (G and H)

gene_id	gene_name	FDR value generation II	FDR value generation III
ENSG00000169071	*ROR2*	5.22E-05	2.51E-03
ENSG00000104332	*SFRP1*	1.34E-02	3.65E-05
ENSG00000145423	*SFRP2*	9.55E-08	2.77E-07
ENSG00000023445	*BIRC3*	1.52E-04	1.07E-02
ENSG00000151468	*CCDC3*	6.07E-03	1.24E-05
ENSG00000149968	*MMP3*	1.05E-11	1.28E-03

Table S6: Top 10 most enriched Gene Ontology Biological Processes using the significantly differentially expressed genes from RNA sequencing data from individuals with *LRRFIP1*-EOS compared to unaffected generation-matched relatives

**GO biological process**	**DEG Count***	**Expected Gene Count****	**Fold Enrichment**	**Raw P-value**	**False Discovery Rate (FDR)**
Wnt signaling pathway involved in somitogenesis (GO:0090244)	2	0.01	> 100	4.24E-05	1.90E-02
Regulation of midbrain dopaminergic neuron differentiation (GO:1904956)	2	0.01	> 100	4.24E-05	1.84E-02
Protein localization to secretory granule (GO:0033366)	2	0.02	> 100	8.47E-05	3.20E-02
Regulation of establishment of planar polarity (GO:0090175)	3	0.03	> 100	1.81E-06	2.23E-03
Positive regulation of fat cell differentiation (GO:0045600)	4	0.26	15.3	1.37E-04	4.39E-02
Female pregnancy (GO:0007565)	7	0.67	10.38	5.07E-06	4.99E-03
Multi-organism reproductive process (GO:0044703)	7	0.75	9.33	1.02E-05	7.15E-03
Multi-multicellular organism process (GO:0044706)	7	0.79	8.88	1.40E-05	7.95E-03
Extracellular matrix organization (GO:0030198)	8	1.03	7.79	8.62E-06	7.48E-03
Extracellular structure organization (GO:0043062)	8	1.03	7.76	8.85E-06	7.26E-03

*DEG Count refers to the number of differentially expressed genes mapping to the respective annotation data category

**Expected Gene Count refers to the number of genes expected for the respective annotation data category based on the reference list.

Table S7: Top 10 most enriched Reactome Pathways using the significantly differentially expressed genes from RNA sequencing data from individuals with *LRRFIP1*-EOS compared to unaffected generation-matched relatives

**Reactome Pathway**	**DEG Count***	**Expected Gene Count****	**Fold Enrichment**	**Raw P-value**	**False Discovery Rate (FDR)**
**Laminin interactions (R-HSA-3000157)**	3	0.11	26.38	1.98E-04	4.31E-02
**Collagen degradation (R-HSA-1442490)**	5	0.24	20.61	4.40E-06	5.76E-03
**Non-integrin membrane-ECM interactions (R-HSA-3000171)**	4	0.22	17.89	7.42E-05	2.78E-02
**Assembly of collagen fibrils and other multimeric structures (R-HSA-2022090)**	4	0.23	17.59	7.92E-05	2.59E-02
**ECM proteoglycans (R-HSA-3000178)**	5	0.29	17.36	1.03E-05	5.39E-03
Platelet degranulation (R-HSA-114608)	5	0.48	10.39	1.22E-04	3.54E-02
Response to elevated platelet cytosolic Ca2+ (R-HSA-76005)	5	0.5	9.99	1.46E-04	3.82E-02
**Degradation of the extracellular matrix (R-HSA-1474228)**	5	0.53	9.42	1.92E-04	4.58E-02
Cell surface interactions at the vascular wall (R-HSA-202733)	7	0.75	9.38	9.85E-06	6.45E-03
**Extracellular matrix organization (R-HSA-1474244)**	10	1.13	8.85	1.84E-07	4.82E-04

Bold text indicates pathways that are part of Extracellular Matrix Organization

*DEG Count refers to the number of differentially expressed genes mapping to the respective annotation data category

**Expected Gene Count refers to the number of genes expected for the respective annotation data category based on the reference list.

Table S8: Scoliotic Cobb angle, Kyphotic Index and Hyperlordosis measurements of Lrrfip1-knock out mice

Specimen ID IMPC	randomly generated mouse ID (blinded)		scoliotic cobb angle 1st measurement	scoliotic cobb angle 2nd measurement	**mean cobb angle**	kyphosis index 1st measurement	kyphosis index 2nd measurement	**mean kyphotic Index***	**affected by hyperlordosis (1= yes, 0=no)**
280730	17	Lrrfip1$^{tm1.1(KOMP)Wtsi}$ Het female 13 weeks	15	16	**15.5**	2.82	2.55	**2.685**	**1**
285970	63	Lrrfip1$^{tm1.1(KOMP)Wtsi}$ Hom female 13 weeks	22	20	**21**	3.14	3.3	**3.22**	**1**
267807	9	Lrrfip1$^{tm1.1(KOMP)Wtsi}$ Het female 14 weeks	12	14	**13**	3.11	2.9	**3.005**	**1**
267809	41	Lrrfip1$^{tm1.1(KOMP)Wtsi}$ Het female 14 weeks	7	9	**8**	2.73	2.77	**2.75**	**1**
267808	39	Lrrfip1$^{tm1.1(KOMP)Wtsi}$ Het female 14 weeks	6	5	**5.5**	2.92	2.88	**2.9**	**1**
267806	13	Lrrfip1$^{tm1.1(KOMP)Wtsi}$ Het female 14 weeks	9	9	**9**	3.14	3	**3.07**	**1**
278406	57	Lrrfip1$^{tm1.1(KOMP)Wtsi}$ Hom female 14 weeks	13	14	**13.5**	2.8	2.8	**2.8**	**1**
263551	5	Lrrfip1$^{tm1.1(KOMP)Wtsi}$ Hom female 14 weeks	13	12	**12.5**	2.52	2.48	**2.5**	**1**
280727	23	Lrrfip1$^{tm1.1(KOMP)Wtsi}$ Het male 13 weeks	5	2	**3.5**	2.96	2.88	**2.92**	**0**
280726	62	Lrrfip1$^{tm1.1(KOMP)Wtsi}$ Het male 13 weeks	3	0	**1.5**	2.75	2.79	**2.77**	**1**
285965	34	Lrrfip1$^{tm1.1(KOMP)Wtsi}$ Hom male 13 weeks	12	12	**12**	3.05	2.82	**2.935**	**1**
278188	16	Lrrfip1$^{tm1.1(KOMP)Wtsi}$ Het male 14 weeks	5	2	**3.5**	2.95	2.75	**2.85**	**1**
278189	44	Lrrfip1$^{tm1.1(KOMP)Wtsi}$ Het male 14 weeks	3	3	**3**	3.29	3.08	**3.185**	**0**
280670	3	Lrrfip1$^{tm1.1(KOMP)Wtsi}$ Het male 14 weeks	13	13	**13**	3.04	3	**3.02**	**1**
280669	21	Lrrfip1$^{tm1.1(KOMP)Wtsi}$ Het male 14 weeks	5	8	**6.5**	2.69	2.91	**2.8**	**1**
263545	20	Lrrfip1$^{tm1.1(KOMP)Wtsi}$ Hom male 14 weeks	15	15	**15**	2.95	2.89	**2.92**	**1**
280671	31	Lrrfip1$^{tm1.1(KOMP)Wtsi}$ Hom male 14 weeks	0	0	**0**	2.88	2.88	**2.88**	**1**
287446	53	Lrrfip1$^{tm1.1(KOMP)Wtsi}$ Hom male 14 weeks	8	6	**7**	3.29	3.45	**3.37**	**1**

* lower kyphotic index represents more severe kyphosis

Table S9: Scoliotic Cobb angle, Kyphotic Index and Hyperlordosis measurements of wildtype control mice

Specimen ID IMPC	randomly generated mouse ID (blinded)		Scoliotic cobb angle 1st measurement	Scoliotic Cobb angle 2nd measurement	**mean scoliotic Cobb angle**	kyphosis index 1st measurement	kyphosis index 2nd measurement	**mean kyphotic index*** * lower kyphotic index represents more severe kyphosis	**presence of hyperlordosis (1= yes, 0=no)**
36068	81	WT Female 13 weeks	8	7	**7.5**	3.14	3.13	**3.135**	**0**
50988	67	WT Female 13 weeks	6	9	**7.5**	2.96	2.8	**2.88**	**0**
47806	56	WT Female 13 weeks	5	4	**4.5**	3	3	**3**	**0**
42418	2	WT Female 13 weeks	3	0	**1.5**	3.04	2.92	**2.98**	**0**
53745	22	WT Female 13 weeks	0	0	**0**	3.09	3.24	**3.165**	**0**
50989	35	WT Female 13 weeks	5	4	**4.5**	3.2	3.2	**3.2**	**0**
37975	25	WT Female 13 weeks	12	9	**10.5**	3.17	3.09	**3.13**	**0**
50696	54	WT Female 13 weeks	6	3	**4.5**	2.85	2.85	**2.85**	**0**
55489	59	WT Female 13 weeks	3	0	**1.5**	3.08	2.92	**3**	**0**
47805	77	WT Female 13 weeks	6	8	**7**	**n/a**	n/a	**no lateral xray**	**n/a**
36069	46	WT Female 13 weeks	8	9	**8.5**	2.8	2.8	**2.8**	**0**
45675	10	WT Female 14 weeks	15	14	**14.5**	3.04	2.8	**2.92**	**0**
50497	70	WT Female 14 weeks	7	8	**7.5**	3.18	3.05	**3.115**	**0**
34368	29	WT Female 14 weeks	10	11	**10.5**	2.71	2.7	**2.705**	**0**
38695	45	WT Female 14 weeks	8	7	**7.5**	2.76	2.65	**2.705**	**0**
50155	4	WT Female 14 weeks	11	11	**11**	2.86	2.78	**2.82**	**0**
38696	30	WT Female 14 weeks	4	4	**4**	2.52	2.5	**2.51**	**0**
44290	18	WT Female 14 weeks	3	4	**3.5**	3.39	3.21	**3.3**	**0**
46779	7	WT Female 14 weeks	3	0	**1.5**	3.4	3.19	**3.295**	**0**
50496	38	WT Female 14 weeks	4	7	**5.5**	**n/a**	n/a	**no lateral xray**	**n/a**
46780	51	WT Female 14 weeks	7	4	**5.5**	3	2.95	**2.975**	**0**

Table S9 (continued)

Specimen ID IMPC	randomly generated mouse ID (blinded)		scoliotic cobb angle 1st measurement	scoliotic cobb angle 2nd measurement	**mean cobb angle**	kyphosis index 1st measurement	kyphosis index 2nd measurement	**mean kyphotic index*** * lower kyphotic index represents more severe kyphosis	**affected by hyperlordosis (1= yes, 0=no)**
50699	49	WT Female 14 weeks	3	0	**1.5**	3.09	3	**3.045**	**0**
42987	64	WT Female 14 weeks	2	5	**3.5**	3.4	3.35	**3.375**	**0**
37985	66	WT Female 14 weeks	8	9	**8.5**	3.14	3.05	**3.095**	**0**
42984	55	WT Female 14 weeks	6	6	**6**	3.47	3.37	**3.42**	**0**
36394	27	WT Male 13 weeks	7	5	**6**	3.04	3.23	**3.135**	**0**
48096	61	WT Male 13 weeks	7	6	**6.5**	3.6	3.27	**3.435**	**0**
47800	14	WT Male 13 weeks	0	0	**0**	3.38	3.43	**3.405**	**0**
36071	12	WT Male 13 weeks	3	0	**1.5**	3.68	3.63	**3.655**	**0**
47801	50	WT Male 13 weeks	3	0	**1.5**	**n/a**	n/a	**no lateral xray**	**n/a**
53742	28	WT Male 13 weeks	7	6	**6.5**	3	3.04	**3.02**	**0**
50984	15	WT Male 13 weeks	0	0	**0**	3.5	3.35	**3.425**	**0**
36070	37	WT Male 14 weeks	11	14	**12.5**	3.62	3.57	**3.595**	**0**
34137	33	WT Male 14 weeks	11	8	**9.5**	3.67	3.61	**3.64**	**0**
50495	42	WT Male 14 weeks	12	9	**10.5**	3.09	3	**3.045**	**0**
38693	36	WT Male 14 weeks	5	2	**3.5**	3.25	3.08	**3.165**	**0**
33357	76	WT Male 14 weeks	0	0	**0**	3.27	3.04	**3.155**	**0**
38694	32	WT Male 14 weeks	0	0	**0**	3.13	3.35	**3.24**	**0**
36476	26	WT Male 14 weeks	0	0	**0**	3	3	**3**	**0**
44288	43	WT Male 14 weeks	6	4	**5**	3.14	3.07	**3.105**	**0**
30460	58	WT Male 14 weeks	5	6	**5.5**	3.48	3.23	**3.355**	**0**
50694	60	WT Male 14 weeks	0	0	**0**	3.59	3.35	**3.47**	**0**
44289	40	WT Male 14 weeks	0	0	**0**	3.13	2.92	**3.025**	**0**
45669	1	WT Male 14 weeks	2	0	**1**	3.52	3.38	**3.45**	**0**
50494	11	WT Male 14 weeks	3	0	**1.5**	3.5	3.65	**3.575**	**0**

Supplemental References

1. Wakeling E, et al. Missense substitutions at a conserved 14-3-3 binding site in hdac4 cause a novel intellectual disability syndrome. *HGG Adv*. 2021;2(1):100015.
2. Groza T, et al. The international mouse phenotyping consortium: Comprehensive knockout phenotyping underpinning the study of human disease. *Nucleic Acids Res*. 2023;51(D1):D1038–D1045.
3. Waterhouse AM, et al. Jalview version 2--a multiple sequence alignment editor and analysis workbench. *Bioinformatics*. 2009;25(9):1189–91.
4. Labbe P, et al. The alternatively spliced lrrfip1 isoform-1 is a key regulator of the wnt/beta-catenin transcription pathway. *Biochim Biophys Acta Mol Cell Res*. 2017;1864(7):1142–1152.
5. Asadollahi R, et al. Pathogenic scn2a variants cause early-stage dysfunction in patient-derived neurons. *Hum Mol Genet*. 2023;32(13):2192–2204.
6. Thiele H, Nurnberg P. Haplopainter: A tool for drawing pedigrees with complex haplotypes. *Bioinformatics*. 2005;21(8):1730–2.
