# Supplementary material for: Rare LRRFIP1 variants contribute to the pathophysiology of severe early-onset scoliosis and common scoliotic phenotypes
Source: J Clin Invest. 2026 Sep 29;136(22):e202751. doi: 10.1172/JCI202751 (PMC13769427; doi:10.1172/JCI202751)

Full unedited gel for Figure 3D
Run 1
Electromobility shift assay (EMSA) of *LRRFIP1* DNA oligonucleotide probes with nuclear extracts.

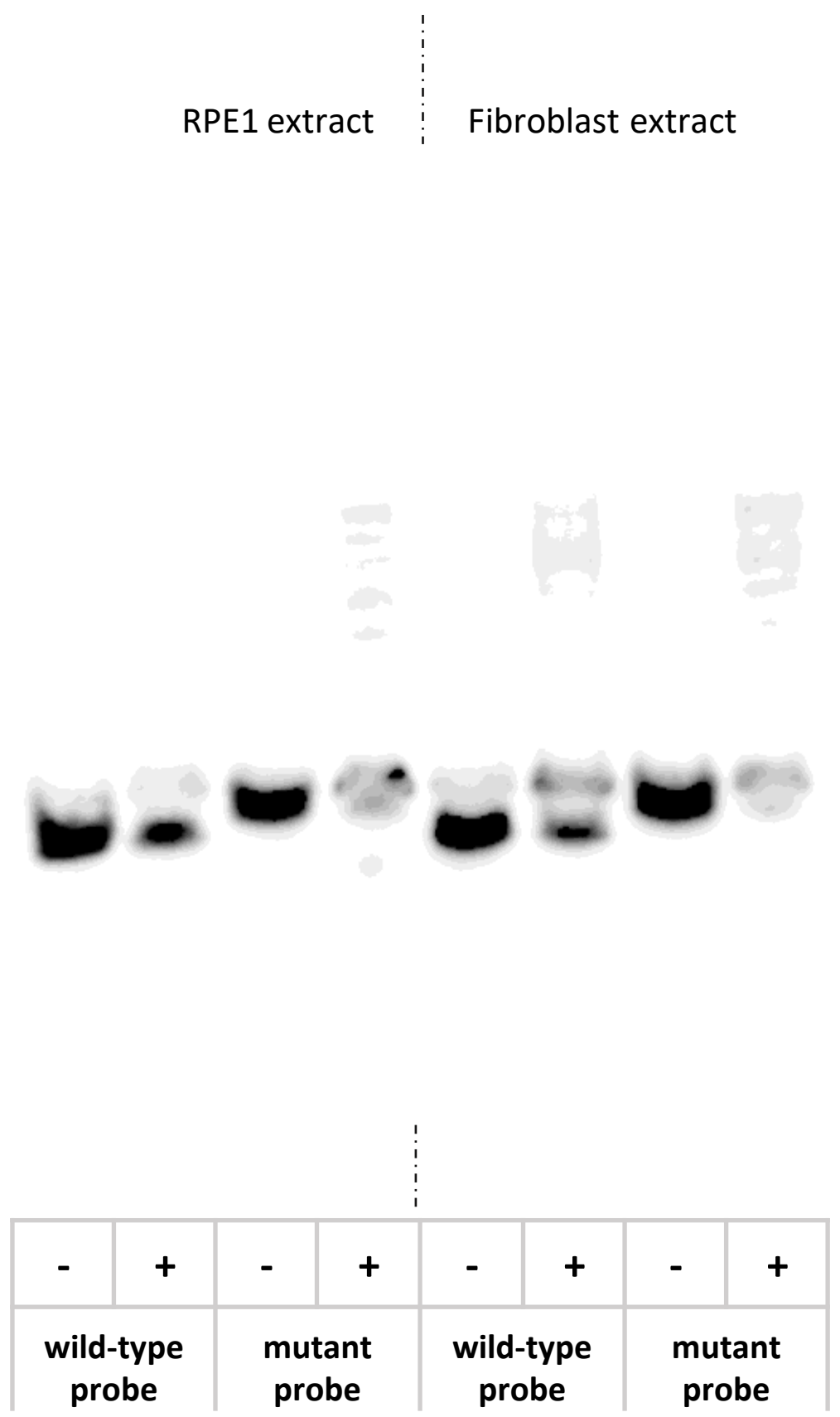

Full unedited gel for Figure 3D
Run 2
Electromobility shift assay (EMSA) of LRRFIP1 DNA oligonucleotide probes with nuclear extracts.

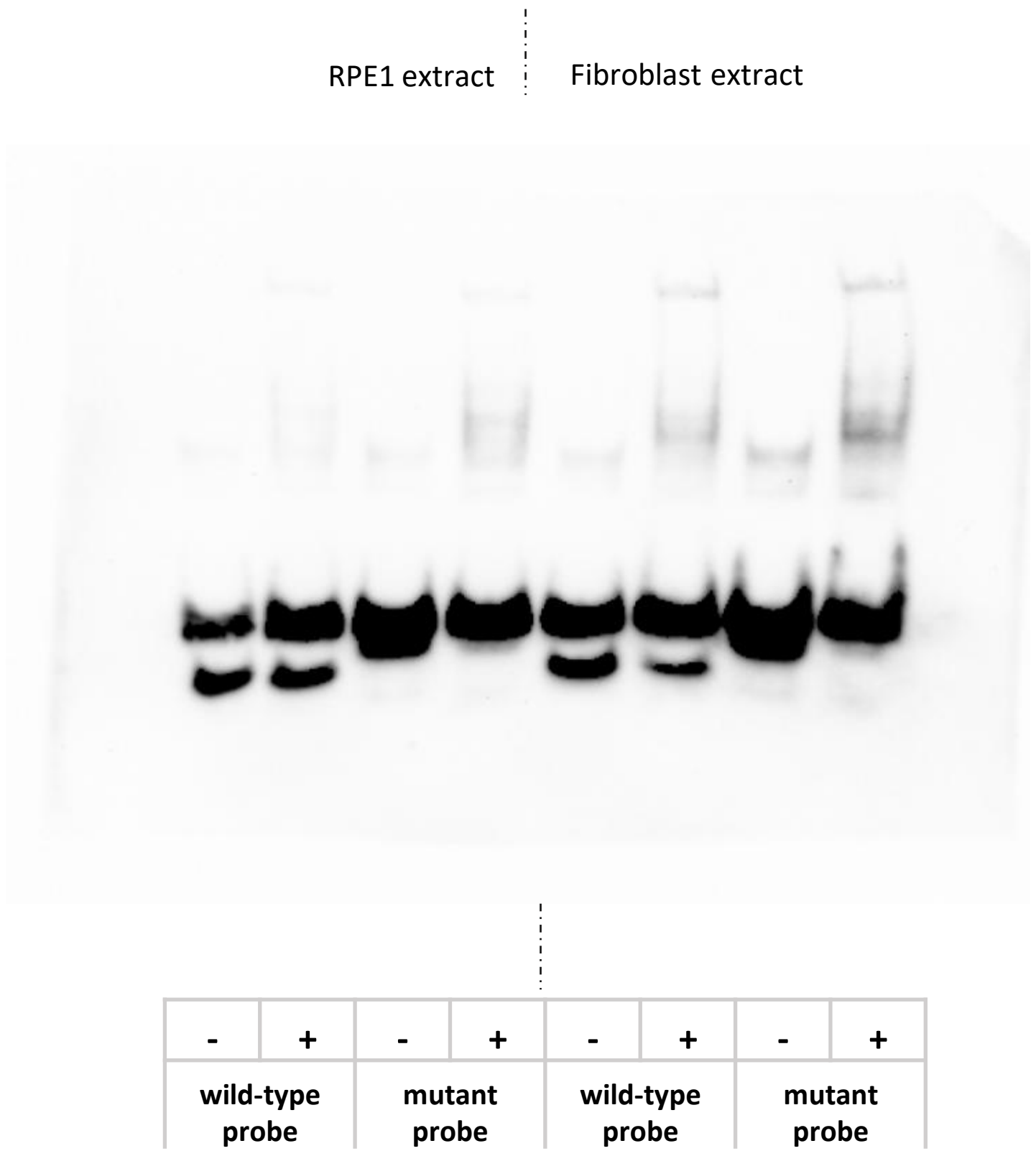

Full unedited gel for Figure 3D
Run 3
Electromobility shift assay (EMSA) of LRRFIP1 DNA oligonucleotide probes with nuclear extracts.

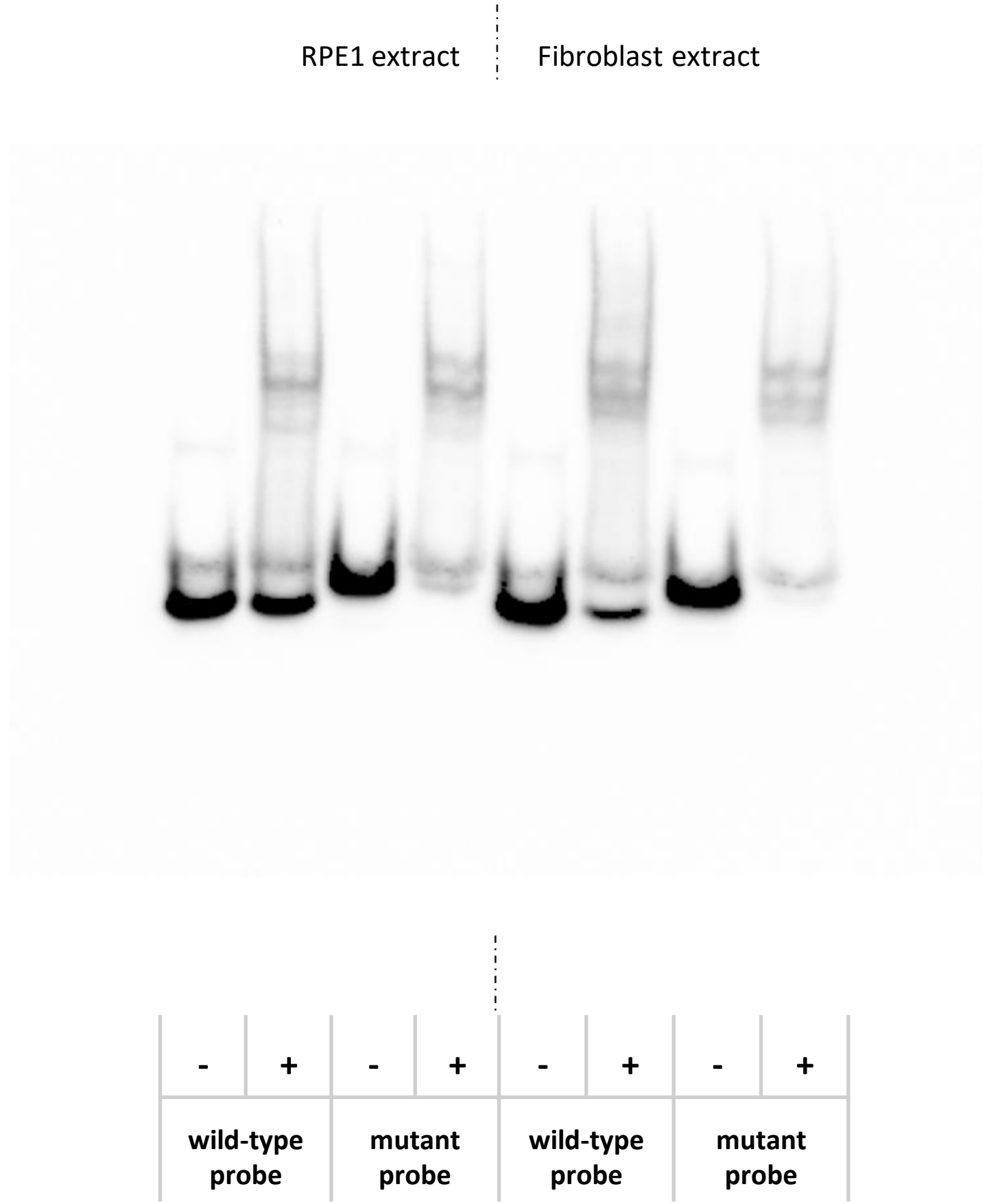

Full unedited gel for Figure S1, A

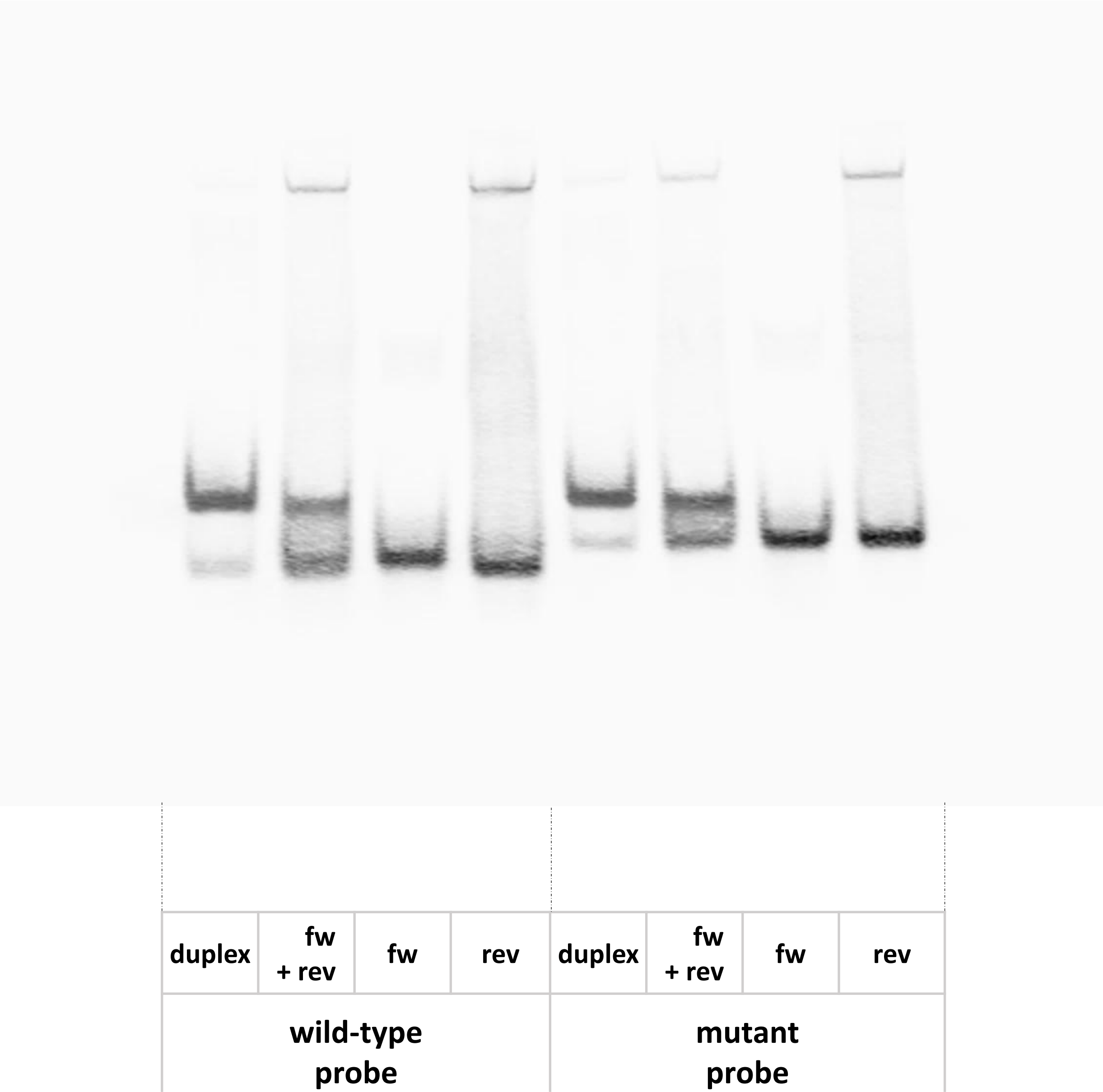

Full unedited gel for Figure S1, B

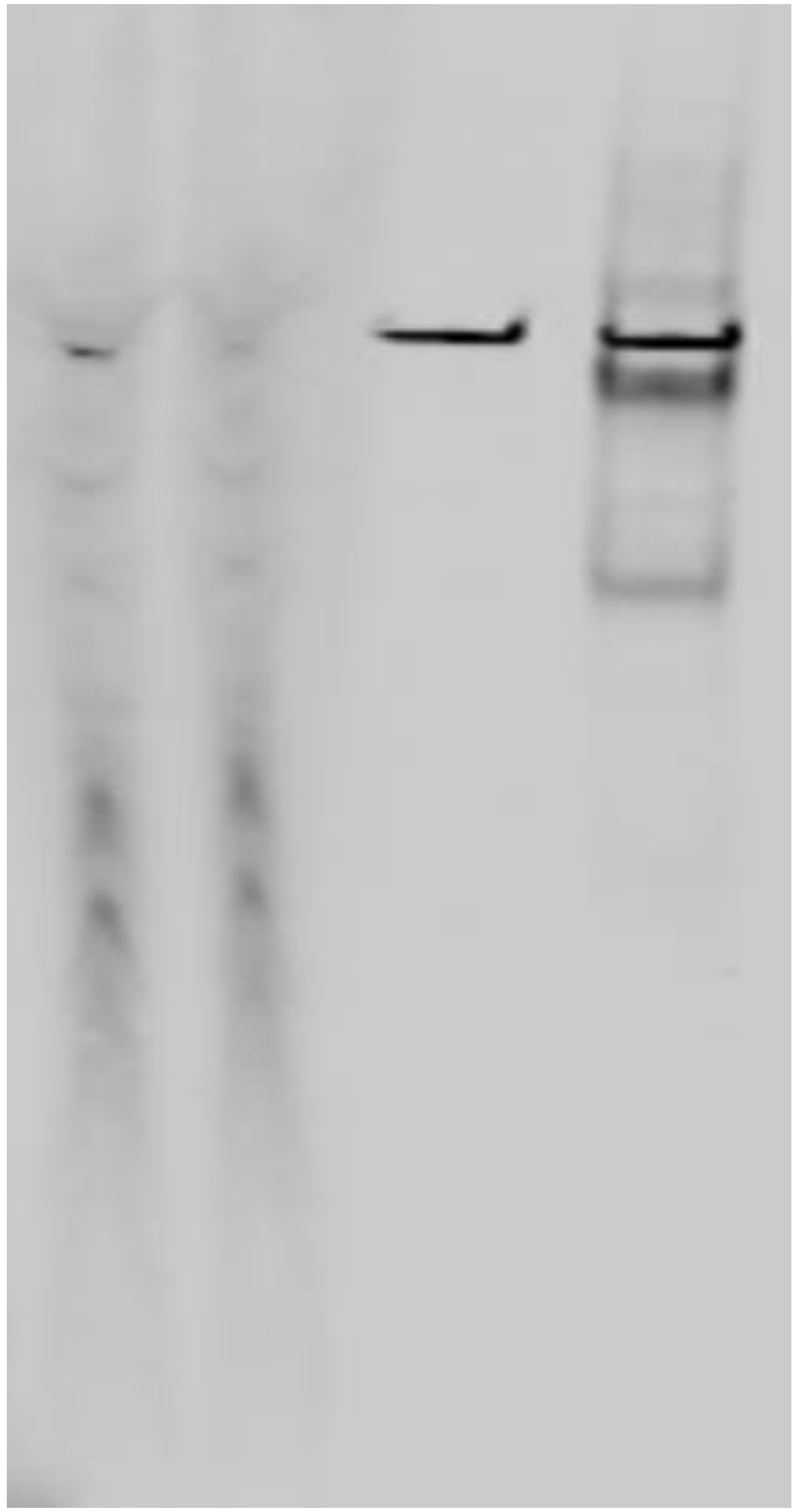

Full unedited gel for Figure S1, C (run 1)

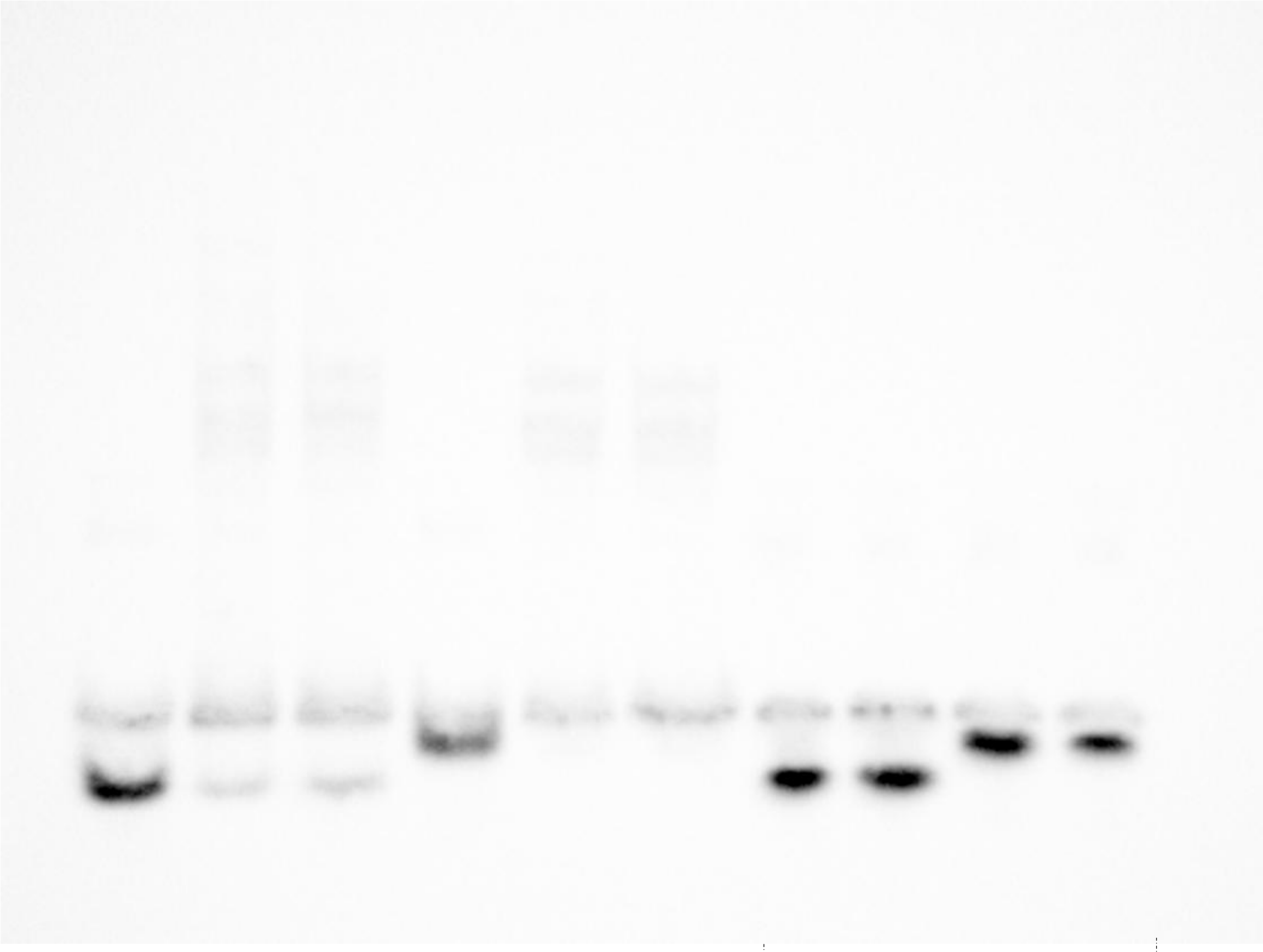

duplex

single-strand

Run 1

NR3C1-FLAG	-	+	-	+
	wild-type probe		mutant probe	

Full unedited gel for Figure S1, C (run 2)

duplex

single-strand

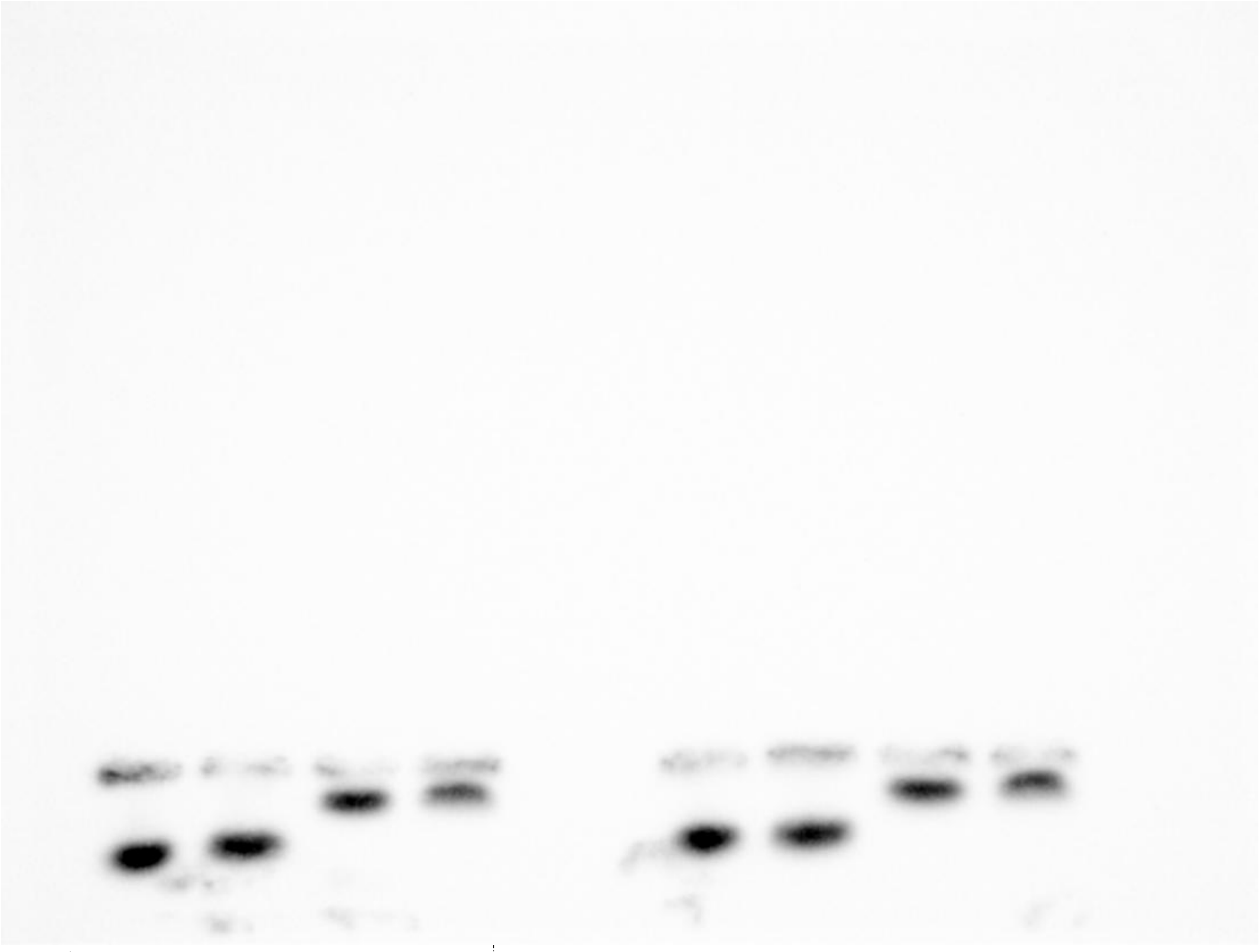

Run 2

**NR3C1-FLAG**	**-**	**+**	**-**	**+**
	**wild-type probe**		**mutant probe**	

Full unedited gel for Figure S1, C (run 3)

duplex

single-strand

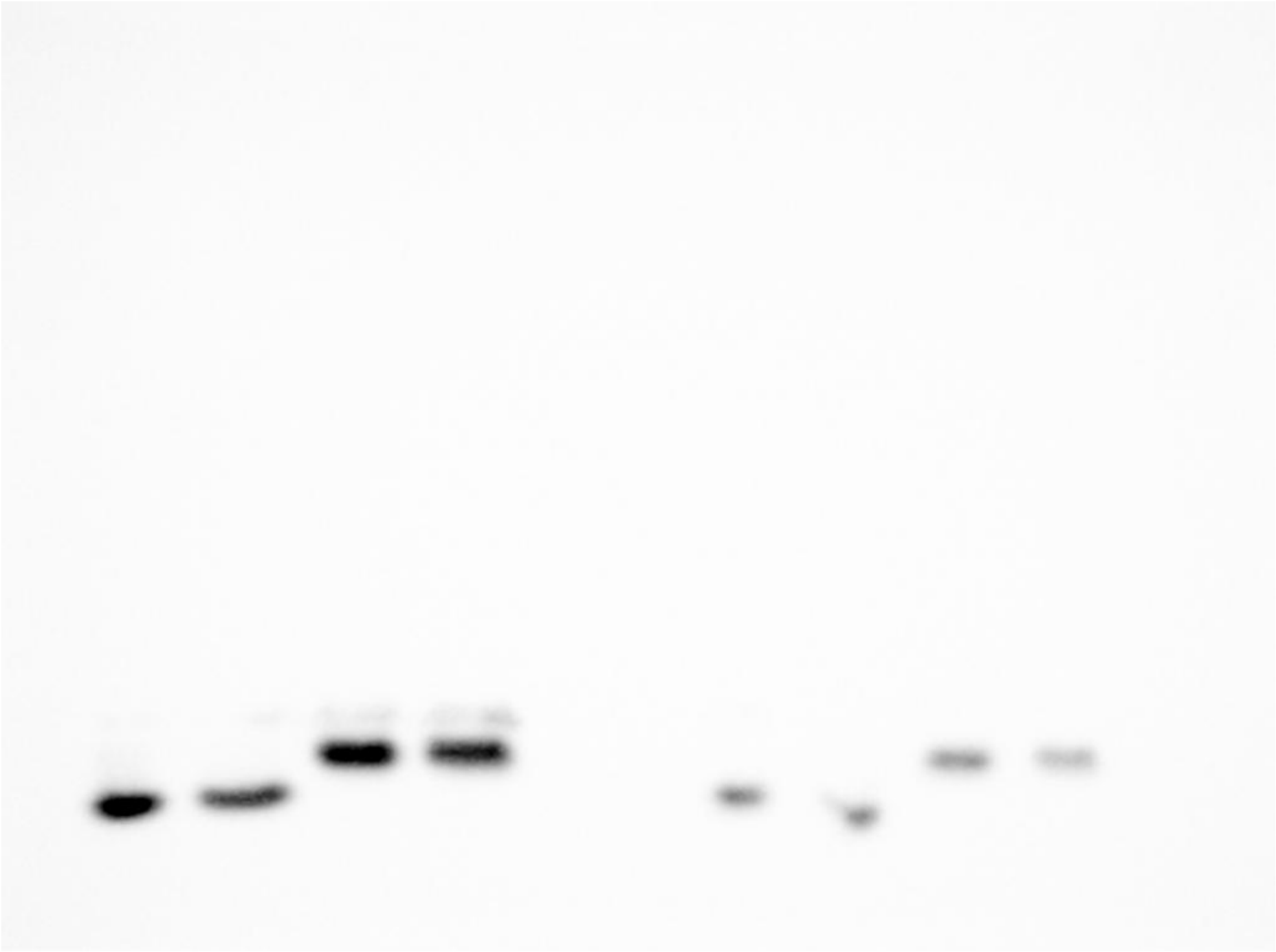

Run 3

NR3C1-FLAG	-	+	-	+
	wild-type probe		mutant probe	

Full unedited gel for Supplemental Figure S5

Iso 1
NM_001137550

Repl.1

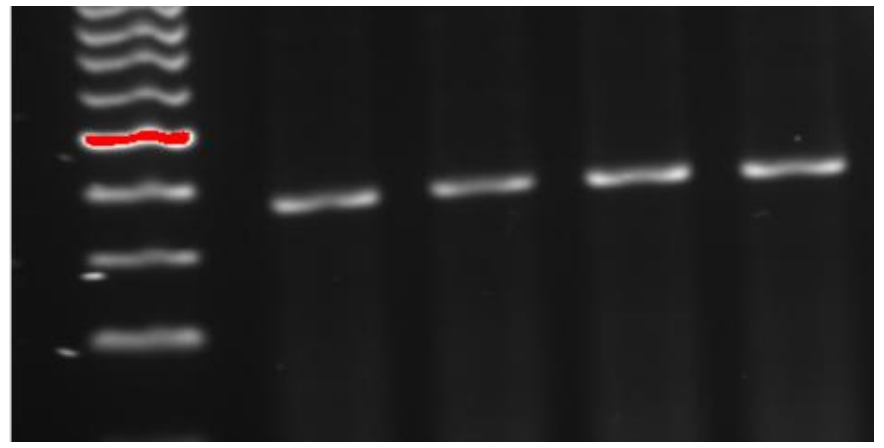

Repl.2

Repl.3

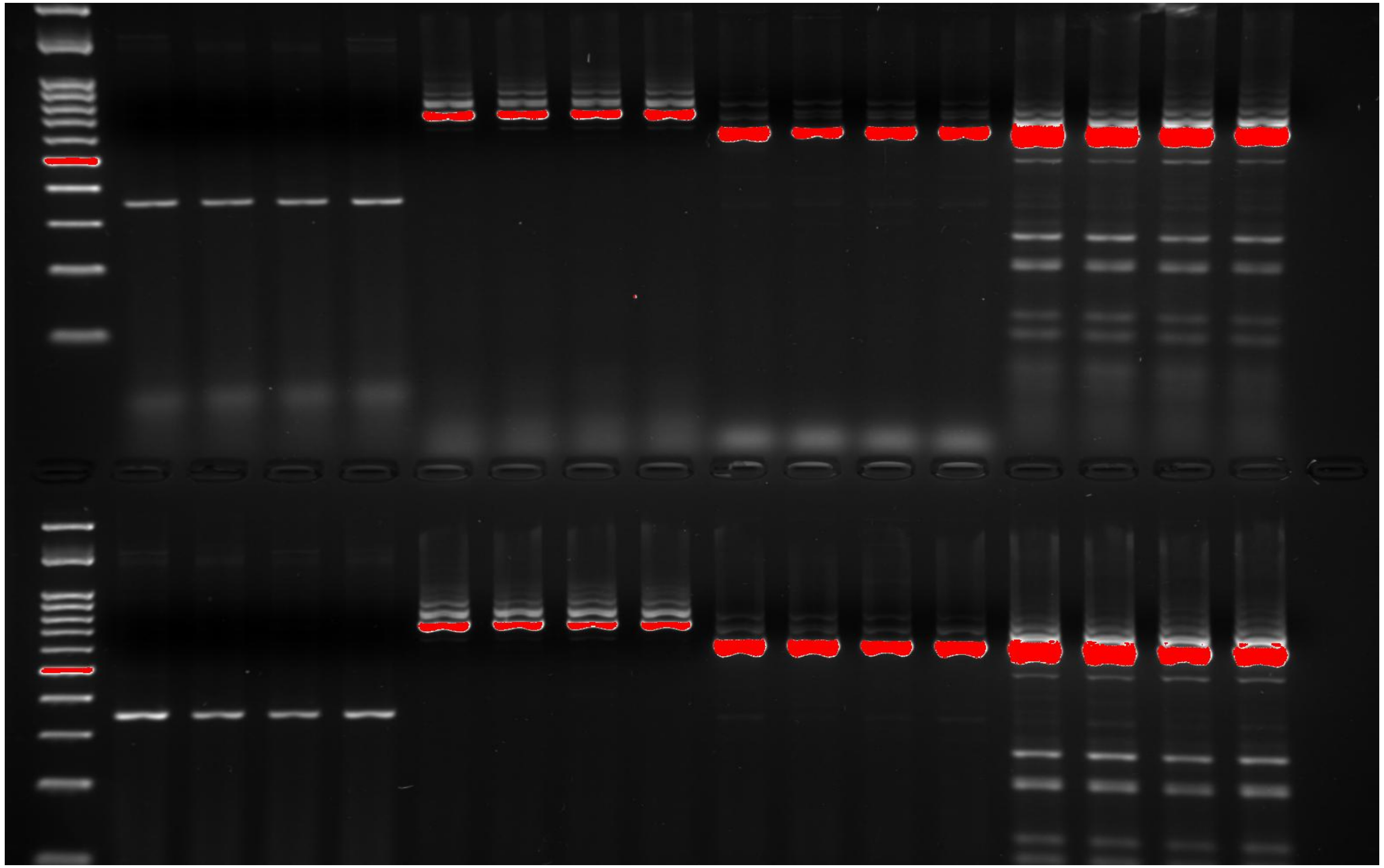

Iso1

Full unedited gel for Supplemental Figure S5

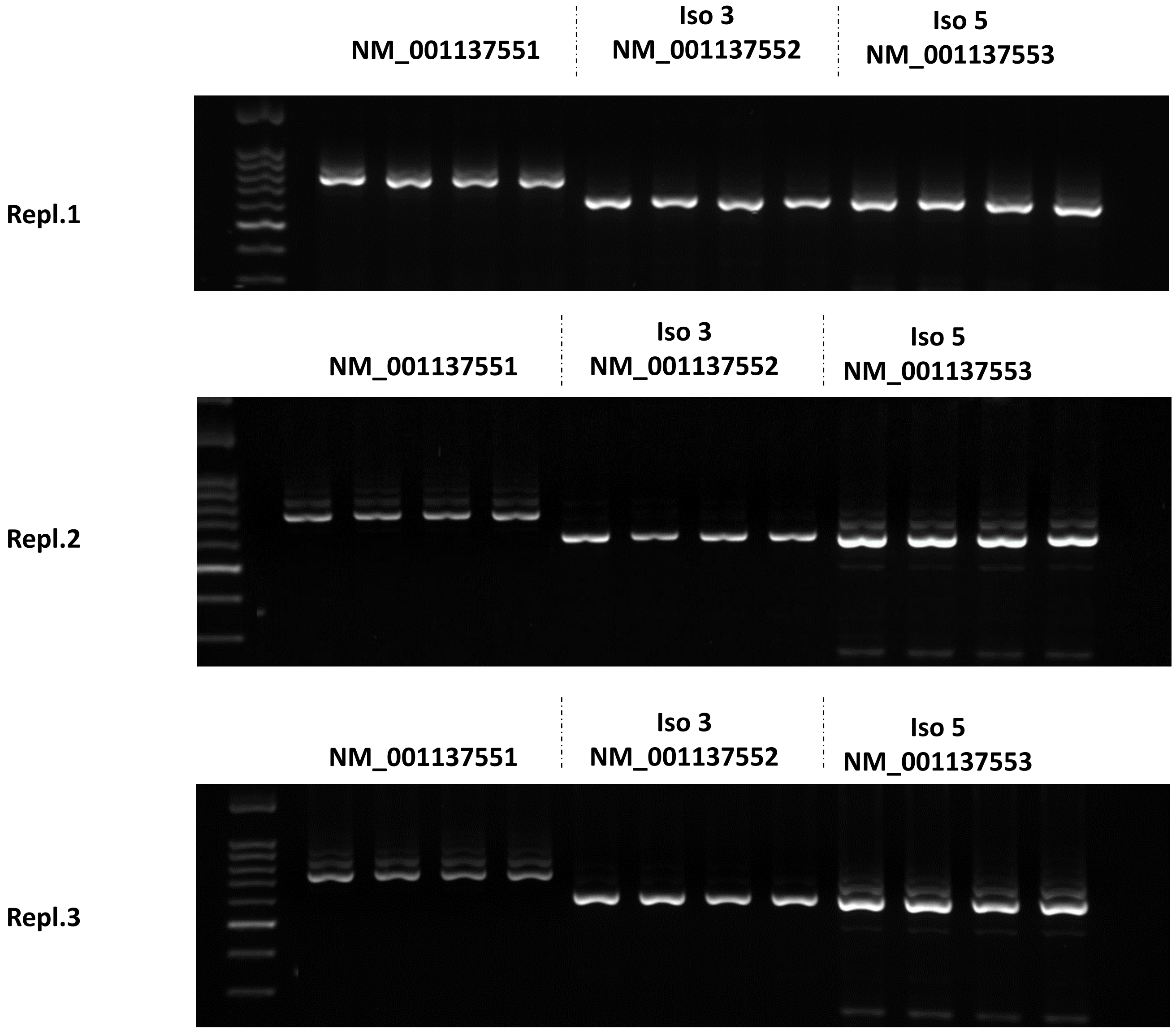

Supplement: Supplementary file 2 [file jci-136-202751-s008.pdf]
